# Determination of main lipids and volatile compounds in unconventional cold‐pressed seed oils through chromatographic techniques

**DOI:** 10.1111/1750-3841.17661

**Published:** 2025-01-19

**Authors:** Francesca Rigano, Federica Vento, Cinzia Cafarella, Emanuela Trovato, Alessandra Trozzi, Paola Dugo, Luigi Mondello

**Affiliations:** ^1^ Department of Chemical, Biological, Pharmaceutical and Environmental Sciences, Messina Institute of Technology University of Messina Messina Italy; ^2^ Department of Chemical, Biological, Pharmaceutical and Environmental Sciences, former Veterinary School, Chromaleont s.r.l. University of Messina Messina Italy

**Keywords:** unconventional seed oils, fatty acids, nutritional indices, novel food, chemometrics

## Abstract

**Abstract:**

The purpose of this study was to characterize unconventional cold‐pressed seed oils (rosehip, strawberry, blackcurrant, carrot, plum, pomegranate, radish, and raspberry) as novel alternative edible oil source. A chemical characterization of different lipid components (total fatty acid composition, triacylglycerols, and vitamin E) and volatiles responsible for the particular aroma of these oils was reported. All the oils showed a content of unsaturated fatty acids, mainly oleic, linoleic, and α‐linolenic acid, that potentially contribute to the prevention of cardiovascular diseases, in the range of 80%–90%. Moreover, an isomer of α‐linolenic acid, namely, punicic acid, was quantified at a level of near to 40% in pomegranate seed oil. Triolein was the most abundant triacylglycerol in most of the analyzed seed oils, with the exception of raspberry and strawberry dominated by trilinolein and pomegranate seed oil, composed for almost 50% of tripunicine. The highest content of vitamin E was found in pomegranate oil (256 mg/100 g), while the lowest amount was found in strawberry (65 mg/100 g). Overall, >300 compounds were identified from volatile profile of oil samples. Among these, aldehydes were the predominant molecule class identified in plum, pomegranate, and strawberry oils, while terpenes were the main volatiles in blackcurrant, carrot, and rosehip oils. Extremely low values were obtained for atherogenicity (0.05–0.10) and thrombogenicity (0.07–0.30) nutritional indices in all the investigated oils. Principal component analysis of the lipid profile was used as strategy to discriminate and classify the samples, highlighting their similarity related to the presence of beneficial compounds.

**Practical Application:**

Unconventional food products can find wide applicability in both cosmetic and food industry as alternative source that harmonize with consumers’ preferences for personal care and nutraceutical purpose. They often address food security, sustainability, and nutritional challenges. Within this context, the chemical characterization of both major (triacylglycerols and total fatty acid composition) and minor components (volatile compounds and vitamin E) was useful to demonstrate that the cold‐pressed seed oils here investigated are rich in essential nutrients. Hence, they can cater to specific dietary needs, thus creating new markets in food tech, agriculture, and biotechnology industries.

## 1 INTRODUCTION

In the last years, consumer interest in the consumption of nutrient‐rich source has increased, with particular attention to nutraceutical alternatives derived from fruits and vegetables (Alves et al., 2020; Kaseke et al., 2020; Sabikhi & Sathish Kumar, 2012; Subedi et al., 2021). Natural cold‐pressed plant seed oils are still poorly diffused and have received slight attention compared to conventional seed oil. From a nutritional point of view, the consumption of seed oils has been associated with the reduction of the risks of various chronic and degenerative diseases, such as cancer, diabetes, and cardiovascular disorders, related to the content of unsaturated fatty acids (UFAs) and bioactive compounds in seeds oils (Bhattacharjya et al., 2021; Cisneros‐Zevallos, 2020).

Chemical composition, content, and availability of these bioactive substances in seed depend on the environmental conditions, agronomic factors, and plant treatment process (Vermaak et al., 2011). Factors intrinsic to food and human body can influence the bioavailability of phytochemicals. For instance, micronutrients such as vitamins and secondary metabolites (e.g., small terpenes) are generally poorly absorbed, largely metabolized, and quickly eliminated. It is therefore advisable that their intake be constant over time to ensure concentrations of the significant metabolites remain high in plasma (Carratù & Sanzini, 2005).

Triglycerides are the compounds that most affect the composition of seed oils, which are also characterized by minority components such as tocopherols, phytosterols, phenols, carotenoids, and phospholipids (Gunstone, 2013; Hernandez, 2005; Przybylski & Eskin, 2011). Fatty acids (FAs) present in seed oils are classified as saturated FAs (SFAs), monounsaturated FAs (MUFAs), and polyunsaturated FAs (PUFAs), mainly represented by linoleic acid (C18:2) and α‐linolenic acid (C18:3), leading compounds of omega‐6 and omega‐3 families, which are very important in preventing cardiovascular diseases. PUFAs are further classified into essential and non‐essential FAs. Their introduction in diets is important since human body does not have the enzymes to synthesize essential FAs belonging to this class of molecules (Orsavova et al., 2015).

Plants also produce secondary metabolites, including volatile organic compounds (VOCs) that possess different biological properties and functions. The main VOCs present in plant seeds are usually classified into terpenoids, amino acid derivatives, FA derivatives, and benzenoid compounds (Dudareva et al., 2013).

Many unconventional edible plant oils are available on the market, but often not enough information is available about their composition and quality. Conversely, it is important to study phytoconstituents of new plant seed oils source in order to fully understand the potential positive effects of their consumption on human health and encourage consumers toward their use. (Alves et al., 2020).

From a legislative point of view, following Regulation (EU) 2015/2283 of the European Parliament and of the European Council on novel foods, the European Commission has asked at the European Food Safety Authority to update and develop scientific and technical advice for the preparation and submission of applications for authorization of novel foods (EFSA, 2021). The EFSA has provided guidance to ensure the quality, safety, and efficacy of these products. In this regard, it has established minimum requirements for the production process and proposed uses, also suggesting including information on nutritional and toxicological value and on the presence of possible allergens.

In this research, blackcurrant, carrot, plum, pomegranate, radish, raspberry, rosehip, and strawberry oils were investigated. Currently, among the analyzed seed oils, only plum seed oil was included in the list of novel food (European Commission, 2017), while the pomegranate seed oil has achieved a positive opinion (European Commission, 2015) but is not yet present on the market, since authorization is required under the Regulation (UE) 2015/2283 before being placed on the market as European food (European Commission, 2023). The profile of the FAs, vitamin E, and triglycerides of the selected cold‐pressed oils was analyzed to evaluate their quality and nutritional properties by using both high‐performance liquid chromatography (HPLC) and gas chromatography (GC) methods. Lipidomic profile was overall investigated by principal component analysis (PCA) to find similarity in composition between the analyzed oils.

Volatile profile was also analyzed by using solid‐phase microextraction (SPME) technique followed by GC coupled to flame ionization detection (FID) and mass spectrometry (MS)  to highlight typical molecules responsible for the aroma of each oil.

Although these oils are already being investigated in literature (Konopka et al., 2023; Liu et al., 2022; Önder et al., 2024; Zlatković et al., 2024), for the best of authors’ knowledge, this is the first research article in which such unconventional seed oils have been compared in term of lipidomic and volatilomic profile in order to valorize them from both nutritional and sensory perspectives. In this regard, nutritional indices were calculated to evaluate the anti‐atherogenic and anti‐thrombogenic activity, and hypocholesterolemic action. Moreover, the analysis of volatile profile led to the identification of >300 compounds, many of which have been identified for the first time in the seed oils analyzed.

From an analytical point of view, the use of complementary identification strategies based on MS spectra and linear retention index (LRI) values in both GC and LC approaches enabled the reliable identification of single compounds. This was especially important for the discrimination of isomeric molecular species, characterized by similar or even identical fragmentation pattern. The proper identification of isomeric species, namely, the capability to distinguish between omega‐3 and omega‐6 FAs, is essential to deduce correct nutritional information.

## 2 MATERIALS AND METHODS

### 2.1 Chemical and reagents

Methanol (MeOH), *n*‐hexane (Hex), 2‐propanol (IPA), acetonitrile (ACN), potassium hydroxide (KOH), and *n*‐heptane were purchased from Merck Life Science (Merck KGaA, Darmstadt). Standards mixtures C7–C30 Saturated Alkanes in Hex (1000 g/mL), C4–C24 fatty acid methyl esters (FAMEs) in Hex (1000 g/mL), trinonanoin, triundecanoin, tritridecanoin, tripentadecanoin, triehptadecanoin, and trinonadecanoin were all provided by Merck Life Science.

### 2.2 Samples

Extra‐virgin olive oil, carrot, strawberry, raspberry, pomegranate, plum, radish, blackcurrant, and rosehip cold‐pressed oils were provided by the company Oleowita (Milicz) during the harvesting years 2022–2023 (for a total of three production lots).

### 2.3 GC‐MS and GC‐FID analysis of FAMEs

For the determination of the total FAs composition by GC‐MS and GC‐FID, 100 mg of each sample was subjected to cold derivatization to convert intact lipids into more volatile FAMEs according to the procedure reported in Lechhab et al. (2022). The GC‐MS analysis was performed on a GC‐QP2030 NX system (Shimadzu) equipped with an AOC‐20i autosampler. A GC‐2010 Plus (Shimadzu) equipped with an FID detector was used for FAMEs quantification. In both cases, the separation of FAMEs was performed on an SLB‐IL‐60i capillary GC column of 30 m × 0.25 mm ID × 0.20 µm df (Merck Life Science). Operating GC‐MS and GC‐FID conditions were the same as those applied in Lechhab et al. (2022). GCMSsolution software (vers. 4.50, Shimadzu) was used for GC‐MS data collection and handling. The identification of FAMEs was performed by using the LIPIDS Mass Spectral Library v1.0 (Shimadzu), with two different identification parameters: mass spectral similarity (over 85%) and LRI, applying a tolerance window of ± 10 units. For calculating LRIs a homologue series of carbon‐saturated FAMEs (C4–C24, Merck Life Science) was injected prior to the samples.

LabSolution software (version 5.92, Shimadzu) was employed for GC‐FID data elaboration. Relative quantification was carried out by integrating each peak and calculating percentage area. All the analyses were performed in triplicate. Atherogenic (AI) and thrombogenic (TI) nutritional indices, and hypocholesterolemic/hypercholesterolemic ratio (h/H) were calculated from identified FAs according to the Equations ((1), (2), (3)) reported by Chen and Liu (2020):

(1) $$\mathrm{AI}=\left[C12:0+\left(4\times C14:0\right)+C16:0\right]/\sum \mathrm{UFA}$$

(2) $$\begin{array}{ccc} \mathrm{TI} & = & \left(C14:0+C16:0+C18:0\right)/[\left(0.5\times \sum \mathrm{MUFA}\right)\\ & & +\left(0.5\times \sum n6-\mathrm{PUFA}\right)+\left(3\times \sum n3-\mathrm{PUFA}\right)\\ & & +\left(n3/n6\right)] \end{array}$$

(3) $$\begin{array}{ccc} h/H & = & \left(\mathrm{cis}18:1+\sum \mathrm{PUFA}\right)\\ & & /\left(C12:0+C14:0+C16:0\right) \end{array}$$

Moreover, after FAs quantification, the peroxidation index (PI) (a measurement of peroxidation susceptibility and peroxidative lipid damage for a particular phospholipid membrane) was calculated by using Equation (4) (Luciano et al., 2013):

(4) $$\begin{array}{ccc} \mathrm{PI} & = & \left(\%\mathrm{dienoic}\times 1\right)+\left(\%\mathrm{trienoic}\times 2\right)+\left(\%\mathrm{tetraenoic}\times 3\right)\\ & & +\left(\%\mathrm{pentaenoic}\times 4\right)+\left(\%\mathrm{hexaenoic}\times 5\right) \end{array}$$

### 2.4 Analysis of vitamin E

Absolute quantification of vitamin E was performed by using the method previously validated by Dugo et al. (2020). Briefly, all samples were dissolved in Hex (1:10, v:v). The analyses were carried out using a HPLC Shimadzu Nexera‐X2 system (Shimadzu), equipped with fluorescence detector (FLD). Tocopherols (α, β, γ, and δ) were separated on an Ascentis Si column (250 × 4.6 mm, L. × i.d., 5 µm particle size (Merck Life Science), operating in isocratic mode with Hex/IPA (99:1, v/v) mobile phase, and flow rate of 1.7 mL/min. Samples were analyzed in triplicate and injection volume was 5 µL. Detector wavelength was 290 nm for excitation and 330 nm for emission. Data acquisition was performed using the LabSolution software (ver. 5.97, Shimadzu).

### 2.5 Analysis of TAGs

For the analysis of TAGs, 20 mg of seed oil was dissolved in IPA, up to a final volume of 10 mL. HPLC analysis was carried out on an HPLC Nexera X2 system (Shimadzu) coupled with an LCMS‐2020 mass spectrometer (Shimadzu) equipped with an atmospheric pressure chemical ionization (APCI) interface (Shimadzu). An Ascentis Express C18 column (10 × 2.1 mm, L. × i.d., 2.7 µm dp, Merck Life Science) was used for the chromatographic separation. Mobile phases consisted of ACN (A) and IPA (B) at a flow rate of 0.5 mL/min in the following linear gradient elution mode: 0–52 min, 0%–70% B, held for 3 min. The oven temperature was set to 35°C. The injection volume was 5 µL.

APCI‐MS parameters were the same as those applied by Oteri et al. (2022). Data acquisition was processed through the LabSolution software (version 5.91, Shimadzu). Three replicates of analyses were acquired, and relative quantification was performed by using the APCI response factor (Beccaria, Moret, et al., 2016; Beccaria, Oteri, et al., 2016). Peak identification was carried out by using an internal dual filter MS library with an embedded LRI database (Oteri et al., 2022). A homologue series of odd chain TAGs from trinonanoin to trinonadecanoin was used to calculate LRIs according to the following equation:

(5) $$\mathrm{LRI}\;=100\left[z+6\;\left(t_{\mathrm{Ri}}-t_{\mathrm{Rz}}\right)/t_{R\left(z+6\right)}-t_{Rz}\right]$$

where *z* is the carbon number of the triglyceride, which elutes before the analyte, *t*_Ri_ is the retention time of the analyte, *t*_Rz_ and *t*_R(z+6)_ are the retention times of the reference triglyceride, which elute before and after the analyte, respectively (Rigano & Mondello, 2019; Rigano et al., 2018).

### 2.6 SPME‐GC‐MS and SPME‐GC‐FID analysis of volatile compounds

For the profiling of the volatile fraction, an SPME fiber coated with divinylbenzene/carboxen/polydimethylsiloxane (DVB/CAR/PDMS) stationary phases (50/30 µm) 1 cm long was used. Sample preparation, GC‐MS, and GC‐FID conditions were the same as reported by Trovato et al. (2023). In detail, 1 mL of oil was conditioned for 5 min at 60°C, and then the fiber was exposed in the sample headspace for 50 min at 60°C. Afterward, the analytes were thermally desorbed for 1 min in the injection port of the GC system at 260°C. Qualitative and semi‐quantitative analyses of volatile components were carried out using a GCMS‐QP2020 NX instrument (Shimadzu) and a GC‐2030 instrument equipped with FID detector (Shimadzu), respectively. In both cases, separation was achieved on a SLB‐5 ms (30 m × 0.25 mm i.d. × 0.25 µm df) capillary column (Merck Life Science). Data handling was supported by GCMS solution ver. 4.30 software (Shimadzu). The identification of volatiles was performed by using the following databases: W11N17 (Wiley11‐Nist17, Wiley) and FFNSC 4.0 (Shimadzu). Peak assignment was performed by evaluating two different identification criteria: MS similarity matching (≥85%) and LRI tolerance windows (±10 units). For calculating LRIs, a homolog series of carbon‐saturated alkanes (C7–C30, Merck Life Science) was used.

As for the GC‐FID analysis, the data were collected by LabSolution software ver. 5.92 (Shimadzu). Quantitative results were expressed in percentage (%) area.

### 2.7 Statistical methods

Statistical analyses were performed with XLstat 2024 software (Lumivero). Analyses were conducted in triplicate and presented as mean ± standard deviation. Data collected were subjected to PCA to check similarities and differences among the samples and evaluate the influence of compound classes and metabolites on the investigated oils. Significant difference within means were subjected to analysis of variance (ANOVA) and Tukey's test at a 95% confidence level (*p* < 0.05).

## 3 RESULTS AND DISCUSSION

### 3.1 Fatty acids profile

FAs profile of the analyzed oils are shown in supporting material (Figures S1–S7). A total of 34 FAs were overall identified, as shown in Table 1. Among UFAs, oleic acid (C18:1n9) and linoleic acid (C18:2n6) are two of the most abundant FAs common to all oils analyzed, found in the range of 20.0%–71.9% and 8.9%–53.4%, respectively, with a significance level of *p* < 0.01. Among the SFAs, palmitic (C16:0) and stearic (C18:0) were the most relevant, quantified in the range of 4.4%–6.4% and 1.4%–3.0%. In particular, palmitic acid was extremely significant for plum and blackcurrant seed oil (*p* < 0.01), and olive oil (*p* < 0.001). Similarly, stearic acid was found to be significantly high for carrot, olive, plum, and rosehip oil (*p* < 0.001). Conversely, pomegranate is the unique oil for which this FA is not statistically relevant (*p* = 0.084).

**TABLE 1:** Fatty acid methyl esters (FAMEs) content of the analyzed oils.

FAMEs	LRI_exp_	LRI_ref_	Pomegranate	Raspberry	Rosehip	Radish	Carrot	Plum	Strawberry	Blackcurrant	Olive
Me. C14:0	1400	1400	0.04 ± 0.00^c^	0.04 ± 0.01^bc^	0.05 ± 0.01^ab^	0.06 ± 0.01^a^	–	–	–	–	–
Me. C16:0	1600	1600	4.82 ± 0.04^cd^	4.46 ± 0.18^d^	5.59 ± 0.05^bc^	5.53 ± 0.05^bc^	4.70 ± 0.03^cd^	6.27 ± 0.02^b^	5.33 ± 0.05^bcd^	6.37 ± 0.08^b^	13.43 ± 1.68^a^
Me. C16:1n9	1608	1603	–	–	–	0.02 ± 0.01^c^	0.07 ± 0.00^b^	–	0.03 ± 0.01^c^	–	0.08 ± 0.01^a^
Me. C16:1n7	1620	1616	0.09 ± 0.01^c^	0.08 ± 0.01^c^	0.14 ± 0.01^c^	0.13 ± 0.01^c^	0.20 ± 0.01^c^	1.17 ± 0.01^a^	0.15 ± 0.01^c^	0.08 ± 0.01^c^	0.91 ± 0.01^b^
Me. C17:0	1700	1700	0.03 ± 0.01^b^	0.03 ± 0.00^b^	0.04 ± 0.00^b^	0.03 ± 0.00^b^	0.03 ± 0.00^b^	0.04 ± 0.01^b^	–	–	0.12 ± 0.04^a^
Me. C17:1n7	1718	1719	–	0.04 ± 0.00^c^	–	0.03 ± 0.00 ^cd^	0.05 ± 0.0^c^	0.11 ± 0.01^b^	–	–	0.19 ± 0.05^a^
Me. C18:0	1800	1800	2.61 ± 0.03^bc^	2.24 ± 0.04^d^	2.91 ± 0.06^a^	2.19 ± 0.03^d^	1.38 ± 0.07^e^	1.51 ± 0.02^e^	2.45 ± 0.04^cd^	2.77 ± 0.04^ab^	3.05 ± 0.11^a^
Me. C18:1n12	1809	1802	–	–	–	–	9.83 ± 0.19^a^	–	–	–	–
Me. C18:1n9	1816	1810	20.04 ± 0.14^i^	23.46 ± 0.13^h^	52.76 ± 0.24^d^	31.10 ± 0.39^e^	69.69 ± 0.47^c^	71.90 ± 0.05^a^	26.57 ± 0.13^f^	25.58 ± 0.06^g^	70.26 ± 0.11^b^
Me. C18:1n7	1825	1820	0.57 ± 0.00^f^	0.57 ± 0.01^f^	0.70 ± 0.02^e^	1.15 ± 0.04^d^	2.23 ± 0.04^a^	1.52 ± 0.01^c^	0.63 ± 0.04^ef^	0.65 ± 0.02^ef^	2.03 ± 0.16^b^
Me. C18:2n6	1857	1848	29.07 ± 0.24^d^	50.00 ± 0.04^b^	32.72 ± 0.04^c^	16.90 ± 0.10^e^	8.94 ± 007^f^	17.26 ± 0.02^e^	48.61 ± 0.14^b^	53.42 ± 0.31^a^	7.76 ± 2.26^f^
Me. C18:3n6	1865	1858	–	–	–	–	–	–	–	3.10 ± 0.07^a^	–
Me. C18:3n3	1908	1900	0.03 ± 0.01^h^	18.16 ± 0.13^a^	3.55 ± 0.09^e^	14.51 ± 0.10^c^	0.24 ± 0.03^g^	0.06 ± 0.00^h^	14.95 ± 0.06^b^	5.00 ± 0.07^d^	0.61 ± 0.03^f^
Me. C18:4n3	1917	1909	–	–	–	–	–	–	–	0.44 ± 0.03^a^	–
Me. C20:0	2000	2000	0.33 ± 0.01^c^	0.23 ± 0.03^d^	0.35 ± 0.01^c^	0.78 ± 0.03^a^	0.33 ± 0.01^c^	0.10 ± 0.01^e^	0.52 ± 0.02^b^	0.26 ± 0.01^d^	0.5 ± 0.03^b^
Me. C20:1n9	2018	2008				9.44 ± 0.10^a^	0.43 ± 0.01^c^	0.06 ± 0.00^e^	0.19 ± 0.02^d^	1.35 ± 0.04^b^	0.02 ± 0.00^ef^
Me. C20:1n7	2029	2015	0.37 ± 0.02^a^	0.22 ± 0.02^c^	0.19 ± 0.01^d^	0.20 ± 0.02 ^cd^	–	–	–	–	0.31 ± 0.04^b^
Me. C20:2n6	2062	2055	–	–	–	0.43 ± 0.03^a^	–	–	–	0.16 ± 0.01^b^	–
Me. C20:3n3	2118	2109	–	–	–	0.13 ± 0.01^a^	–	–	–	–	–
Me. C22:0	2200	2200	0.79 ± 0.20^a^	0.35 ± 0.04^b^	0.73 ± 0.04^a^	0.34 ± 0.02^b^	0.83 ± 0.01^a^	–	0.42 ± 0.03^b^	0.47 ± 0.01^b^	0.68 ± 0.14^a^
Me. C22:1n9	2221	2217	–	–	–	15.03 ± 0.19^a^	0.73 ± 0.00^b^	–	–	0.19 ± 0.01^c^	–
C18:3n5 CLnA (punicic acid)^*^	2229	–	37.71 ± 0.45^a^	–	–	–	–	–	–	–	–
Me. C22:1n7	2233	2229	–	–	–	0.05 ± 0.01^a^	–	–	–	–	–
C18:3n5 (catalpic acid)^*^	2255	–	2.18 ± 0.21^a^	–	–	–	–	–	–	–	–
Me. C22:2n6	2269	2262	–	–	–	0.09 ± 0.01^a^	–	–	–	–	–
C18:3n5 (β‐eleostearic acid)^*^	2280	–	0.81 ± 0.16^a^	–	–	–	–	–	–	–	–
Me. C23:0	2300	2300	0.02 ± 0.00^b^	–	0.03 ± 0.01^a^	–	–	–	–	–	–
C18:3n5^*^	2330	–	0.35 ± 0.13^a^	–	–	–	–	–	–	–	–
Me. C24:0	2400	2400	0.14 ± 0.01^ef^	0.12 ± 0.02^f^	0.24 ± 0.02^c^	0.45 ± 0.03^a^	0.32 ± 0.00^b^		0.16 ± 0.01^de^	0.17 ± 0.00^d^	0.07 ± 0.01^g^
Me. C24:1n9	2424	2420	–	–	–	1.42 ± 0.07^a^	–	–	–	–	–

*Note*: Values are expressed as mean percentage area ± standard deviation (SD) (*n* = 3). Data with different superscript letters in the same row have different significance level at *p* < 0.05.

Abbreviation: LRI, linear retention index.

* M.T. Boroushaki et al. (2016).

A high percentage of α‐linolenic acid (C18:3n3) was also found in raspberry (18.2%), radish (14.5%), and strawberry (15.0%), followed by rosehip (3.5%) and blackcurrant (5.0%) seed oils, and it was extremely significant for all the oils analyzed (*p* < 0.001). Some FAs are characteristic of few seed oils; for example, punicic acid (CLnA), catalpic acid, and β‐eleostearic acid (omega‐5 PUFAs, isomers of conjugated α‐linolenic acid) were found only in pomegranate seed oil. Their identification was confirmed by comparing experimental mass spectra with literature data (Boroushaki et al., 2016), and they were found to have a percentage area of 37.71%, 2.18%, and 0.81%, respectively. These C18:3n5 acids were extensively investigated for their beneficial effects on human health, such as antioxidant, anti‐cancer, anti‐obesity, and anti‐inflammatory activities, as well as positive actions on regulating diabetes and body immunity (Du et al., 2024). In radish seed oil, eicosenoic acid (C20:1n9) and erucic acid (C22:1n9) were detected with high percentage areas of 9.44% and 15.03%, respectively. In addition, this is the only seed oil in which nervonic acid (C24:1n9) was encountered. It is noteworthy as the high level of erucic acid is connected to well‐known negative effect on the cardiovascular system (Galanty et al., 2023), so that a regulation was introduced in Europe establishing the maximum level of 5% (*w:w*) for erucic acid in fats and oils and foods containing vegetable oils and fats (Council of the European Communities, 1976).

In carrot seed oil, a high content (9.96%) of petroselinic acid (C18:1n12), typical of food products belonging to the *Apiaceae* family (Hajib et al., 2023), was detected. Plum seed oil is the unique oil in which a significant amount of palmitoleic acid (C16:1n7) content (1.17%) was found. Finally, γ‐linolenic acid (C18:3n6) was detected only in blackcurrant seed oil with a percentage area of 3.10% and stearidonic acid (C18:4n3).

The obtained semi‐quantitative results were used to calculate SFA, MUFA, PUFA, and UFA content, omega‐3 and omega‐6 total %, their ratios, and nutritional indices according to Equations ((1), (2), (3)). From a nutritional point of view, the most interesting results were observed for pomegranate, raspberry, strawberry, and blackcurrant seed oils, for which the PUFA content accounted in the range of 62.12%–70.15%, (Table 2), with a *p* value < 0.001 for all the oils.

**TABLE 2:** FAs identified in oils analyzed, along with FA classes and ratio, and nutritional indices.

FA class	Pomegranate	Raspberry	Rosehip	Radish	Carrot	Plum	Strawberry	Blackcurrant	Olive
**SFA**	8.78 ± 0.31^def^	7.47 ± 0.007^g^	9.94 ± 0.15^bc^	9.38 ± 0.15^bcd^	7.58 ± 0.41^fg^	7.92 ± 0.02^efg^	8.88 ± 0.14^cde^	10.04 ± 0.14^b^	17.84 ± 1.73^a^
**MUFA**	21.07 ± 0.17^h^	24.37 ± 0.11^g^	53.79 ± 0.20^e^	58.57 ± 0.07^d^	83.24 ± 0.51^a^	74.76 ± 0.04^b^	27.57 ± 0.07^f^	27.85 ± 0.02^f^	73.80 ± 0.55^c^
**PUFA**	70.15 ± 0.52^a^	68.16 ± 0.14^b^	36.27 ± 0.05^d^	32.06 ± 0.11^e^	9.18 ± 0.07^g^	17.32 ± 0.02^f^	63.56 ± 0.08^c^	62.12 ± 0.14^c^	8.36 ± 2.28^g^
**UFA**	91.22 ± 0.67^bc^	92.53 ± 0.07^a^	90.06 ± 0.15^c^	90.63 ± 0.15^c^	92.42 ± 0.53^a^	92.08 ± 0.02^ab^	91.13 ± 0.14^bc^	89.97 ± 0.13^c^	82.16 ± 1.73^d^
**SFA/MUFA**	0.42 ± 0.01^a^	0.31 ± 0.00^c^	0.18 ± 0.00^e^	0.16 ± 0.00^f^	0.09 ± 0.01^g^	0.11 ± 0.00 ^g^	0.32 ± 0.01^c^	0.36 ± 0.00^b^	0.24 ± 0.02^d^
**PUFA/SFA**	7.99 ± 0.27^b^	9.12 ± 0.10^a^	3.65 ± 0.05^e^	3.42 ± 0.06^e^	1.22 ± 0.09^g^	2.19 ± 0.01^f^	7.16 ± 0.12^c^	6.19 ± 0.10^d^	0.48 ± 0.17^h^
**SFA/UFA**	0.10 ± 0.00^bc^	0.08 ± 0.00^c^	0.11 ± 0.300^b^	0.10 ± 0.00^b^	0.08 ± 0.01^c^	0.09 ± 0.00^c^	0.10 ± 0.00^bc^	0.11 ± 0.00^b^	0.22 ± 0.03^a^
**n3**	0.03 ± 0.00^h^	18.16 ± 0.13^a^	3.55 ± 0.09^e^	14.64 ± 0.06^c^	0.24 ± 0.03^g^	0.06 ± 0.00^h^	14.95 ± 0.06^b^	5.44 ± 0.10^d^	0.61 ± 0.03^f^
**n6**	29.07 ± 0.27^d^	50.00 ± 0.04^b^	32.72 ± 0.04^c^	17.42 ± 0.05^e^	8.94 ± 0.07^f^	17.26 ± 0.02^e^	48.61 ± 0.14^b^	56.68 ± 0.24^d^	7.76 ± 2.26^f^
**n3/n6**	0.001 ± 0.00 ^g^	0.36 ± 0.00^b^	0.11 ± 0.00^d^	0.84 ± 0.00^a^	0.03 ± 0.00^f^	0.004 ± 0.000^g^	0.31 ± 0.00^c^	0.10 ± 0.00^de^	0.08 ± 0.02^e^
**AI**	0.10 ± 0.00^b^	0.05 ± 0.00^d^	0.06 ± 0.00^cd^	0.06 ± 0.00^cd^	0.05 ± 0.00^d^	0.07 ± 0.00^c^	0.06 ± 0.00^cd^	0.07 ± 0.00^c^	0.16 ± 0.02^a^
**TI**	0.30 ± 0.00^b^	0.07 ± 0.00^e^	0.16 ± 0.00cd	0.09 ± 0.00^e^	0.13 ± 0.01^d^	0.17 ± 0.00^c^	0.09 ± 0.00^e^	0.16 ± 0.00^cd^	0.39 ± 0.05^a^
**PI**	111.23 ± 17.52^a^	86.32 ± 1.34^ab^	39.82 ± 0.31^a^	46.70 ± 0.11^a^	9.42 ± 0.09^b^	17.38 ± 0.31^a^	78.51 ± 0.45^a^	71.10 ± 0.84^a^	–
**h/H**	10.11 ± 0.13^g^	20.36 ± 0.79^a^	15.79 ± 0.15^c^	11.18 ± 0.18^f^	16.80 ± 0.02^b^	14.23 ± 0.05^d^	16.91 ± 0.20^b^	13.19 ± 0.22^e^	5.95 ± 0.91^h^

*Note*: Data with different superscript letters in the same row have different significance level at *p* < 0.05.

Abbreviations: AI, atherogenic index; FA, fatty acids; h/H, hypo‐/Hypercholesterolemic ratio; MUFA, monounsaturated fatty acids; n3, n3‐polyunsaturated fatty acids; n3/n6, n3‐polyunsaturated fatty acids/n6‐polyunsaturated fatty acids ratio; n6, n6‐polyunsaturated fatty acids; PI, peroxidation index; PUFA, polyunsaturated fatty acids; PUFA/SFA, polyunsaturated/saturated fatty acid ratio; SFA, saturated fatty acids; SFA/MUFA, saturated/monounsaturated fatty acid ratio; SFA/UFA, saturated/unsaturated fatty acid ratio; TI, thrombogenic index.

All plant seed oils presented an SFA content in the range of 7%–10%. In particular, this parameter was significantly high for olive oil (*p* < 0.001) and not significant for pomegranate (*p* = 0.158) and radish (*p* = 0.658) seed oils. Raspberry, pomegranate, and strawberry oils presented the higher ratio PUFA/SFA, important for the prevention of cardiovascular pathologies (Chen & Liu, 2020). Furthermore, the ratio values were found significant for all the oils analyzed (*p* < 0.001). Despite it being known that low values of AI and TI indicate better nutritional food quality, no institution has yet identified recommended values for these indices. In the present study, very low AI and TI values were attained for all the analyzed oils, in the range of 0.05%–0.10% and 0.07%–0.30%, respectively. More in detail, the lowest AI value of 0.05 was obtained for raspberry and carrot seed oils, characterized by the highest UFA amount. In particular, AI value showed a not significant relevance for radish, raspberry, rosehip, and strawberry oils, having a *p* > 0.05). Raspberry also presented the lowest TI value (*p* < 0.05), due to the highest omega‐3 content. Furthermore, the h/H ratio provided good results for all seed oils (10.11%–20.36%) with the highest value calculated for raspberry and strawberry seed oils (20.36% and 16.91%, respectively). In particular, carrot is the unique oil for which this ratio was not statistically significant (*p* = 0.617).

**FIGURE 1:**
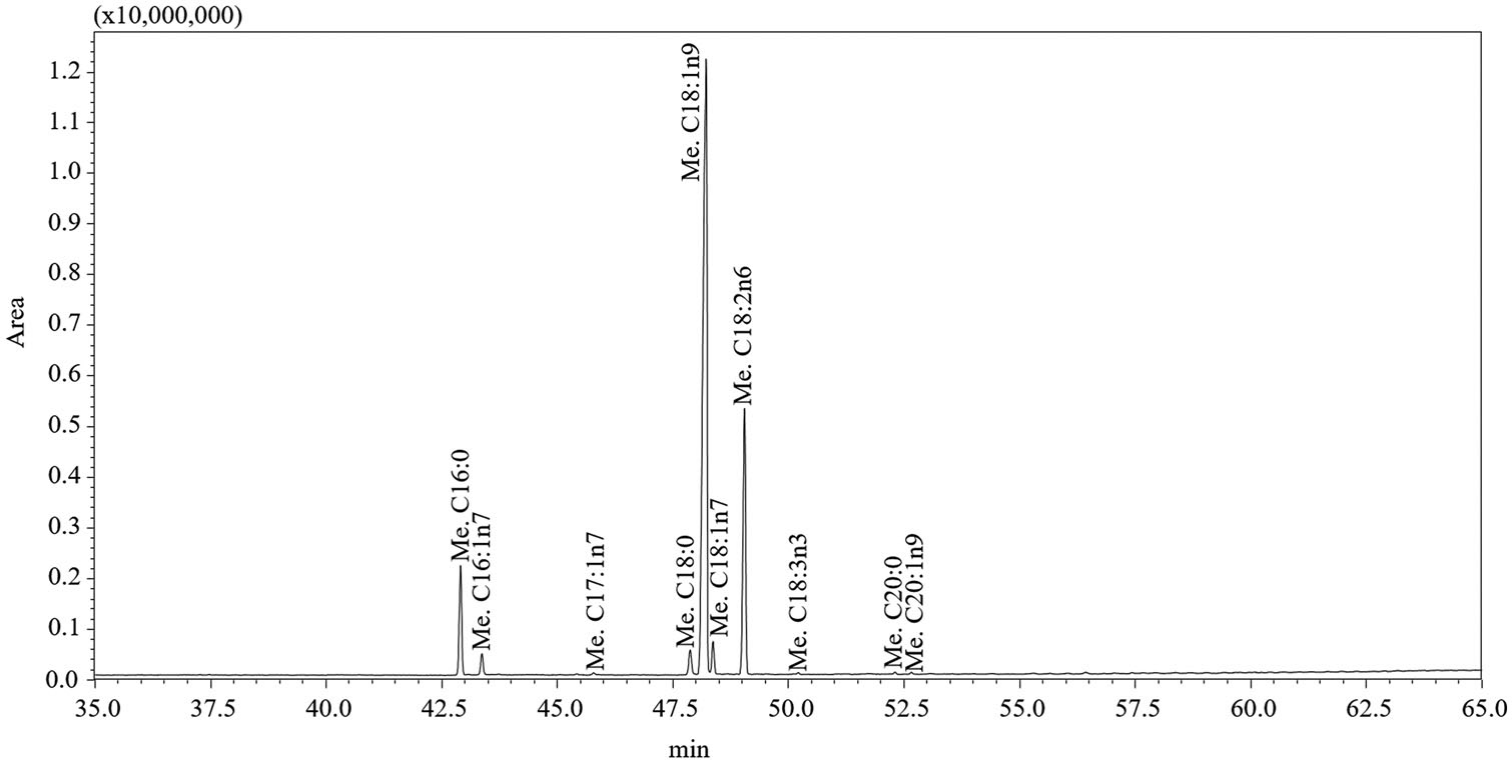
Fatty acids profile of plum seed oil, acquired by GC‐FID analysis.

Figure 1 shows the chromatographic profile obtained for plum seed oil, which has been included in the list of novel food, and it has been identified as vegetable fat to use in frying process (Chang et al., 2019). The MUFA content of plum seed oil (74.76%) is comparable to extra virgin olive oil (EVOO) (mean of 73.80% over more the 200 Italian EVOOs previously analyzed), thus attributing it some healthy aspects normally associated with EVOO. In addition, a high content of MUFAs combined with a low amount of PUFAs is preferable for frying because it ensures an excellent resistance to oxidation (Lozano‐Castellòn et al., 2022), as demonstrated by the lowest PI value obtained for plum seed oil.

### 3.2 Vitamin E content

Total tocopherol (vitamin E) and its vitamers α‐, β‐, ɣ‐, and δ‐tocopherols were determined in triplicates by HPLC‐FLD. As concern total vitamin E, pomegranate seed oil presented the highest content (256.11 mg/100 g), followed by raspberry seed oil (192.35 mg/100 g) and carrot seed oil (145.39 mg/100 g). On the contrary, the lowest value of vitamin E was found in strawberry (64.80 mg/100 g) and radish (65.78 mg/100 g) seed oils, as reported in Table 3. Among the vitamin E components, γ‐tocopherol was the most representative vitamer in all plant seed oils, mostly abundant in pomegranate oil (223.73 mg/100 g), with the exception of rosehip oil, in which α‐tocopherol was the predominant vitamer and accounted for 66.93 mg/100 g. α‐Tocopherol was found in discrete amount also in carrot, raspberry, and blackcurrant (48.61 mg/100 g, 57.48 mg/100 g, and 44.15 mg/100 g, respectively). This vitamer showed a significance level *p* < 0.001 for all oils, with the exception of plum seed oil (*p* = 0.538). β‐Tocopherol was detected mainly in carrot seed oil (34.42 mg/100 g), and few amounts were detected also in raspberry (7.03 mg/100 g), rosehip (4.19 mg/100 g), and blackcurrant (3.65 mg/100 g) oils (*p* < 0.001). Furthermore, the highest content of δ‐tocopherol was detected in raspberry, blackcurrant, and pomegranate seed oils (40.97 mg/100 g, 10.23 mg/100 g and 8.93 mg/100 g, respectively). ANOVA showed that δ‐tocopherol was not significant for plum (*p* = 0.05) and radish seed oil (*p* = 0.790).

**TABLE 3:** Tocopherols and vitamin E total detected in the analyzed oils by LC‐FLD analyses.

Plant seed oil	α‐Tocopherol mg/100 g	β‐Tocopherol mg/100 g	γ‐Tocopherol mg/100 g	δ‐Tocopherol mg/100 g	Vitamin E total mg/100 g
**Pomegranate**	25.46 ± 0.26^f^	–	223.73 ± 0.60^a^	8.93 ± 0.28^b^	256.11 ± 1.13^a^
**Raspberry**	57.48 ± 0.92^c^	7.03 ± 0.11^b^	86.87 ± 1.14^c^	40.97 ± 0.86^a^	192.35 ± 3.04^ab^
**Rosehip**	66.93 ± 0.28^b^	4.19 ± 0.06^bc^	46.29 ± 0.21^f^	2.22 ± 0.12^cde^	119.63 ± 0.67^c^
**Radish**	3.11 ± 0.04^h^	–	58.83 ± 0.20^d^	3.84 ± 0.06^c^	65.78 ± 0.30^d^
**Carrot**	48.61 ± 0.13^d^	34.42 ± 0.08^a^	61.88 ± 0.70^h^	0.47 ± 0.03^de^	145.39 ± 0.95^b^
**Plum**	10.37 ± 0.09^g^	–	95.25 ± 0.44^b^	2.90 ± 0.05^cd^	108.52 ± 0.59^c^
**Strawberry**	8.78 ± 0.09^g^	–	51.61 ± 0.19^e^	4.40 ± 0.12^c^	64.80 ± 0.40^d^
**Blackcurrant**	44.15 ± 0.17^e^	3.65 ± 0.10^c^	48.52 ± 0.31^e^	10.23 ± 0.25^b^	106.56 ± 0.84^c^
**Olive**	164.35 ± 25.20^a^	2.31 ± 0.38^d^	8.47 ± 1.26^g^	‐	175.14 ± 26.84^b^

*Note*: Values are expressed as mean percentage area ± standard deviation (SD) (*n* = 3). Data with different superscript letters in the same row have different significance level at *p* < 0.05.

### 3.3 Triacylglycerol profile

**FIGURE 2:**
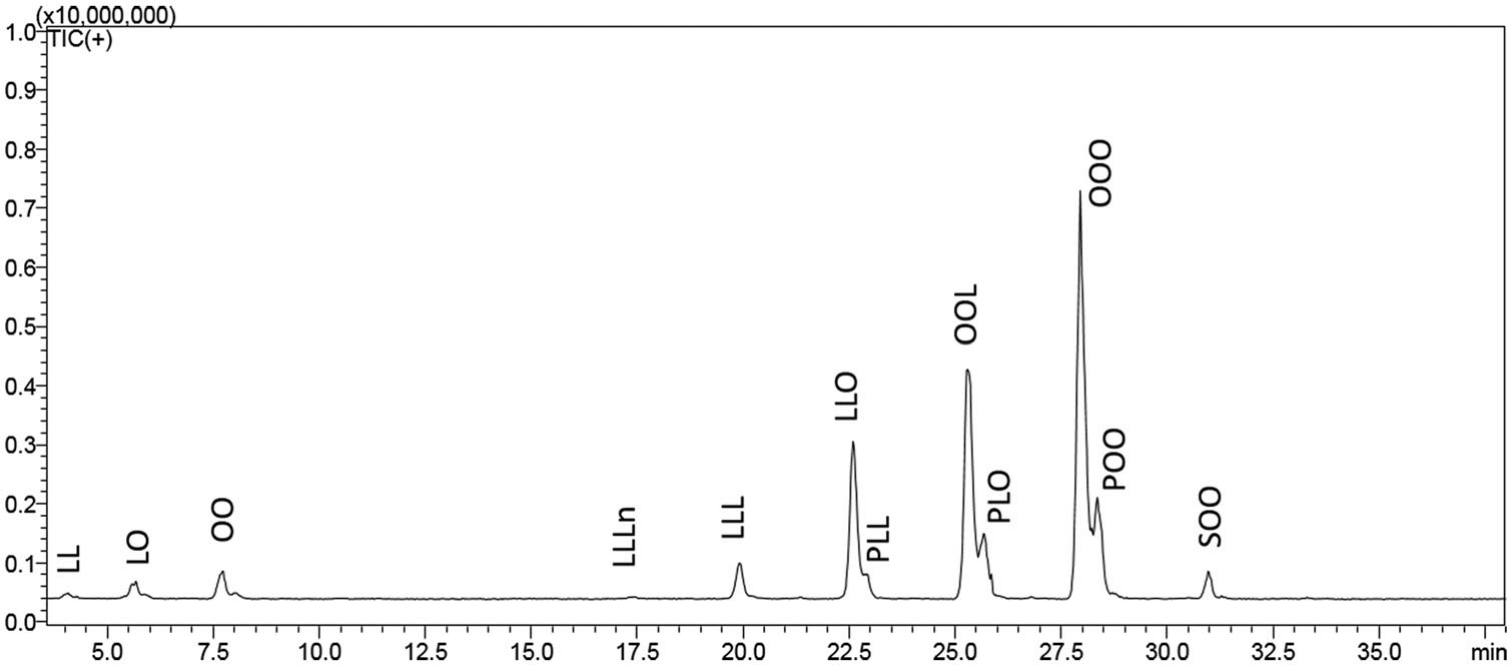
Triacylglycerols profile of plum seed oil, acquired by HPLC‐MS analysis. FA abbreviation: L, Linoleic acid C18:2n6; O, Oleic acid C18:1 n9; Ln, linolenic acid, C18:3n3; P, Palmitic acid, C16:0; S, Stearic acid C18:0.

Figure 2 shows the TAG profile of plum seed oil, including minor diacylglycerol (DAG) peaks. The TAG profile of the other analyzed seed oils is shown in Figures S8–S14. For a rapid, automatic, and reliable identification, an automatic dual filter strategy was employed, which exploits the complementarity between LRI and a homemade MS spectral library (Rigano et al., 2018), leading to identification of 34 compounds overall. An example of identification is shown in Figures S15–S16, along with the interpretation of main MS fragments enabling the reliable identification of the FA composition of each TAG. Table 4 reports the list of all identified compounds, along with calculated LRI in comparison with tabulated values and % areas. A maximum difference of 12 LRI units was obtained for 23 out of 34 identified TAGs, thus confirming the robustness of the LRI approach, recently implemented in LC. The remaining 11 compounds were not present in the database and were identified through MS spectra and literature information (Boroushaki et al., 2016).

**TABLE 4:** Triglycerides composition of the analyzed seed oils, along with experimental linear retention index (LRI exp.) and reference linear retention index (LRI ref.).

DAGs and TAGs	LRI_exp_	LRI_ref_	Pomegranate	Raspberry	Rosehip	Radish	Carrot	Plum	Strawberry	Blackcurrant	Olive
**cLncLn**	2659	–	2.70 ± 0.14^a^								
**LL**	2878	–	1.88 ± 0.31^a^	0.67 ± 0.24^cd^	0.92 ± 0.04^cd^	1.96 ± 0.69^b^		0.40 ± 0.02^d^	0.91 ± 0.09^c^	1.19 ± 0.08^b^	
**LO**	3049	–	1.28 ± 0.16^ab^	0.44 ± 0.38^de^	0.70 ± 0.13^cd^	1.87 ± 0.52^a^		1.60 ± 0.09^cd^	1.15 ± 0.21^bc^	0.81 ± 0.15 ^cd^	
**OO**	3307	–		1.49 ± 0.18^c^	0.67 ± 0.05^de^	1.93 ± 0.24^b^	1.97 ± 0.55^a^	1.79 ± 0.13^d^	0.57 ± 0.19^f^	0.83 ± 0.05^e^	
**C_22:1_L**	3418	–				0.71 ± 0.52^a^					
**cLncLncLn**	3696	–	46.99 ± 1.91^a^								
**LnLnLn**	3672	3669		7.70 ± 21.52^a^	0.76 ± 0.04^d^				4.69 ± 0.96^b^	1.40 ± 0.26^c^	
**LnLnL**	3835	3842		12.11 ± 2.13^a^	4.37 ± 0.27^c^	0.47 ± 0.22^e^			9.68 ± 1.25^b^	1.60 ± 0.26^d^	
**cLncLnL**	3865	–	5.15 ± 0.29^a^								
**LLLn**	4010	4005		13.36 ± 1.85^a^	6.07 ± 0.14^c^	0.75 ± 0.29^e^		0.09 ± 0.00^f^	11.84 ± 1.58^b^	4.03 ± 0.20^d^	
**LnLnO**	4020	4015		2.59 ± 0.35^b^	0.82 ± 0.14^b^	1.90 ± 0.91^b^			3.45 ± 1.11^ab^	3.73 ± 0.07^a^	
**LLcLn**	4026	–	7.92 ± 0.21^a^								
**LnPLn**	4048	4040		1.21 ± 0.23^a^	0.24 ± 0.03^b^				1.23 ± 0.12^a^		
**PcLncLn**	4064	–	4.08 ± 0.49^a^								
**LLL**	4169	4160	6.28 ± 0.21^d^	19.00 ± 2.36^a^	15.91 ± 0.33^b^	2.27 ± 1.23^f^	4.49 ± 0.04^a^	2.91 ± 0.08^f^	17.40 ± 1.25^a^	15.38 ± 0.48^c^	
**LnLO**	4190	4186		4.43 ± 0.89^b^	2.41 ± 0.27^bc^	2.85 ± 1.04^bc^			7.90 ± 1.36^a^	2.50 ± 0.14^c^	
**PoLL**	4204	4206		0.96 ± 0.14^b^					0.35 ± 0.17^c^	1.99 ± 0.01^a^	
**cLnLP**	4201	–	1.29 ± 0.09^a^								
**LnLP**	4228	4229			0.68 ± 0.04^d^	1.40 ± 0.50^b^			2.52 ± 0.38^a^	1.07 ± 0.11^c^	
**cLncLnS**	4255	–	2.71 ± 0.22^a^								
**LLO**	4343	4339	7.90 ± 0.54^e^	15.86 ± 2.27^c^	15.88 ± 0.41^bc^	6.20 ± 2.27^e^	5.64 ± 0.36^f^	13.9 ± 0.87^d^	16.40 ± 1.58^ab^	17.40 ± 1.42^a^	5.23 ± 0.40^f^
**LLP**	4372	4358	2.05 ± 0.23 ^g^	4.16 ± 0.98^d^	4.64 ± 0.54^e^	8.65 ± 2.05^a^	2.67 ± 0.10^f^	1.05 ± 0.19^h^	7.05 ± 1.27^b^	5.27 ± 0.72^c^	1.14 ± 0.09^h^
**POLn**	4395	4389							1.76 ± 0.19^a^		
**OOcLn**	4410	–	0.38 ± 0.05^a^								
**OOL**	4530	4522	3.03 ± 0.08 ^g^	5.21 ± 1.12^f^	7.68 ± 0.09^de^	14.61 ± 2.01^c^	6.14 ± 0.49^f^	23.65 ± 1.87^a^	5.28 ± 1.02^ef^	7.83 ± 0.59^d^	21.12 ± 1.62^b^
**PLO**	4558	4548	2.41 ± 0.24 ^g^	3.89 ± 1.02^ef^	4.95 ± 0.38^b^	4.47 ± 0.99b^cd^	3.66 ± 0.28^f^		4.18 ± 0.19^cde^	4.58 ± 0.42^bc^	6.75 ± 0.52^a^
**SLL**	4590	4575					4.91 ± 0.07^a^				5.23 ± 0.40^a^
**PPL**	4595	4584						4.83 ± 0.69^a^	0.63 ± 0.16^b^		0.76 ± 0.06^a^
**SOLn**	4603	–			0.37 ± 0.02^a^						
**OOO**	4739	4732	3.91 ± 0.11^e^	4.10 ± 1.89^e^	25.30 ± 1.62^c^	19.28 ± 2.58^d^	54.65 ± 0.11^a^	41.35 ± 1.41^b^	1.10 ± 0.20^f^	23.2 ± 0.60^c^	44.20 ± 3.40^b^
**POO**	4767	4763	1.29 ± 0.10^e^	1.72 ± 0.63^e^	4.54 ± 1.06^c^	7.13 ± 1.97^c^	10.23 ± 0.84^b^	6.62 ± 0.99^c^	1.27 ± 0.20^e^	4.27 ± 0.81^d^	21.77 ± 1.67^a^
**C_22:1_LO**	4895	4891				8.84 ± 1.74^a^					
**SOO**	4943	4938	0.50 ± 0.05^e^	1.00 ± 0.28^d^	3.00 ± 0.21^b^	2.27 ± 1.25^b^	5.60 ± 0.11^a^	1.74 ± 0.12^c^	0.59 ± 0.12^e^	2.84 ± 0.22^b^	3.27 ± 0.25^a^
**C_22:1_OO**	5091	5088				6.09 ± 2.21^a^					
**C_22:1_OP**	5096	–				3.38 ± 1.32^a^					
**C_22:1_OG**	5220	5228				2.17 ± 1.98^a^					
**C_22:1_OS**	5255	5267				0.53 ± 2.13^a^					
**C_22:1_GG**	5298	–				0.70 ± 0.65^a^					

*Note*: Values are expressed as mean percentage area ± standard deviation (SD) (*n* = 3). Data with different superscript letters in the same row have different significance level at *p* < 0.05. FA abbreviation: cLn, Punicic acid C18:3n5; L, Linoleic acid C18:2n6; O, Oleic acid C18:1 n9; C22:1, erucic acid; Ln, linolenic acid, C18:3n3; P, Palmitic acid, C16:0; Po, Palmitoleic acid, C16:1n7; S, Stearic acid C18:0; G, Gadoleic acid, C20:1n9.

As for semi‐quantitative results, the most representative TAG in rosehip, radish, carrot, plum, and blackcurrant seed oils was triolein (OOO) (*p* < 0.001). In raspberry and strawberry, the most abundant TAG was trilinolein (LLL) (19.00% and 17.40%, respectively). This TAG had a significant statistical impact for all the oils (*p* < 0.001). Furthermore, strawberry is the unique oil in which palmitin‐olein‐linolenin (POLn) was detected (1.76%). Pomegranate seed oil was composed of almost 50% tripunicine (cLncLncLn) (46.99%). In addition, this oil contains triglycerides in which punicic (cLn) acid is combined with palmitic (P), stearic (S), and linoleic (L) acid. The triglyceride dilinolein‐olein (LLO) was found in all samples analyzed. In particular, the highest values in the range of 14%–17% were encountered for strawberry, rosehip, raspberry, plum (all belonging to the Rosaceae family), and blackcurrant oils. However, statistical evaluation showed LLO is not significant (*p* = 0.445) for blackcurrant oil. On the contrary, the lowest value was observed in carrot oil (5.64%), pomegranate oil (7.90%), and radish (6.20%). Furthermore, radish oil is the only one in which stearin‐olein‐linolenin (SOLn) was found (0.37%). Linolein‐diolein (OOL) was found in all oils, with the highest value in plum seed oil corresponding to 23.65% and the lowest value in pomegranate seed oil (3.03%). This triglyceride was found significant for all the oil except carrot (*p* = 0.672) and raspberry (*p* = 0.078). Carrot and plum seed oils had a simpler profile than the other oils analyzed; however, they were the only samples in which stearin‐dilinolein (SLL) was detected (4.91% and 4.83%, *p* < 0.001). Palmitolein‐dilinolein (PoLL) was detected only in raspberry (0.96%), strawberry (0.35%), and blackcurrant (1.99%) seed oils. Finally, radish is the only seed oil in which triglycerides containing erucic acid are present combined with palmitic, oleic, gondoic (G), and stearic acid.

### 3.4 Volatile profile

**FIGURE 3:**
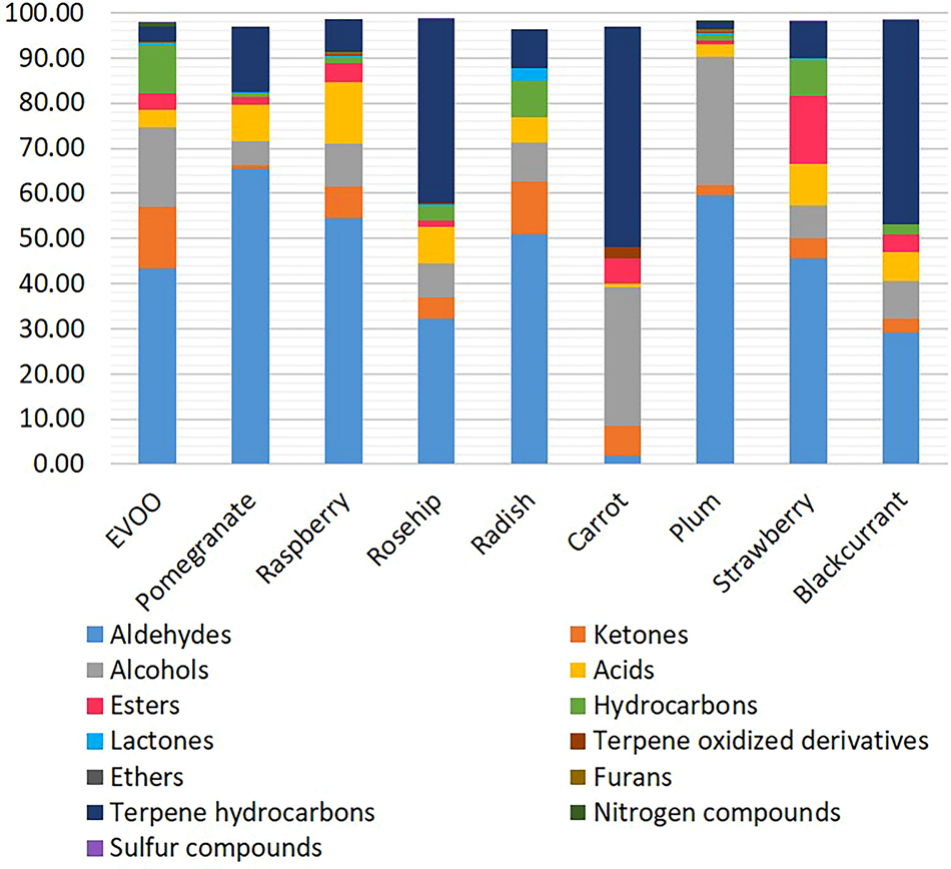
Classification of the main chemical groups present in the analyzed oil.

Overall, 346 compounds were identified through the analysis of the volatile profile of seed oils (Table S2), and it is well known that some of these have biological activity (Babukumar et al., 2017; Bahi et al., 2014; Baldissera et al., 2017; Guzmán‐Gutiérrez et al., 2015; Kamaraj et al., 2017; Rufino et al., 2015; Zhang et al., 2015). Volatile profile of the analyzed oils is shown in Figures S17–S24, and chemical families characterizing their volatile profile are visible in Figure 3. Aldehydes, responsible for the fruity and herbaceous notes, are the predominant class in pomegranate, plum, radish, strawberry, and raspberry seed oils. The most abundant compound belonging to this class are as follows: (2*E*,4*Z*)‐nonadienal (29.16%) in pomegranate, benzaldehyde (54.43%) in plum, hexanal (6.93%) and (2*E*,4*E*)‐heptadienal (5.43%) in radish, hexanal (10.30%) in strawberry, and raspberry (12.96%) seed oils.

In rosehip, carrot, and blackcurrant seed oils, the most representative class is terpene hydrocarbons, with α‐pinene (26.31%) in rosehip, carotol (27.43%) and α‐pinene (22.52%) in carrot, and α‐pinene (11.25%) and δ‐3‐carene (8.47%) in raspberry.

From the olfactory point of view, the seed oils analyzed presented compounds with characteristics similar to those of the fruits or vegetables from which they derive. Among the various classes of compounds identified, aldehydes, mainly derived from the degradation of FAs, significantly impact the flavor of edible oil products, since they are characterized by a very low odor threshold. Pomegranate and blackcurrant presented the highest content of pentanal (14.00% and 2.17%) with a fruity flavor. Hexanal, associated with a green and leafy aroma, is the major decomposition product of linoleic acid. It was found in raspberry (12.96%), strawberry (10.30%), blackcurrant (7.03%), radish (6.93%), and rosehip (5.88%) seed oil. Oils characterized by a high linoleic and α‐linolenic acid content also presented a good percentage of short‐chain trans aldehydes. (*E*)‐2‐butenal (5.13%) and (*E*)‐2‐pentenal (2.18%) were found mainly in radish; (*E*)‐2‐hexenal was detected principally in raspberry (1.82%) and strawberry (0.66%) seed oil. Relative high percentage of (*E*)‐2‐heptenal was detected in raspberry, rosehip, pomegranate, radish, blackcurrant, and strawberry in the range of 0.86%–3.83%. Raspberry and strawberry showed the higher content of (*E*)‐2‐octenal (2.02% and 3.15%, respectively). (*E*)‐2‐Nonenal was encountered in strawberry (1.28%).

Plum seed oil presents a high amount of benzaldehyde (54.43%), also present in strawberry, pomegranate, raspberry, and rosehip in a percentage >0.50%, which gives them a fruity aroma.

Alcohols, derived from lipids oxidation or reduction of carbonyl groups, had a higher threshold than aldehydes and had a sweet and pungent spice odor. The most abundant alcohols detected were *n*‐pentanol (1.80% in pomegranate and 0.53% in raspberry) and *n*‐hexanol (1.92% in rosehip, 0.88% in radish, 0.85% in blackcurrant, and 0.65% in raspberry seed oil), which are commonly found in volatile compounds of edible oil. In raspberry and plum seed oil, 2,3‐butadienol was also detected, which possesses a fruity and buttery nuance with a value of 1.96% and 0.59%, respectively.

Ketones, resulting from the oxidation of UFAs, contribute less to the flavor due to their odor threshold being higher than aldehydes and alcohols. The most relevant ketones detected were (3*E*,5*E*)‐3,5‐octadien‐2‐one with a fruity and green flavor (3.49% in raspberry, 2.36% in radish, and 2.14% in strawberry seed oil) and 1‐octen‐3‐one (1.24% in rosehip and 0.82% in blackcurrant seed oil) with herbal and earthy nuances.

Methyl and ethyl esters are the most influencing contributors to flavors, impacting fruity and floral aroma. Ethyl acetate having ethereal and fruity flavor was encountered in strawberry, blackcurrant, and raspberry with a percentage of 5.32%, 1.78%, and 1.76%, respectively. Ethyl hexanoate was detected in strawberry (2.24%).

Acids generally give a pungent aroma, which is not always desirable. The most relevant acids encountered were formic acid, found in raspberry, strawberry, rosehip, and blackcurrant seed oils in the range of 0.84%–2.71%, and acetic acid, detected in all the seed oils analyzed in the range of 0.32%–13.42%.

Other chemical classes that affect aroma profile are terpene hydrocarbons and their oxygenated derivatives. These molecules have very different threshold values and contribute to the aroma, giving woody, spicy, and citrus notes. Among them, α‐pinene and limonene were detected in all the seed oils in the range 0.53%–26.31% and 0.36%–3.34%, respectively.

Such a comprehensive investigation of the volatilome of these unconventional seed oils paves the way for the sensory evaluation by expert flavorist to correlate the olfactive notes with the identified compounds.

### 3.5 Principal component analysis of lipids

**FIGURE 4:**
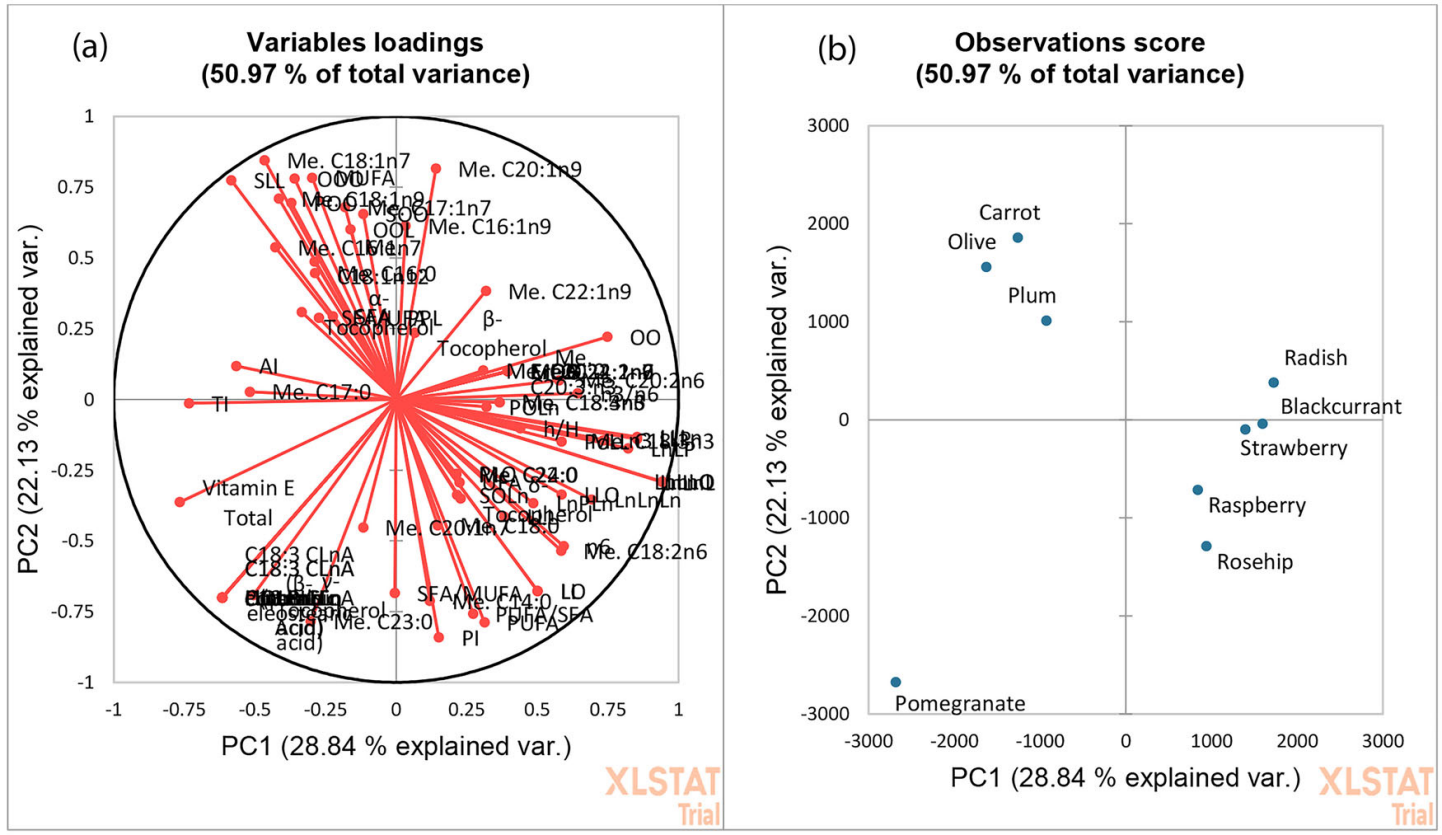
(a) Loading plot and score plot (b) based on lipidomic quality features of the analyzed oils.

Despite some differences in lipid composition that were easily visualized through the chromatographic comparison, PCA was performed to efficiently summarize such data and rapidly identify discriminant compounds. The first two principal components (PCs) explained 50.97% of total data variance. Each variable in Figure 4a is represented by the vectors, the direction and length of which indicate to what extent the variables in question affect the principal components. Figure 4b shows the score plot in the plane of the principal components, which illustrates the similarity between the lipidomic profiles of the oils analyzed. As reference sample, quantitative results previously obtained on an extra‐virgin olive oil were included in the PCA analysis.

**FIGURE 5:**
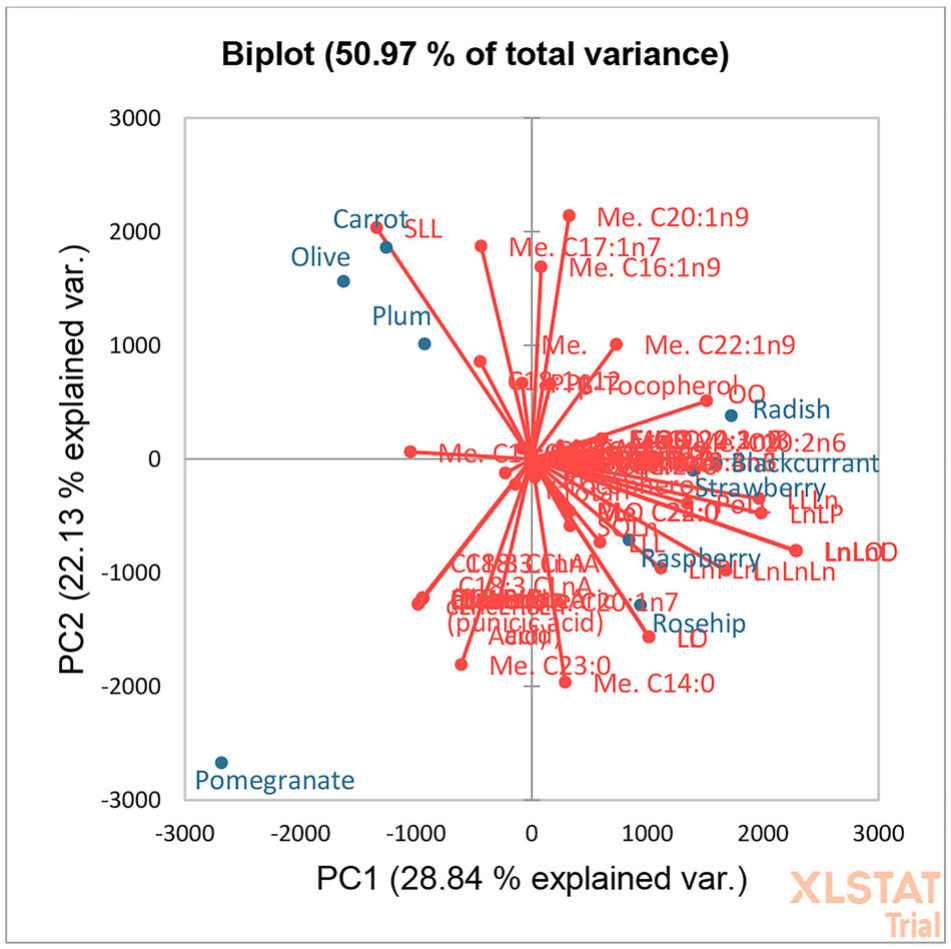
Biplots for principal component (PC1) and PC2 showing correlation between variables and observation for the lipidomic profile of the analyzed oils with a 95% confidence level.

The correlation between the original variables and the obtained principal components is shown in Figure 5.

The use of statistical methods in the evaluation of the lipids distribution showed that the combination of FAs, triglycerides, ω3/ω6 ratio, nutritional index, and tocopherols is useful to distinguish oils at the 95% confidence interval within the experimental space studied. PCA demonstrated that there is a significant difference in pomegranate oil distribution (located in the lower left region).

As visible in Table S1 that explains correlation between variable and factors, FAs and TAGs containing punicic acid, and γ‐tocopherol were the parameter that mainly contribute among the variables on PC1 for the differentiation of pomegranate seed oil. At more positive values on PC1 are grouped raspberry, rosehip, strawberry, blackcurrant, and radish seeds oils. For these samples, PC2 better describes the relationship between the variables and the samples. In this respect, triglycerides containing linoleic and linolenic acids, PUFA content, and PUFA/SFA ratio mainly affect raspberry, rosehip, and strawberry oils. On the contrary, the main variables that contribute to radish oil distribution are the eicosenoic (C20:1n9) and erucic (C22:1n9) acids. For blackcurrant seed oil, γ‐linolenic (C18:3n6) and stearidonic (C18:4n3) acids are the most influent variables, in accordance with GC results, which have revealed the presence of these FAs only in this oil. In the upper left region are grouped carrot, plum, and olive oil, which are common to have the highest content of oleic acid. For these oils, the components that most affect PCA distribution are (*Z*)‐hepatadecenoic acid (C17:1n7), oleic acid, MUFA, and triglycerides containing oleic and linoleic acid.

## 4 CONCLUSION

GC and LC technique were applied to deeply characterize novel alternative edible oil assets. The content of vitamin E, FAs, triglycerides, and volatile compounds in eight unconventional cold‐pressed seed oils was investigated and characterized in detail. The analyzed samples presented a high content of UFAs in the range of 80%–90%. Isomers of α‐linolenic acid, such as γ‐linolenic and punicic acid (related to possible antioxidant, anti‐cancer, anti‐obesity, and anti‐inflammatory activity), were detected in significant amounts in blackcurrant and pomegranate seed oils. Statistical analysis showed that all the oils were well distributed in 4 different regions. Similarities were revealed between olive, carrot, and plum seed oils, as well as between raspberry, rosehip, strawberry, blackcurrant, and radish seed oils. The first three have in common a high oleic acid content, a conspicuous presence of triglycerides containing oleic and linoleic acid, and a high value of MUFAs. Raspberry, rosehip, strawberry, blackcurrant, and radish oils are characterized by a high amount of linolenic acid and its triglycerides. Furthermore, volatile profile analysis revealed for all the samples the presence of a conspicuous number of compounds belonging to terpene hydrocarbons and their oxygenated derivatives, such as limonene, α‐pinene, β‐pinene, β‐caryophyllene, and linalool. In addition, compounds commonly detected in the corresponding fruit and vegetables, and commonly related with their typical flavor were also revealed.

Nutritional values were evaluated, and very beneficial SFA/UFA were found for all seed oils.

Moreover, hypocholesterolemic/hypercholesterolemic, AI, and TI indices were extremely favorable in each investigated sample, even compared to other common vegetable oils. In addition, the analyzed oils are a natural source of antioxidant molecules such as vitamin E. Given the growing interest in nutraceuticals, they could find applications in the formulation of foods with specific health effects for prevention of chronic and degenerative diseases, as well as be used in the cosmetics and pharmaceuticals industry, even related to their taste and olfactive notes.

## AUTHOR CONTRIBUTIONS

**Francesca Rigano**: Conceptualization; visualization; writing—original draft. **Federica Vento**: Data curation; investigation. **Cinzia Cafarella**: Data curation; investigation. **Emanuela Trovato**: Writing—original draft; writing—review and editing; conceptualization. **Alessandra Trozzi**: Conceptualization; visualization. **Paola Dugo**: Supervision; writing—original draft. **Luigi Mondello**: Supervision; project administration.

## CONFLICT OF INTEREST STATEMENT

The authors declare no conflicts of interest.

## Supporting information




**Figure S1**. Chromatogram of fatty acid profile of raspberry seed oil acquired by GC‐FID analysis.
**Figure S2**. Chromatogram of fatty acid profile of rosehip seed oil acquired by GC‐FID analysis.
**Figure S3**. Chromatogram of fatty acid profile of pomegranate seed oil acquired by GC‐FID analysis.
**Figure S4**. Chromatogram of fatty acid profile of radish seed oil acquired by GC‐FID analysis.
**Figure S5**. Chromatogram of fatty acid profile of carrot seed oil acquired by GC‐FID analysis.
**Figure S6**. Chromatogram of fatty acid profile of strawberry seed oil acquired by GC‐FID analysis.
**Figure S7**. Chromatogram of fatty acid profile of blackcurrant seed oil acquired by GC‐FID analysis.
**Figure S8** Chromatogram of the DAGs and TAGs identified in raspberry seed oil.
**Figure S9**. Chromatogram of the DAGs and TAGs identified in rosehip seed oil.
**Figure S10**. Chromatogram of the DAGs and TAGs identified in pomegranate seed oil.
**Figure S11**. Chromatogram of the DAGs and TAGs identified in radish seed oil.
**Figure S12**. Chromatogram of the DAGs and TAGs identified in carrot seed oil.
**Figure S13**. Chromatogram of the DAGs and TAGs identified in strawberry seed oil.
**Figure S14**. Chromatogram of the DAGs and TAGs identified in blackcurrant seed oil.
**Figure S15**. Similarity search results for LLL. Structure elucidation is also provided and linear retention index values are circled in blue.
**Figure S16**. Similarity search results for LLL. Structure elucidation is also provided and linear retention index values are circled in blue.
**Table S1**. PCA factor loading and percentage of variance explained for the lipidomic profile of the investigated seed oils.
**Figure S17**. Chromatogram of volatile compounds identified in raspberry seed oil.
**Figure S18**. Chromatogram of volatile compounds identified in rosehip seed oil.
**Figure S19**. Chromatogram of volatile compounds identified in pomegranate seed oil.
**Figure S20**. Chromatogram of volatile compounds identified in radish seed oil.
**Figure S21**. Chromatogram of volatile compounds identified in carrot seed oil.
**Figure S22**. Chromatogram of volatile compounds identified in plum seed oil.
**Figure S23**. Chromatogram of volatile compounds identified in strawberry seed oil.
**Figure S24**. Chromatogram of volatile compounds identified in blackcurrant seed oil.
**Table S2** Volatile compound identified in the fruit seed oils analysed along with experimental linear retention index (LRI exp.) and reference linear retention index (LRI ref.).

## Data Availability

Data will be made available on request.
