# Supplementary material for: Determination of main lipids and volatile compounds in unconventional cold‐pressed seed oils through chromatographic techniques
Source: J Food Sci. 2025 Jan 19;90(1):e17661. doi: 10.1111/1750-3841.17661 (PMC11743071; doi:10.1111/1750-3841.17661)
Supplement: Supplementary file 1 — Figure S1. Chromatogram of fatty acid profile of raspberry seed oil acquired by GC‐FID analysis. Figure S2. Chromatogram of fatty acid profile of rosehip seed oil acquired by GC‐FID analysis. Figure S3. Chromatogram of fatty acid profile of pomegranate seed oil acquired by GC‐FID analysis. Figure S4. Chromatogram of fatty acid profile of radish seed oil acquired by GC‐FID analysis. Figure S5. Chromatogram of fatty acid profile of carrot seed oil acquired by GC‐FID analysis. Figure S6. Chromatogram of fatty acid profile of strawberry seed oil acquired by GC‐FID analysis. Figure S7. Chromatogram of fatty acid profile of blackcurrant seed oil acquired by GC‐FID analysis. Figure S8 Chromatogram of the DAGs and TAGs identified in raspberry seed oil. Figure S9. Chromatogram of the DAGs and TAGs identified in rosehip seed oil. Figure S10. Chromatogram of the DAGs and TAGs identified in pomegranate seed oil. Figure S11. Chromatogram of the DAGs and TAGs identified in radish seed oil. Figure S12. Chromatogram of the DAGs and TAGs identified in carrot seed oil. Figure S13. Chromatogram of the DAGs and TAGs identified in strawberry seed oil. Figure S14. Chromatogram of the DAGs and TAGs identified in blackcurrant seed oil. Figure S15. Similarity search results for LLL. Structure elucidation is also provided and linear retention index values are circled in blue. Figure S16. Similarity search results for LLL. Structure elucidation is also provided and linear retention index values are circled in blue. Table S1. PCA factor loading and percentage of variance explained for the lipidomic profile of the investigated seed oils. Figure S17. Chromatogram of volatile compounds identified in raspberry seed oil. Figure S18. Chromatogram of volatile compounds identified in rosehip seed oil. Figure S19. Chromatogram of volatile compounds identified in pomegranate seed oil. Figure S20. Chromatogram of volatile compounds identified in radish seed oil. Figure S21. Chromatogram of volatile compo [file JFDS-90-0-s001.docx]

**Supplementary material**

**Determination of main lipids and volatile compounds in unconventional cold-pressed seed oils through chromatographic techniques**

Francesca Rigano^1^, Federica Vento^1^, Cinzia Cafarella^1^, Emanuela Trovato^1,^*, Alessandra Trozzi^1^, Paola Dugo^1,2^, Luigi Mondello^1,2^

*corresponding author: ematrovato@unime.it

^1^Messina Institute of Technology c/o Department of Chemical, Biological, Pharmaceutical and Environmental Sciences, former Veterinary School, University of Messina, Messina, Italy

^2^Chromaleont s.r.l., c/o Department of Chemical, Biological, Pharmaceutical and Environmental Sciences, former Veterinary School, University of Messina, Messina, Italy

**Figure S1.** Chromatogram of fatty acid profile of raspberry seed oil acquired by GC-FID analysis.

**Figure S2.** Chromatogram of fatty acid profile of rosehip seed oil acquired by GC-FID analysis.

**Figure S3.** Chromatogram of fatty acid profile of pomegranate seed oil acquired by GC-FID analysis.

**Figure S4.** Chromatogram of fatty acid profile of radish seed oil acquired by GC-FID analysis.

**Figure S5.** Chromatogram of fatty acid profile of carrot seed oil acquired by GC-FID analysis.

**Figure S6.** Chromatogram of fatty acid profile of strawberry seed oil acquired by GC-FID analysis.

**Figure S7.** Chromatogram of fatty acid profile of blackcurrant seed oil acquired by GC-FID analysis.

**Figure S8** Chromatogram of the DAGs and TAGs identified in raspberry seed oil.

**Figure S9.** Chromatogram of the DAGs and TAGs identified in rosehip seed oil.

**Figure S10.** Chromatogram of the DAGs and TAGs identified in pomegranate seed oil.

**Figure S11.** Chromatogram of the DAGs and TAGs identified in radish seed oil.

**Figure S12.** Chromatogram of the DAGs and TAGs identified in carrot seed oil.

**Figure S13.** Chromatogram of the DAGs and TAGs identified in strawberry seed oil.

**Figure S14.** Chromatogram of the DAGs and TAGs identified in blackcurrant seed oil.

**Figure S15.** Similarity search results for LLL. Structure elucidation is also provided and linear retention index values are circled in blue.

**Figure S16.** Similarity search results for LLL. Structure elucidation is also provided and linear retention index values are circled in blue.

**Table S1**. PCA factor loading and percentage of variance explained for the lipidomic profile of the investigated seed oils.

**Figure S17.** Chromatogram of volatile compounds identified in raspberry seed oil.

**Figure S18.** Chromatogram of volatile compounds identified in rosehip seed oil.

**Figure S19.** Chromatogram of volatile compounds identified in pomegranate seed oil.

**Figure S20.** Chromatogram of volatile compounds identified in radish seed oil.

**Figure S21.** Chromatogram of volatile compounds identified in carrot seed oil.

**Figure S22.** Chromatogram of volatile compounds identified in plum seed oil.

**Figure S23.** Chromatogram of volatile compounds identified in strawberry seed oil.

**Figure S24.** Chromatogram of volatile compounds identified in blackcurrant seed oil.

**Table S2** Volatile compound identified in the fruit seed oils analysed along with experimental linear retention index (LRI exp.) and reference linear retention index (LRI ref.).


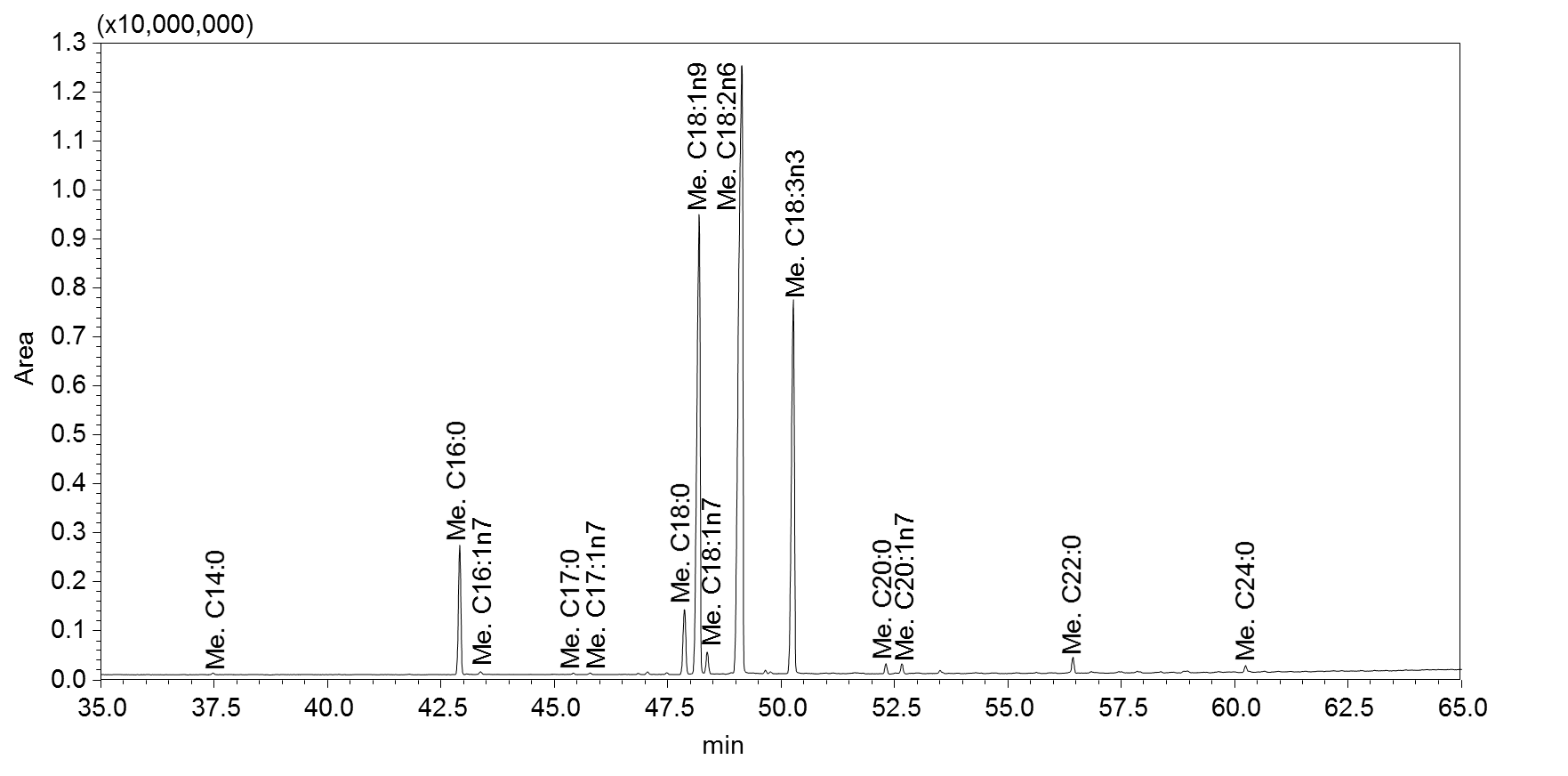


**Figure S1.** Chromatogram of fatty acid profile of raspberry seed oil acquired by GC-FID analysis.


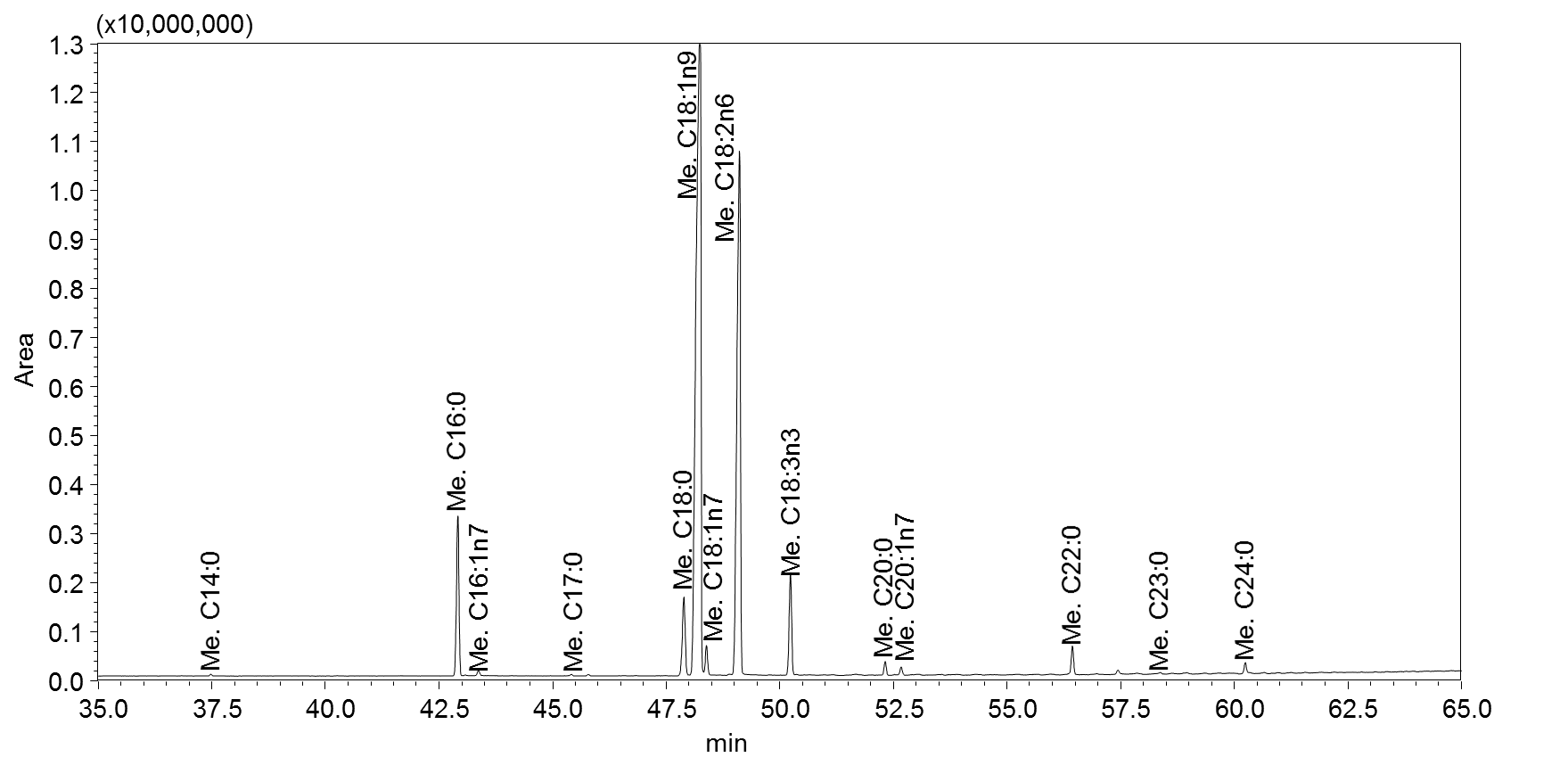


**Figure S2.** Chromatogram of fatty acid profile of rosehip seed oil acquired by GC-FID analysis.


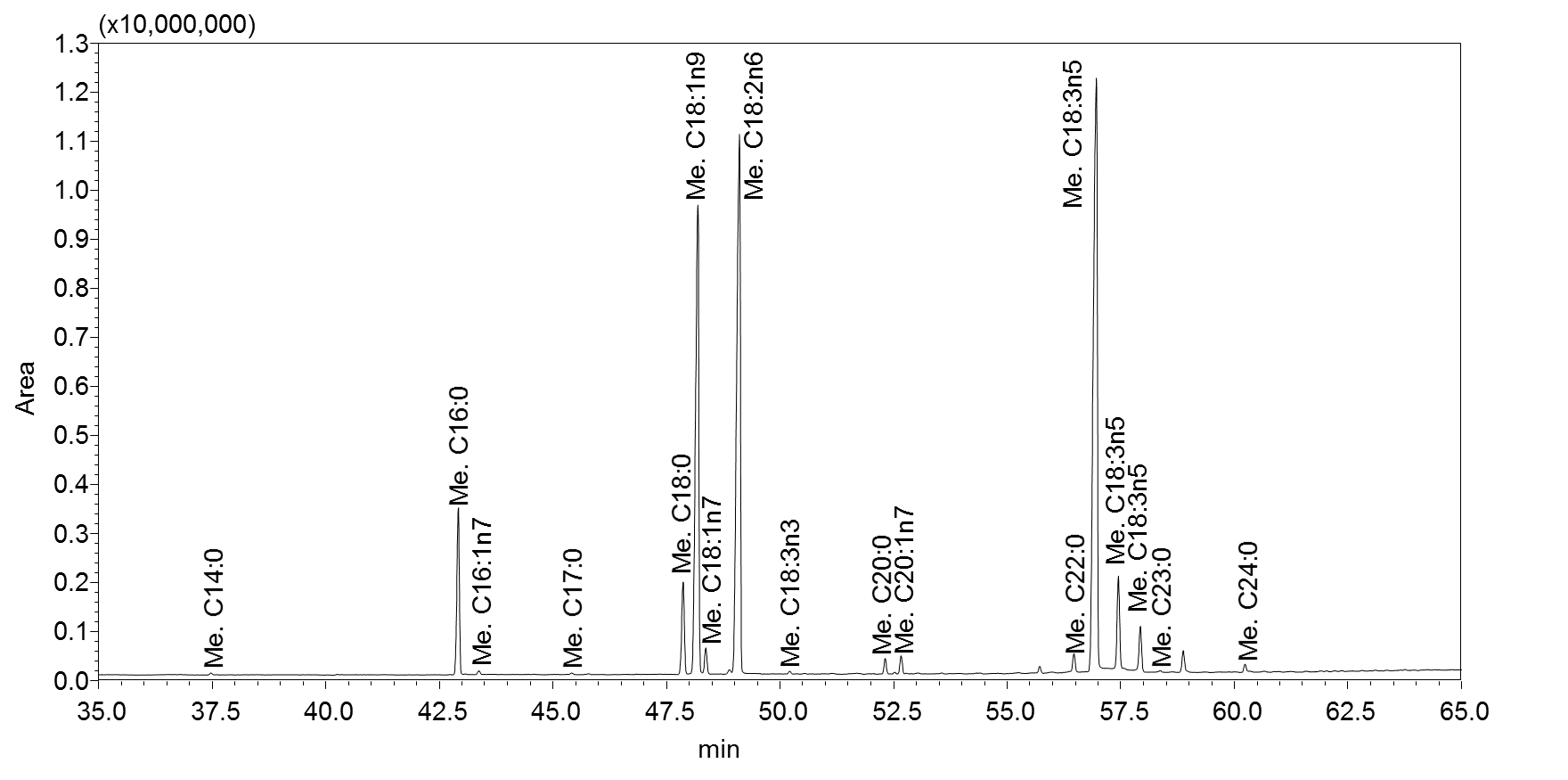


**Figure S3.** Chromatogram of fatty acid profile of pomegranate seed oil acquired by GC-FID analysis.


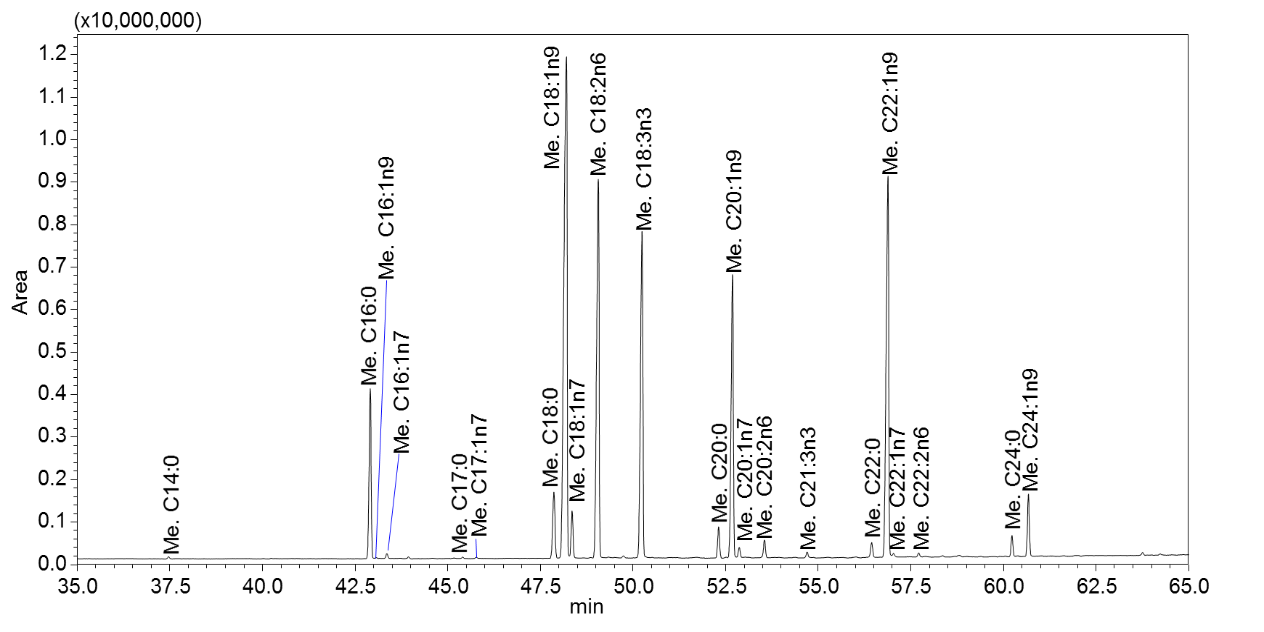


**Figure S4.** Chromatogram of fatty acid profile of radish seed oil acquired by GC-FID analysis.


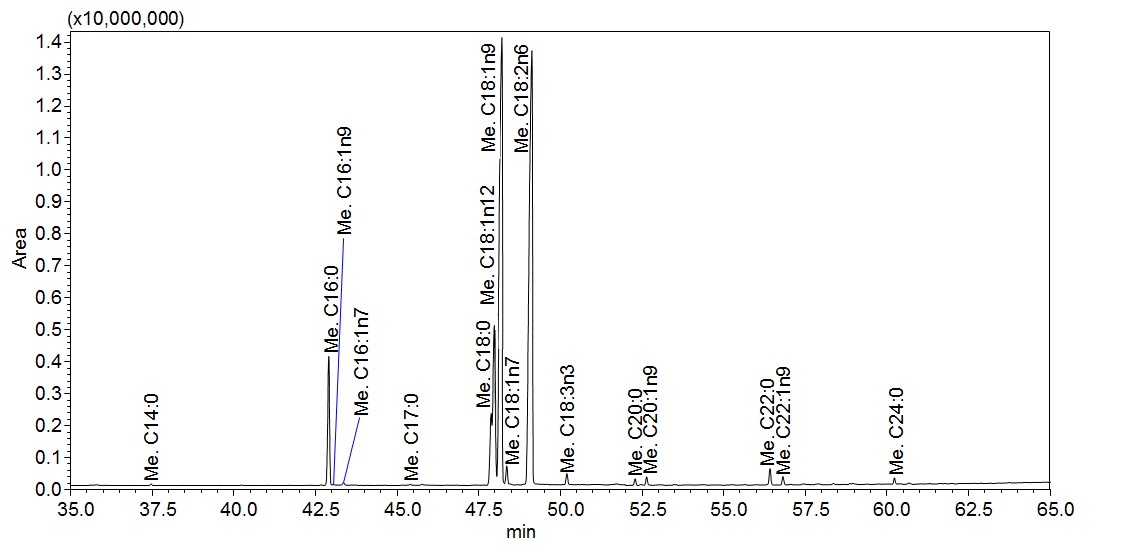


**Figure S5.** Chromatogram of fatty acid profile of carrot seed oil acquired by GC-FID analysis.


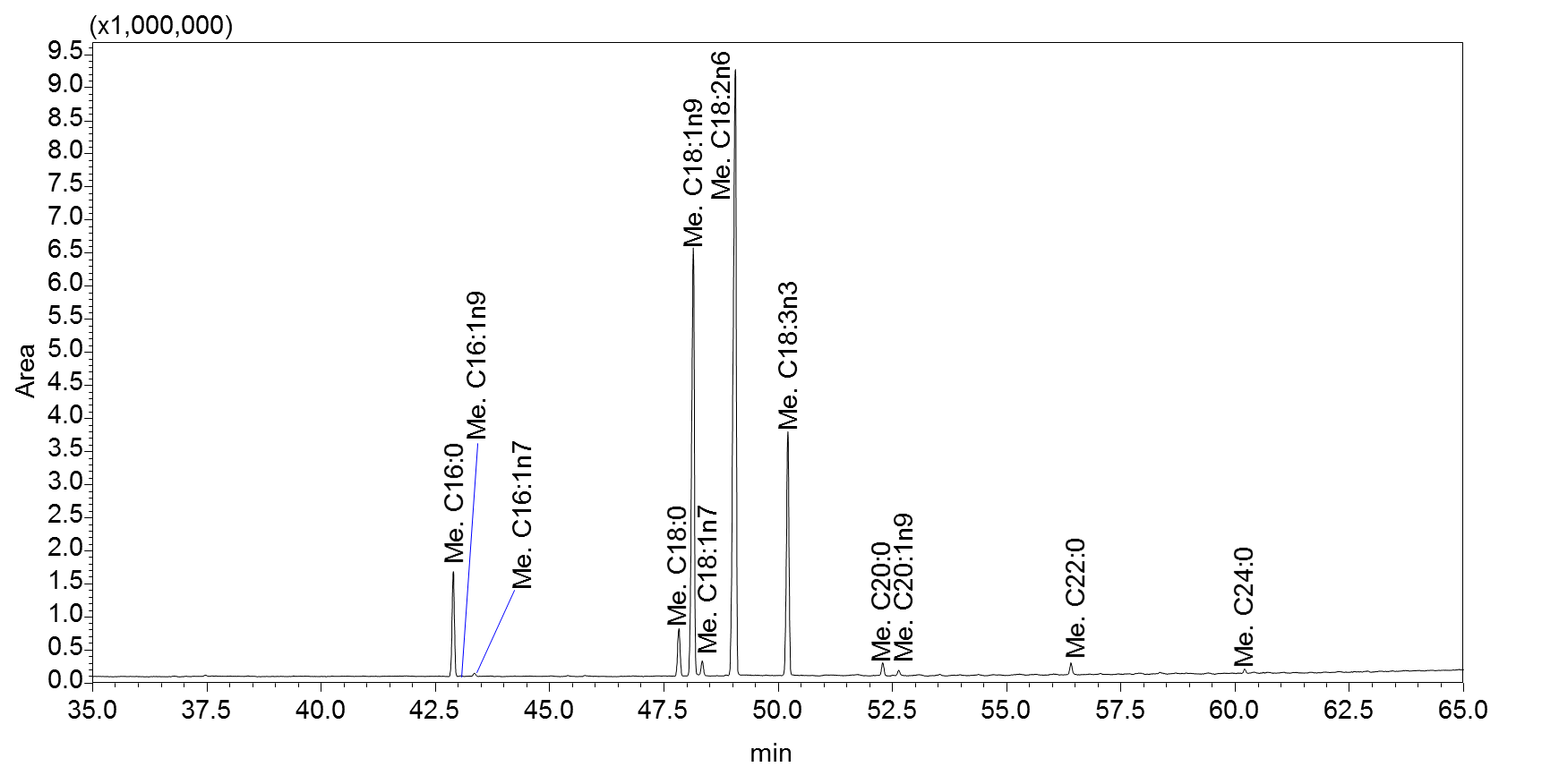


**Figure S6.** Chromatogram of fatty acid profile of strawberry seed oil acquired by GC-FID analysis.


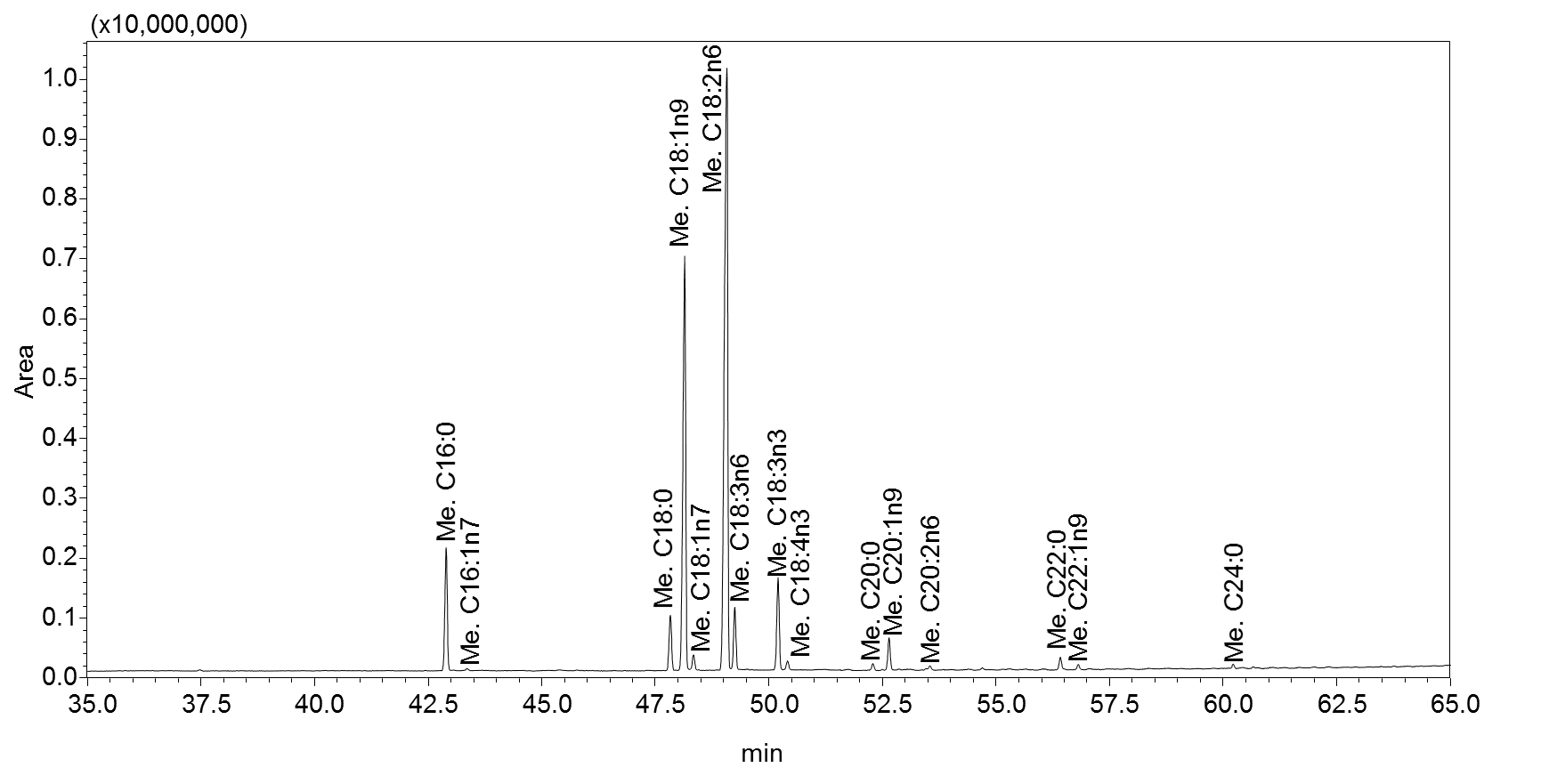


**Figure S7.** Chromatogram of fatty acid profile of blackcurrant seed oil acquired by GC-FID analysis.


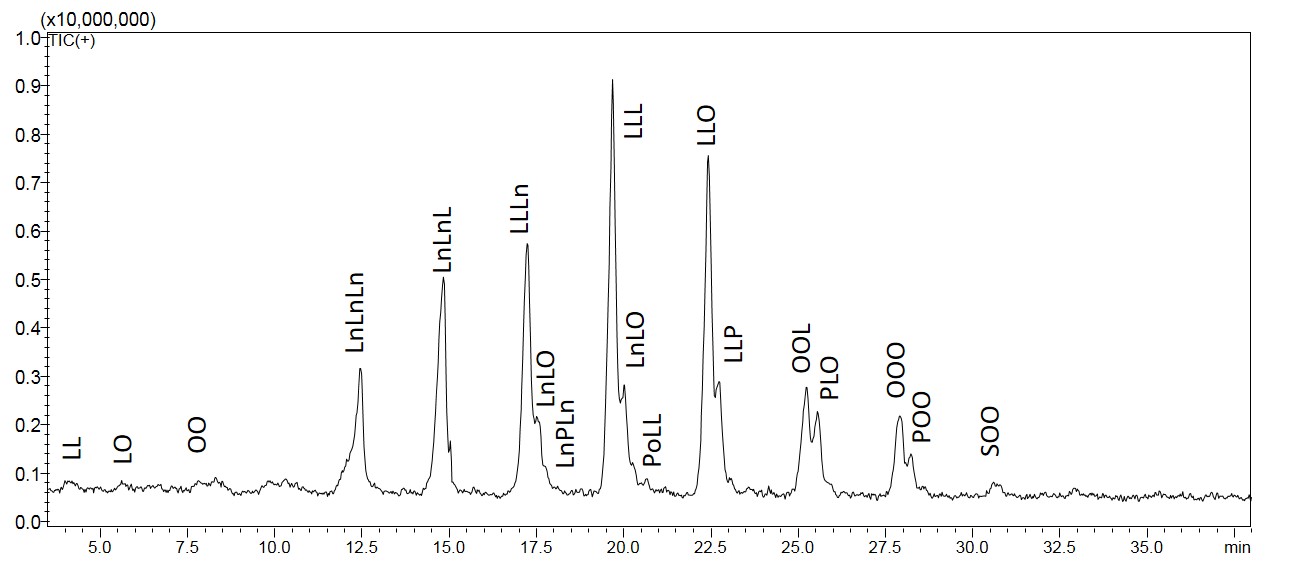
**Figure S8.** Chromatogram of the DAGs and TAGs identified in raspberry seed oil.


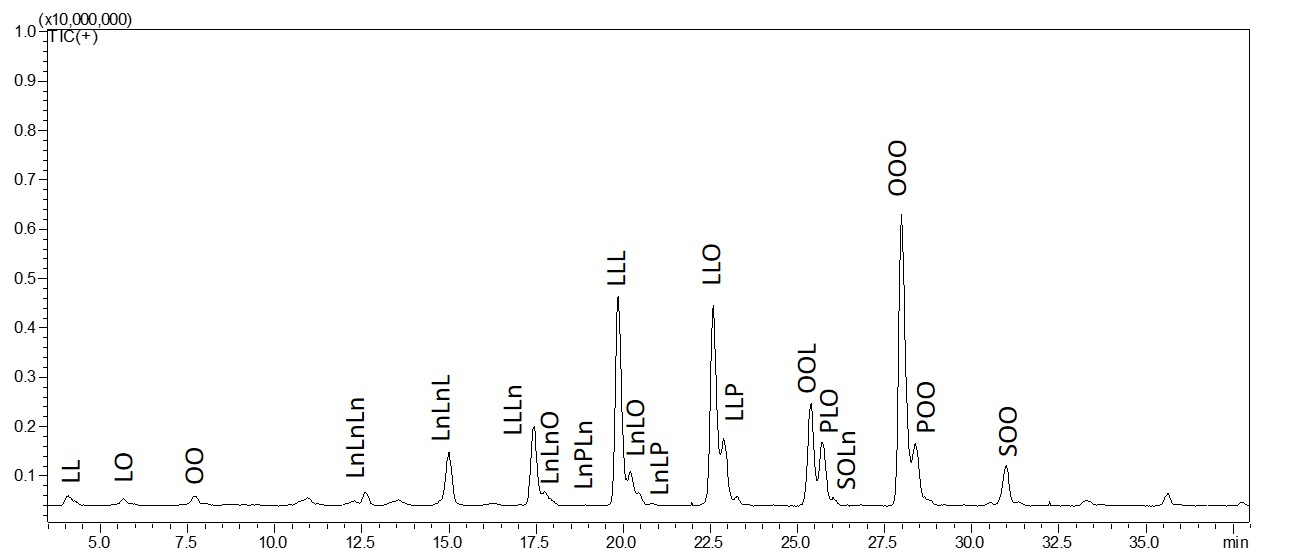
**Figure S9.** Chromatogram of the DAGs and TAGs identified in rosehip seed oil.
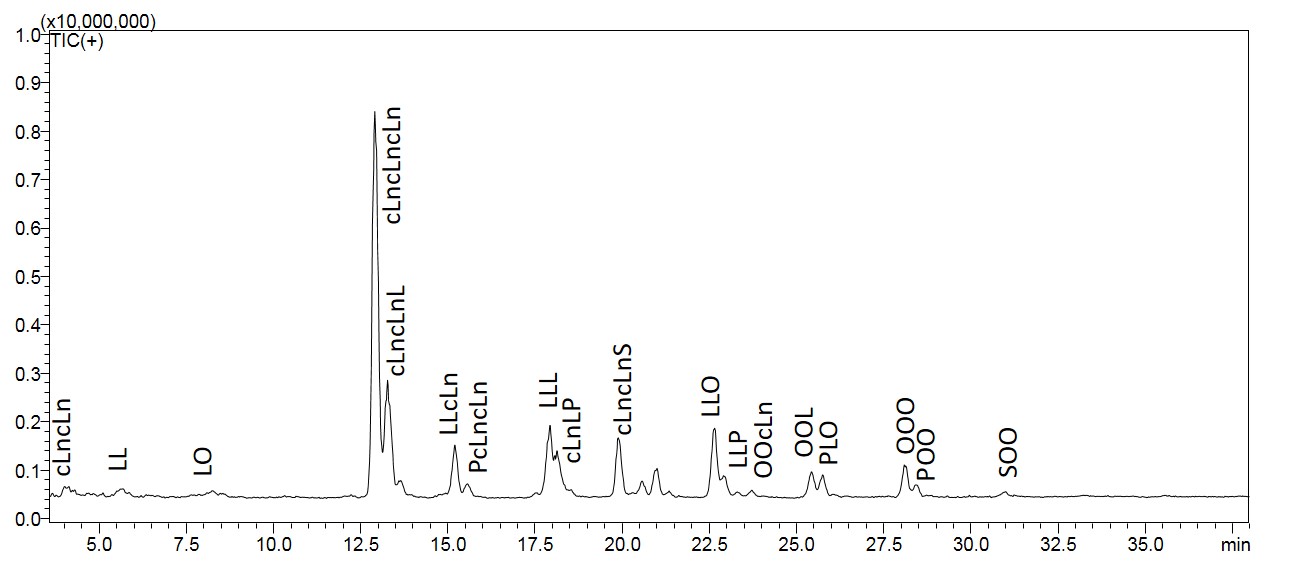
**Figure S10.** Chromatogram of the DAGs and TAGs identified in pomegranate seed oil.


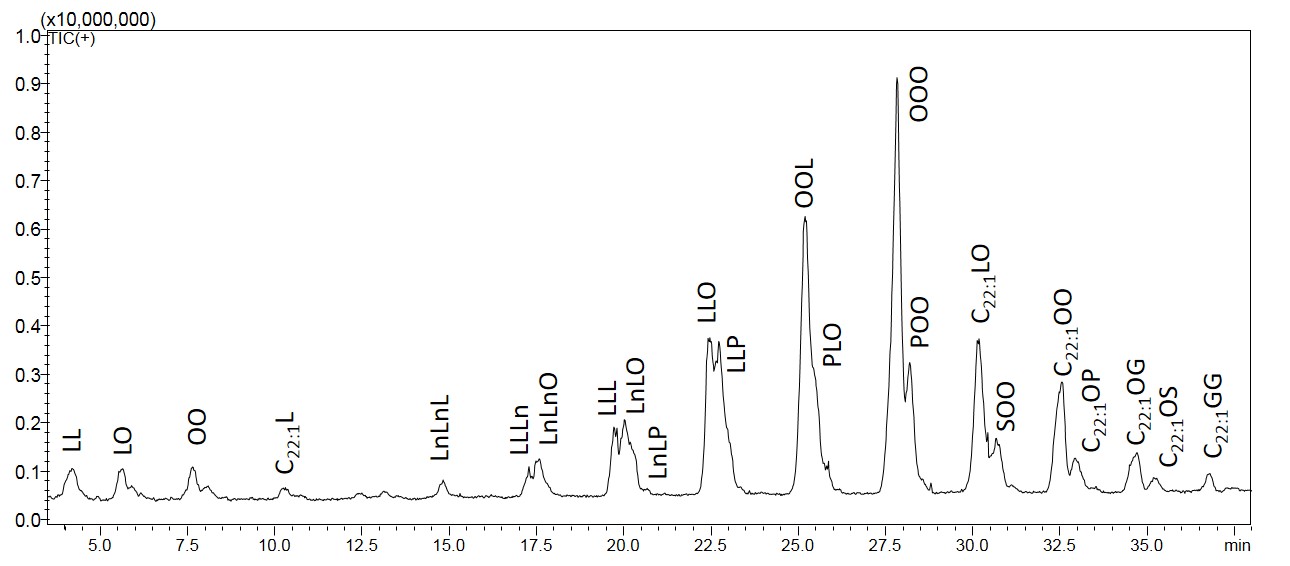


**Figure S11.** Chromatogram of the DAGs and TAGs identified in radish seed oil.


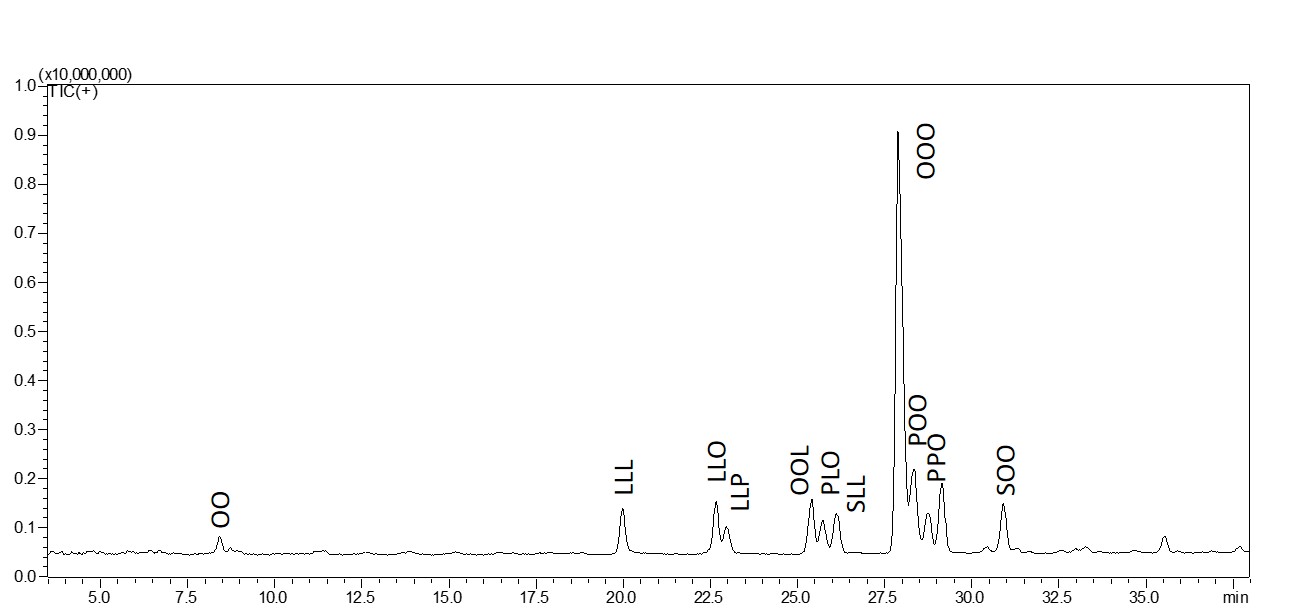


**Figure S12.** Chromatogram of the DAGs and TAGs identified in carrot seed oil.


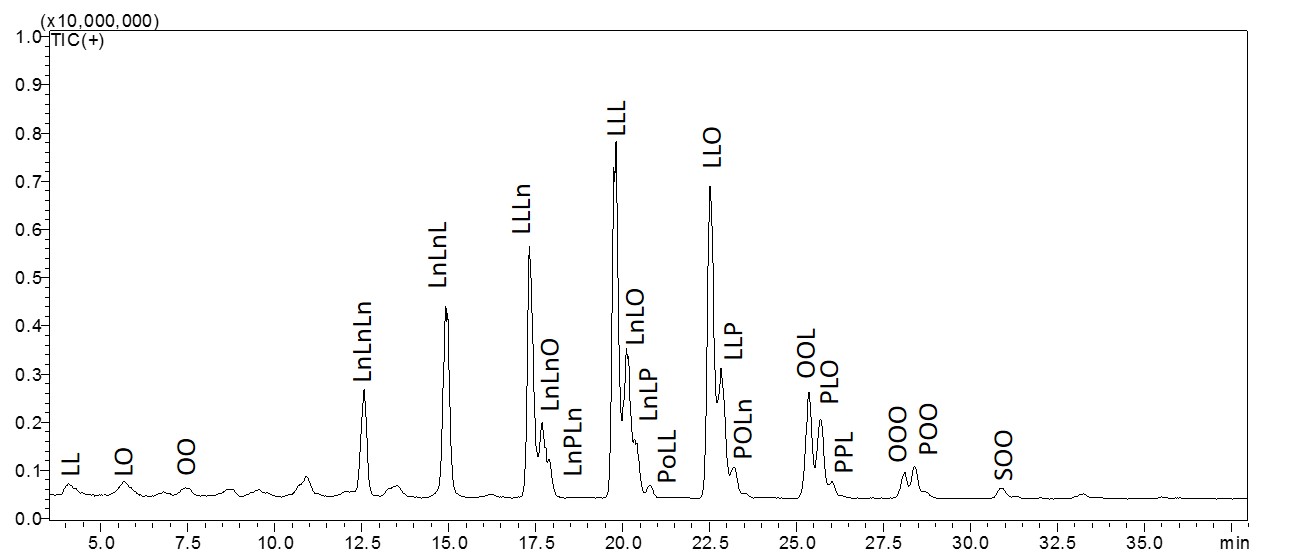


**Figure S13.** Chromatogram of the DAGs and TAGs identified in strawberry seed oil.


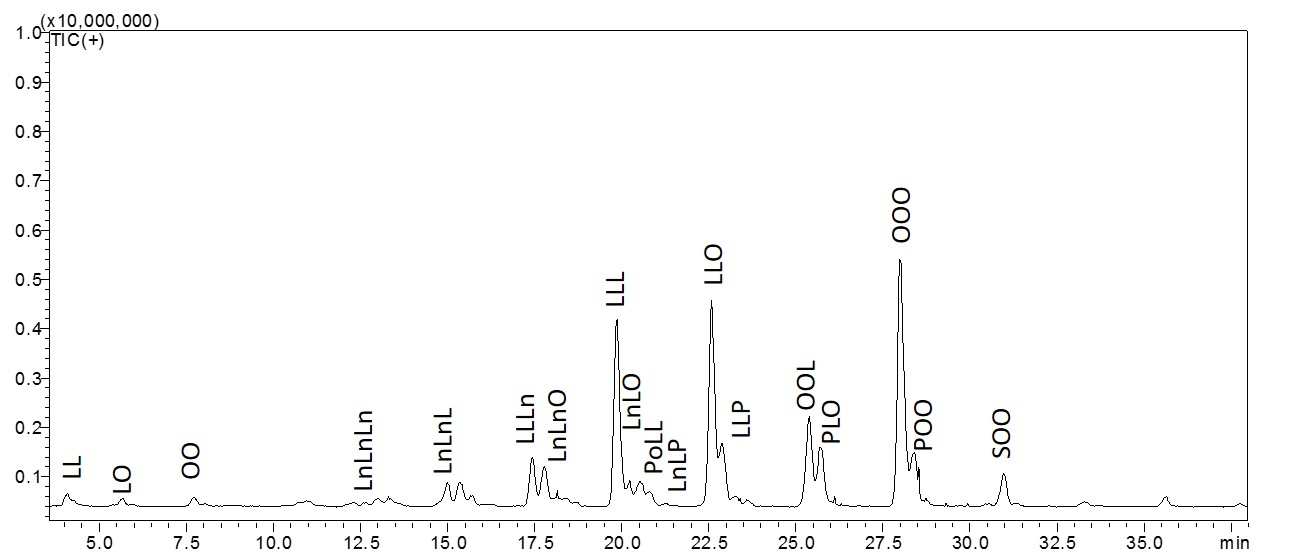


**Figure S14.** Chromatogram of the DAGs and TAGs identified in blackcurrant seed oil.

**Figure S15.** Similarity search results for LLL. Structure elucidation is also provided and linear retention index values are circled in blue.

**Figure S16.** Similarity search results for LLP. Structure elucidation is also provided for both LLL and LLP and linear retention index values are circled in blue.

**Table S1**. PCA factor loading and percentage of variance explained for the lipidomic profile of the investigated seed oils.

|  | PC1 | PC2 | PC3 | PC4 | PC5 |
| --- | --- | --- | --- | --- | --- |
| Me. C14:0 | 0.120 | -0.712 | -0.462 | 0.483 | -0.077 |
| Me. C16:0 | -0.288 | 0.446 | 0.057 | 0.174 | 0.308 |
| Me. C16:1n9 | 0.034 | 0.613 | -0.321 | 0.027 | 0.509 |
| Me. C16:1n7 | -0.428 | 0.538 | 0.089 | 0.012 | -0.491 |
| Me. C17:0 | -0.519 | 0.027 | -0.434 | 0.649 | -0.303 |
| Me. C17:1n7 | -0.181 | 0.679 | -0.349 | 0.376 | -0.275 |
| Me. C18:0 | 0.147 | -0.445 | 0.140 | 0.158 | 0.531 |
| Me. C18:1n12 | -0.289 | 0.487 | 0.035 | 0.020 | 0.212 |
| Me. C18:1n9 | -0.416 | 0.710 | 0.122 | 0.271 | -0.244 |
| Me. C18:1n7 | -0.467 | 0.846 | -0.136 | 0.136 | 0.054 |
| Me. C18:2n6 | 0.585 | -0.535 | 0.481 | -0.217 | 0.055 |
| Me. C18:3n6 | 0.366 | -0.010 | 0.253 | -0.571 | 0.211 |
| Me. C18:3n3 | 0.733 | -0.145 | -0.124 | 0.176 | 0.046 |
| Me. C18:4n3 | 0.366 | -0.010 | 0.253 | -0.571 | 0.211 |
| Me. C20:0 | 0.309 | 0.102 | -0.659 | 0.092 | 0.520 |
| Me. C20:1n9 | 0.140 | 0.816 | -0.125 | -0.501 | 0.082 |
| Me. C20:1n7 | -0.117 | -0.453 | -0.433 | 0.671 | 0.177 |
| Me. C20:2n6 | 0.576 | 0.068 | -0.497 | -0.423 | 0.161 |
| Me. C20:3:n3 | 0.396 | 0.100 | -0.910 | 0.012 | 0.003 |
| Me. C22:0 | 0.212 | -0.265 | -0.075 | 0.224 | 0.892 |
| Me. C22:1n9 | 0.318 | 0.384 | -0.422 | -0.357 | 0.282 |
| C18:3 CLnA (punicic acid) | -0.616 | -0.700 | -0.209 | -0.258 | 0.114 |
| Me. C22:1n7 | 0.396 | 0.100 | -0.910 | 0.012 | 0.003 |
| C18:3 CLnA (catalpic Acid) | -0.616 | -0.700 | -0.209 | -0.258 | 0.114 |
| Me. C22:2n6 | 0.396 | 0.100 | -0.910 | 0.012 | 0.003 |
| C18:3 CLnA (β-eleostearic acid) | -0.616 | -0.700 | -0.209 | -0.258 | 0.114 |
| Me. C23:0 | -0.302 | -0.785 | -0.017 | 0.200 | 0.026 |
| C18:3 CLnA | -0.616 | -0.700 | -0.209 | -0.258 | 0.114 |
| Me. C24:0 | 0.213 | -0.265 | -0.076 | 0.224 | 0.892 |
| Me. C24:1n9 | 0.396 | 0.100 | -0.910 | 0.012 | 0.003 |
| SFA | -0.224 | 0.293 | 0.006 | 0.220 | 0.476 |
| MUFA | -0.297 | 0.784 | -0.202 | 0.248 | -0.189 |
| PUFA | 0.313 | -0.788 | 0.193 | -0.265 | 0.123 |
| UFA | 0.224 | -0.293 | -0.006 | -0.221 | -0.476 |
| SFA/MUFA | -0.004 | -0.684 | 0.228 | -0.316 | 0.399 |
| PUFA/SFA | 0.272 | -0.757 | 0.197 | -0.169 | 0.038 |
| SFA/UFA | -0.273 | 0.288 | 0.034 | 0.213 | 0.446 |
| n3 | 0.742 | -0.145 | -0.124 | 0.164 | 0.051 |
| n6 | 0.594 | -0.519 | 0.474 | -0.244 | 0.066 |
| n3/n6 | 0.643 | 0.021 | -0.666 | 0.149 | 0.068 |
| AI | -0.566 | 0.118 | -0.028 | 0.061 | 0.354 |
| TI | -0.734 | -0.013 | 0.012 | 0.016 | 0.294 |
| PI | 0.151 | -0.842 | 0.040 | -0.267 | 0.145 |
| h/H | 0.440 | -0.102 | 0.422 | 0.170 | -0.315 |
| α-Tocopherol | -0.335 | 0.309 | 0.238 | 0.489 | 0.447 |
| β-Tocopherol | 0.066 | 0.236 | 0.455 | 0.477 | 0.371 |
| γ-Tocopherol | -0.506 | -0.691 | -0.194 | -0.281 | -0.227 |
| δ-Tocopherol | 0.228 | -0.351 | 0.199 | 0.294 | -0.131 |
| Vitamin E Total | -0.766 | -0.363 | 0.093 | 0.256 | 0.197 |
| cLncLn | -0.616 | -0.700 | -0.209 | -0.258 | 0.114 |
| LL | 0.501 | -0.678 | -0.062 | -0.241 | -0.462 |
| LO | 0.501 | -0.677 | -0.062 | -0.241 | -0.463 |
| OO | 0.748 | 0.221 | 0.123 | -0.030 | -0.389 |
| ErL | 0.396 | 0.100 | -0.910 | 0.012 | 0.003 |
| cLncLncLn | -0.616 | -0.700 | -0.209 | -0.258 | 0.114 |
| LnLnLn | 0.692 | -0.354 | 0.610 | 0.097 | 0.103 |
| LnLnL | 0.942 | -0.292 | 0.037 | 0.106 | 0.104 |
| cLncLnL | -0.616 | -0.700 | -0.209 | -0.258 | 0.114 |
| LLLn | 0.854 | -0.133 | 0.090 | -0.037 | -0.481 |
| LnLnO | 0.943 | -0.291 | 0.034 | 0.102 | 0.105 |
| LLcLn | -0.616 | -0.700 | -0.209 | -0.258 | 0.114 |
| LnPLn | 0.486 | -0.367 | 0.474 | 0.482 | -0.032 |
| PcLncLn | -0.616 | -0.700 | -0.209 | -0.258 | 0.114 |
| LLL | 0.383 | -0.415 | -0.032 | -0.293 | -0.395 |
| LnLO | 0.943 | -0.291 | 0.035 | 0.103 | 0.105 |
| PoLL | 0.586 | -0.149 | 0.518 | -0.247 | 0.162 |
| cLnLP | -0.616 | -0.700 | -0.209 | -0.258 | 0.114 |
| LnLP | 0.821 | -0.173 | -0.094 | -0.205 | 0.205 |
| cLncLnS | -0.616 | -0.700 | -0.209 | -0.258 | 0.114 |
| LLO | 0.585 | -0.336 | 0.643 | -0.128 | -0.330 |
| LLP | 0.853 | -0.140 | -0.351 | -0.107 | 0.225 |
| POLn | 0.320 | -0.026 | 0.322 | -0.287 | 0.190 |
| OOcLn | -0.616 | -0.700 | -0.209 | -0.258 | 0.114 |
| OOL | -0.162 | 0.600 | -0.213 | 0.051 | -0.427 |
| PLO | 0.213 | -0.263 | -0.075 | 0.225 | 0.892 |
| SLL | -0.583 | 0.774 | 0.104 | 0.063 | -0.187 |
| PPL | -0.041 | 0.290 | 0.277 | 0.008 | 0.446 |
| SOLn | 0.216 | -0.337 | 0.186 | 0.522 | -0.080 |
| OOO | -0.360 | 0.781 | 0.008 | 0.104 | -0.077 |
| POO | -0.372 | 0.695 | -0.113 | 0.278 | 0.293 |
| ErLO | 0.396 | 0.100 | -0.910 | 0.012 | 0.003 |
| SOO | -0.116 | 0.655 | -0.005 | 0.179 | 0.275 |
| ErOO | 0.396 | 0.100 | -0.910 | 0.012 | 0.003 |
| ErOP | 0.396 | 0.100 | -0.910 | 0.012 | 0.003 |
| ErOG | 0.396 | 0.100 | -0.910 | 0.012 | 0.003 |
| ErOS | 0.396 | 0.100 | -0.910 | 0.012 | 0.003 |
| ErGG | 0.396 | 0.100 | -0.910 | 0.012 | 0.003 |
| *Variance explained in %* | *28.84* | *22.12* | *18.66* | *9.33* | *8.18* |

**
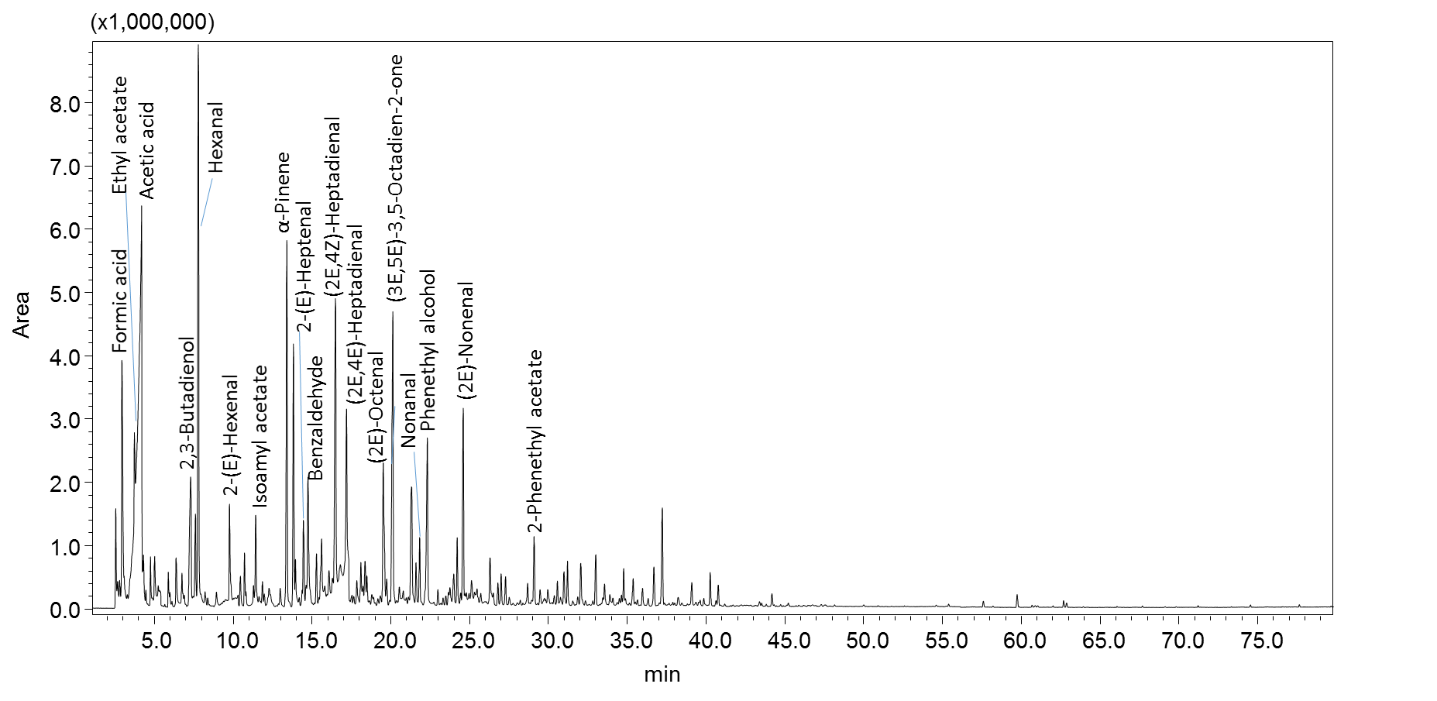
Figure S17.** Chromatogram of volatile compounds identified in raspberry seed oil.

**
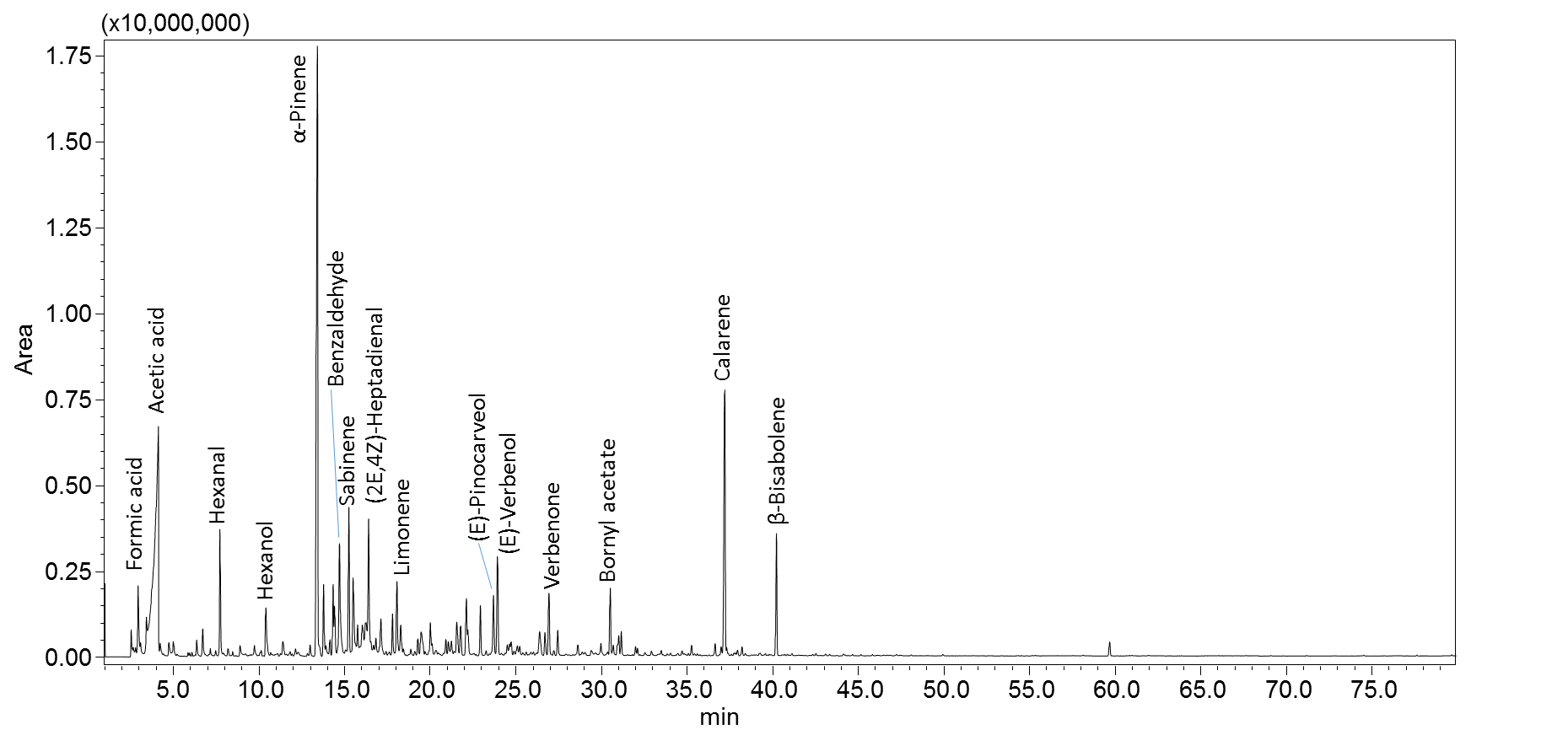
Figure S18.** Chromatogram of volatile compounds identified in rosehip seed oil.

**
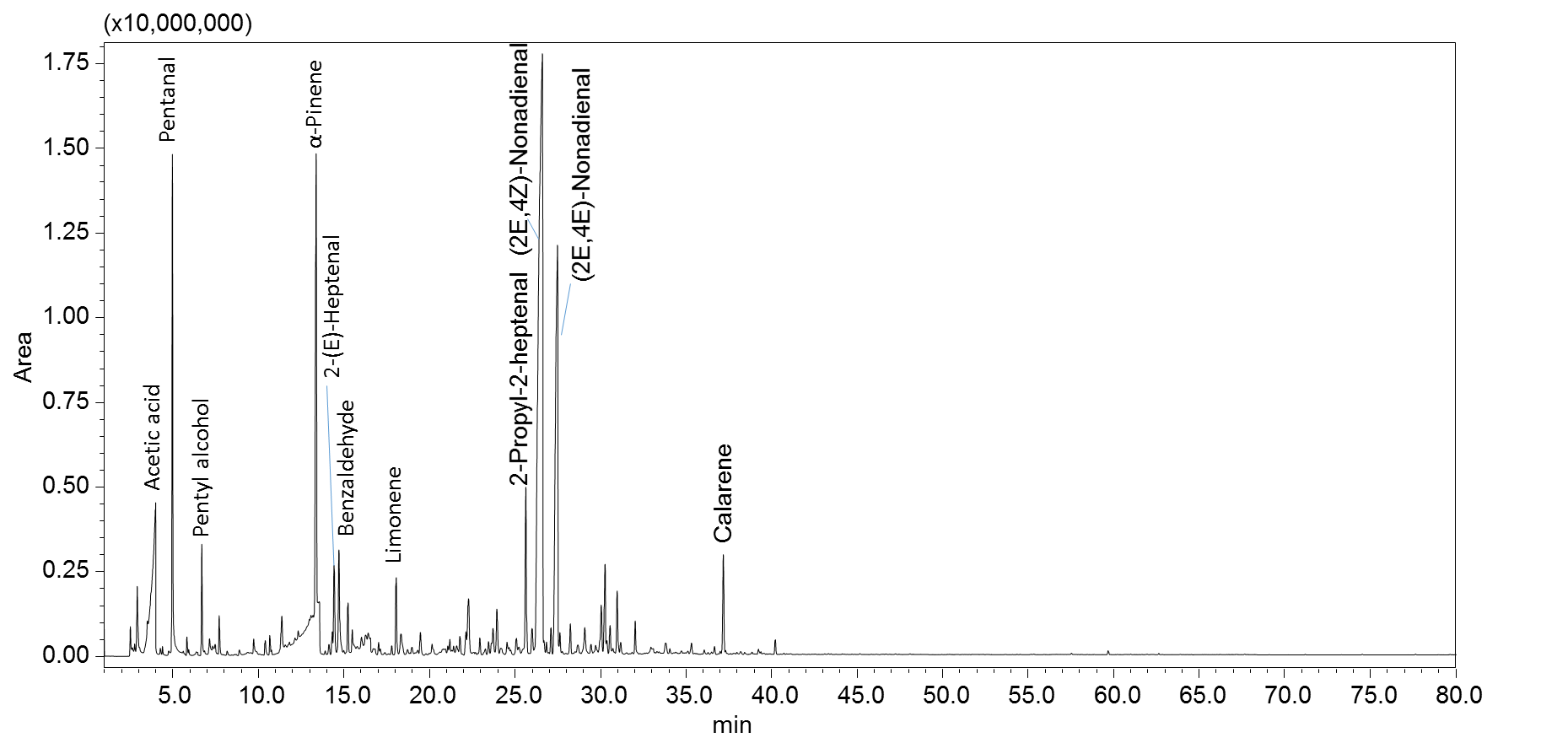
Figure S19.** Chromatogram of volatile compounds identified in pomegranate seed oil.

**
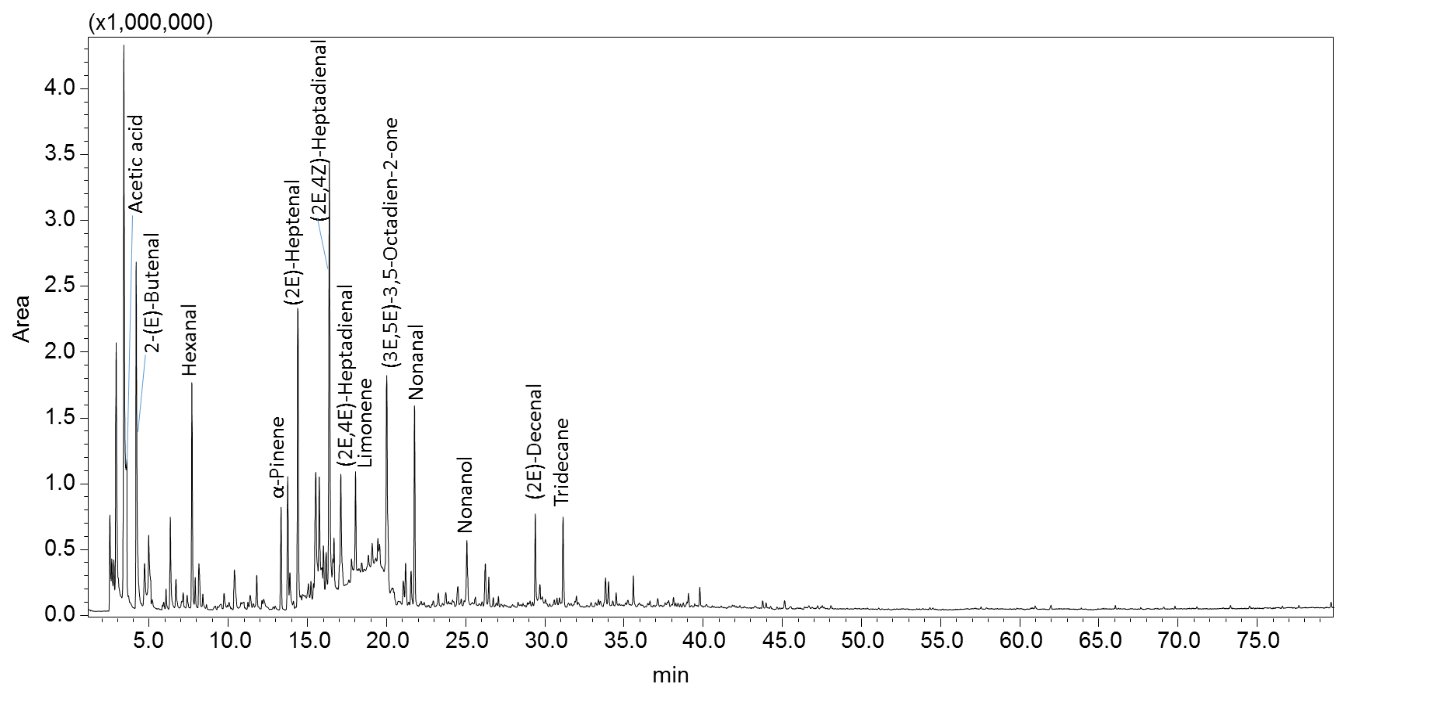
Figure S20.** Chromatogram of volatile compounds identified in radish seed oil.

**
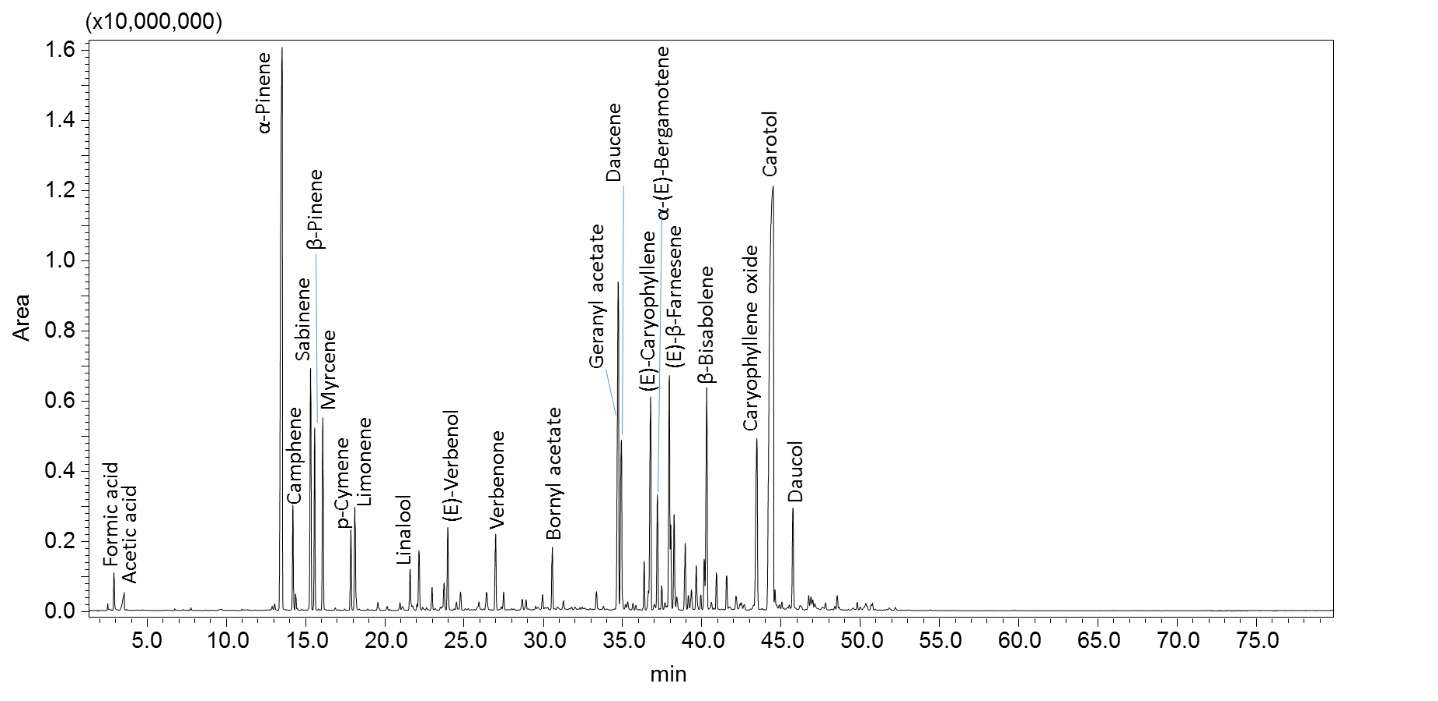
Figure S21.** Chromatogram of volatile compounds identified in carrot seed oil.


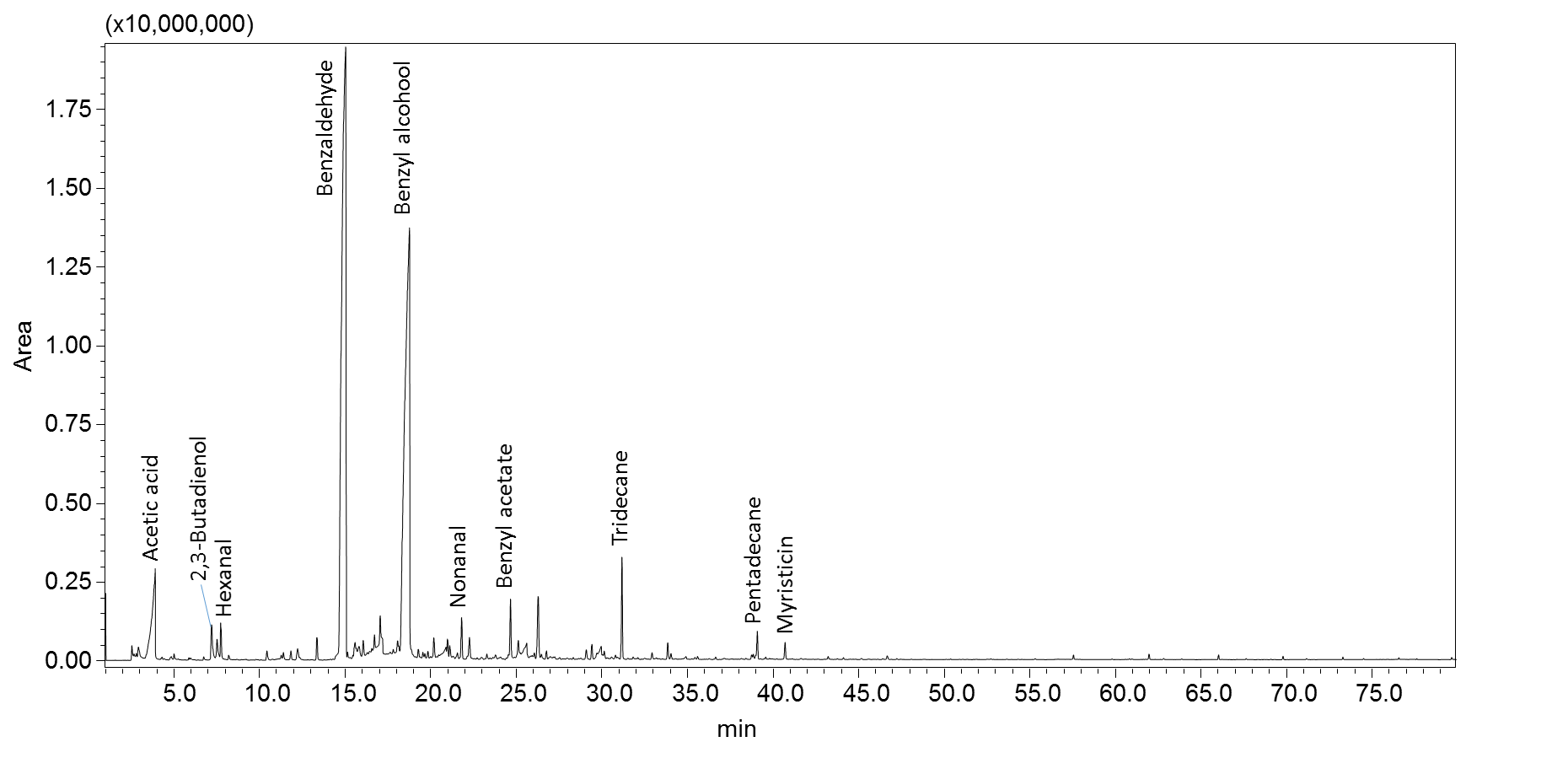


**Figure S22.** Chromatogram of volatile compounds identified in plum seed oil.

**
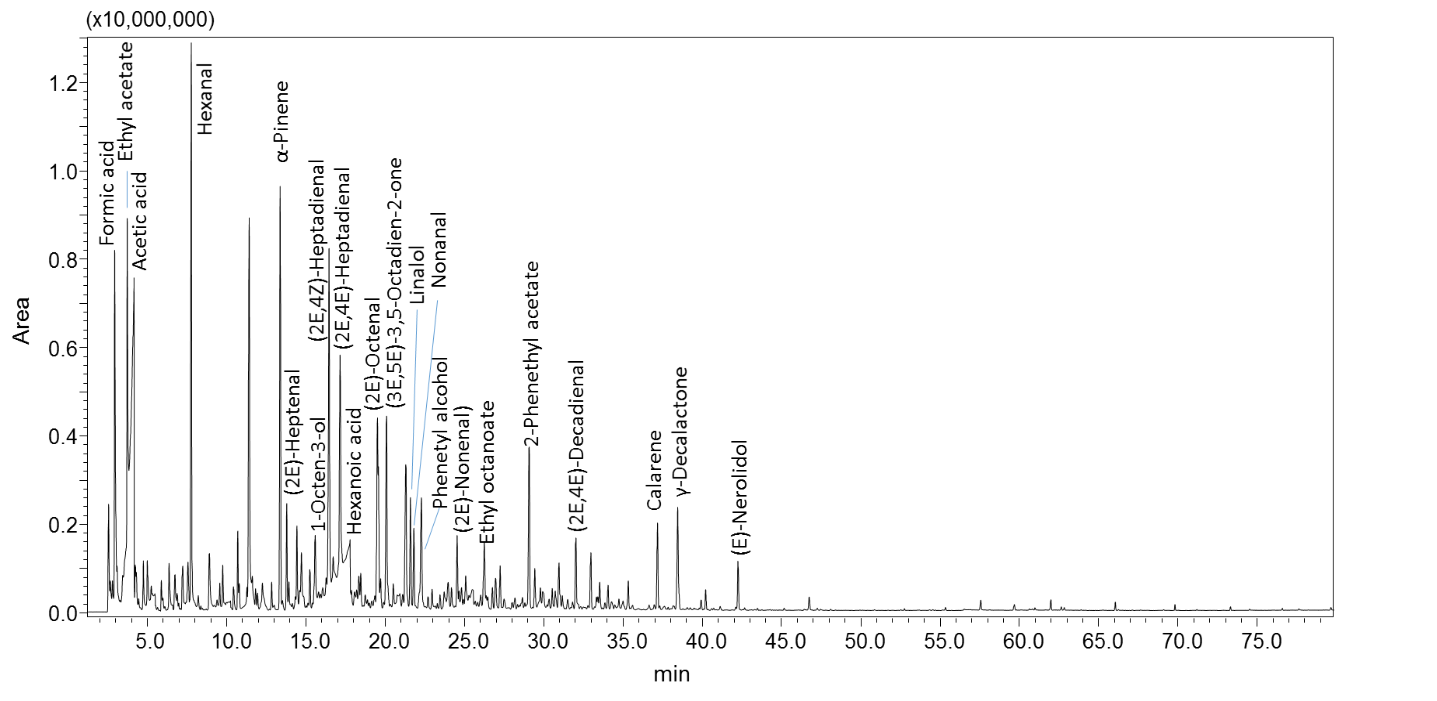
Figure S23.** Chromatogram of volatile compounds identified in strawberry seed oil.

**
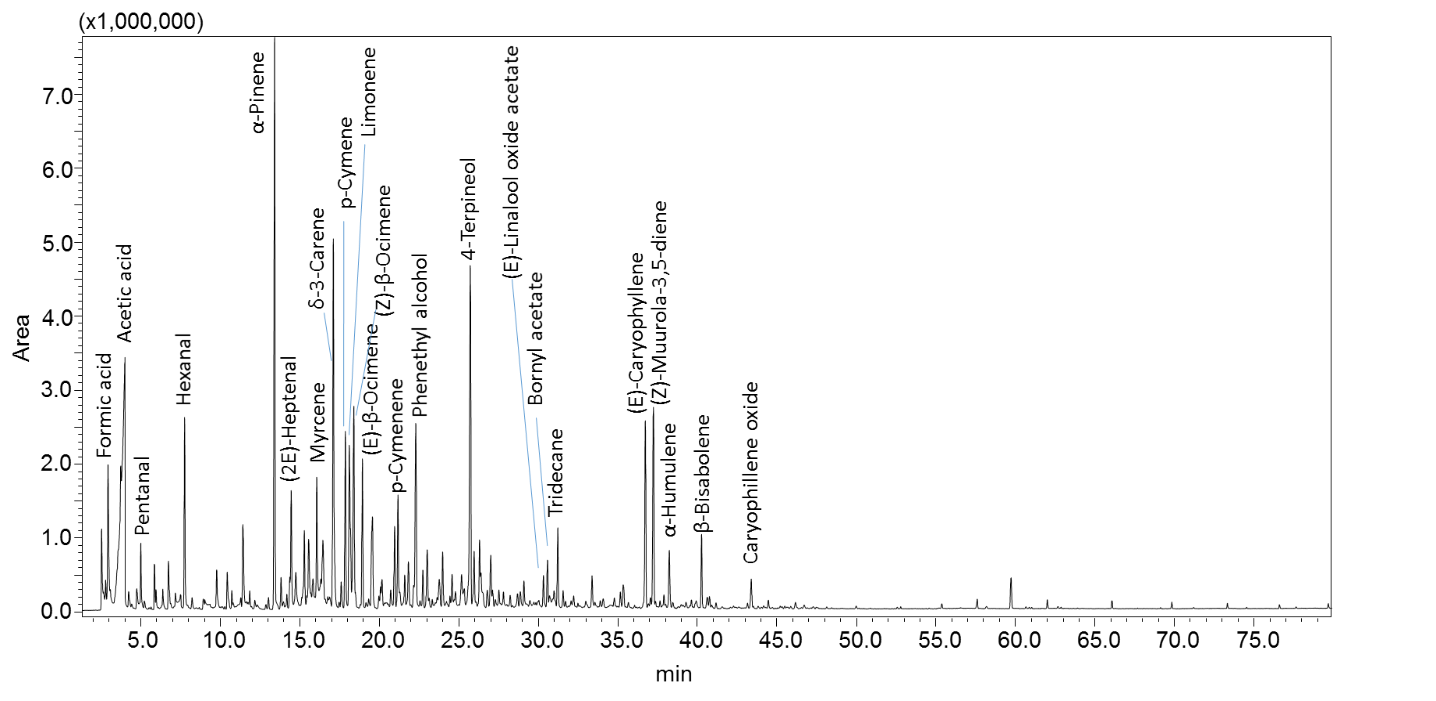
Figure S24.** Chromatogram of volatile compounds identified in blackcurrant seed oil.

**Table S2** Volatile compound identified in the fruit seed oils analysed along with experimental linear retention index (LRI exp.) and reference linear retention index (LRI ref.).

| **n** | **Compound** | **Classification** | **LRI exp** | **LRI**  **ref** | **Pomegranate** | **Raspberry** | **Rosehip** | **Radish** | **Carrot** | **Plum** | **Strawberry** | **Blackcurrant** |
| --- | --- | --- | --- | --- | --- | --- | --- | --- | --- | --- | --- | --- |
| 1 | Acetaldehyde | Aliphatic Aldehyde |  | 409 | 0.09* | 0.34* | 0.16* | 0.57* |  | 0.04* | 0.35* | 0.22* |
| 2 | Ethanol | Aliphatic Alcohol |  | 427 | 0.21 | 0.51 | 0.25 | 0.75* |  | 0.09 | 0.56* | 0.64* |
| 3 | Formic acid | Acid | 532 | 526 | 0.17 | 2.71* | 1.24 |  | 0.23* | 0.15* | 2.52* | 0.84* |
| 4 | Methyl acetate | Aliphatic Ester | 552 | 541 | 0.04* |  | 0.17* |  |  |  | 0.20* | 0.15* |
| 5 | 2-Methyl propanal | Aliphatic Aldehyde | 566 | 570 |  | 0.17* |  |  |  |  | 0.07* |  |
| 6 | n-Butanal | Aliphatic Aldehyde | 596 | 587 | 0.57* |  |  |  |  |  |  | 0.61* |
| 7 | Acetic acid | Acid | 603 | 661 | 4.05 | 13.42 | 11.79 | 2.25 | 0.32* | 2.05* | 7.72 | 6.95* |
| 8 | Vinyldimethylcarbinol | Aliphatic Alcohol | 608 | 608 |  |  |  |  | 0.01* |  |  |  |
| 9 | Ethyl acetate | Aliphatic Ester | 609 | 606 |  | 1.76 |  |  |  |  | 5.32* | 1.78* |
| 10 | (2E)-Butenal | Aliphatic Aldehyde | 645 | 650 |  | 0.18* | 0.14* | 5.13* | 0.01* | 0.01* | 0.06* | 0.34* |
| 11 | Isovaleric aldehyde | Aliphatic Aldehyde | 651 | 656 | 0.08* | 0.06 |  |  |  | 0.04* | 0.08* | 0.20* |
| 12 | Butyl alcohol | Aliphatic Alcohol | 660 | 653 | 0.14 |  |  |  |  |  |  | 0.11 |
| 13 | 2-Methylbutyraldehyde | Aliphatic Aldehyde | 664 | 662 |  | 0.04 |  |  |  |  |  |  |
| 14 | 1-Penten-3-ol | Aliphatic Alcohol | 682 | 691 |  | 0.75 |  |  |  |  | 0.58* | 0.24* |
| 15 | Propionic acid | Acid | 698 | 698 |  | 0.61 | 0.05* |  |  | 0.02* | 0.65* |  |
| 16 | α-Ethyl furan | Furan | 703 | 702 |  |  |  |  |  |  | 0.17* |  |
| 17 | n-Propyl acetate | Aliphatic Ester | 705 | 709 |  | 0.18 |  |  |  |  | 0.23* |  |
| 18 | n-Pentanal | Aliphatic Aldehyde | 707 | 696 | 14.00 | 0.94* | 0.51* | 2.54* | 0.01* | 0.10* | 0.79* | 2.17* |
| 19 | Acetoin | Aliphatic Ketone | 710 | 716 |  | 0.47* |  |  |  | 0.01* |  | 0.15* |
| 20 | Methyl butyrate | Aliphatic Ester | 721 | 718 |  |  |  |  |  |  |  | 0.05 |
| 21 | Isopentyl alcohol | Aliphatic Alcohol | 733 | 729 | 0.18 | 0.30* | 0.08* | 0.08* |  | 0.02* | 0.20* | 0.62* |
| 22 | sec-butyl-Carbinol | Aliphatic Alcohol | 736 | 733 | 0.07* | 0.19* | 0.07* | 0.18* |  | 0.03* | 0.09* | 0.35* |
| 23 | (2Z)-Pentenal | Aliphatic Aldehyde | 741 | - |  | 0.10* | 0.12* | 0.44* |  |  | 0.07* |  |
| 24 | Isobutyric acid | Acid | 744 | 774 |  |  |  |  | 0.01* |  |  | 0.02* |
| 25 | (2E)-Pentenal | Aliphatic Aldehyde | 751 | 751 | 0.06* | 0.89 | 0.52* | 2.18* |  | 0.01 | 0.76* | 0.43* |
| 26 | Pentyl alcohol | Aliphatic Alcohol | 767 | 763 | 1.8 | 0.53 |  |  |  |  | 0.40* |  |
| 27 | (2E)-Penten-1-ol | Aliphatic Alcohol | 769 | 761 |  | 0.30* |  | 0.09* |  |  | 0.40* | 0.61* |
| 28 | Isobutyl acetate | Aliphatic Ester | 770 | 768 | 0.07 | 0.12* | 0.15* |  |  |  | 0.32* | 0.21* |
| 29 | Methyl 2-methylbutyrate | Aliphatic Ester | 774 | 769 |  |  |  |  |  |  | 0.11* |  |
| 30 | 2,3-Butadienol | Aliphatic Alcohol | 782 | 788 | 0.19 | 1.96* | 0.14* | 0.69* |  | 0.59* | 0.35* | 0.10* |
| 31 | 3-Methyl crotonaldehyde | Aliphatic Aldehyde | 785 | 780 |  |  |  |  | 0.02* |  |  |  |
| 32 | Butyl methyl ketone | Aliphatic Ketone | 788 | 786 | 0.06* |  |  |  |  |  |  |  |
| 33 | Butyric acid | Acid | 795 | 818 |  |  |  |  |  |  |  | 0.2 |
| 34 | (Z)-Hex-3-enal | Aliphatic Aldehyde | 799 | 797 |  |  | 0.14* | 0.15* |  |  |  |  |
| 35 | Isobutenyl methyl ketone | Aliphatic Ketone | 800 | 797 |  |  |  |  | 0.01* |  |  |  |
| 36 | n-Octane | Hydrocarbon | 801 | 800 |  |  |  | 3.51* |  |  |  |  |
| 37 | n-Hexanal | Aliphatic Aldehyde | 803 | 801 | 0.69 | 12.96 | 5.88* | 6.93 | 0.06* | 0.75 | 10.3 | 7.03 |
| 38 | (4E)-Octene | Hydrocarbon | 806 | 796 |  |  | 0.21* | 1.13* |  |  | 0.18* |  |
| 39 | 2,4-Octadiene | Hydrocarbon | 812 | 818 |  |  |  | 0.72* |  |  |  |  |
| 40 | 4-Methyl 2-pentenal | Aliphatic Aldehyde | 817 | 814 |  |  |  |  |  |  | 0.05* |  |
| 41 | 2-Methyl 2-pentenal | Aliphatic Aldehyde | 818 | 828 |  | 0.15* |  |  |  |  |  |  |
| 42 | Methyl pentanoate | Aliphatic Ester | 822 | 821 | 0.02* |  |  |  |  |  |  |  |
| 43 | 2-Methyl pyrazine | Nitrogen compound | 825 | 820 |  |  |  |  |  | 0.01* |  |  |
| 44 | Furfural | Furan Aldehyde | 832 | 845 | 0.08 | 0.2 | 0.12 |  |  | 0.01 |  | 0.1 |
| 45 | Sclerosol | Sulfur compound | 834 | 827 |  |  |  |  |  |  |  | 0.03* |
| 46 | Isovaleric acid | Acid | 838 | 842 | 0.03 |  |  |  |  | 0.02* |  | 0.05* |
| 47 | (2Z)-Hexenal | Aliphatic Aldehyde | 841 | 842 |  | 0.04* |  |  |  |  |  |  |
| 48 | (E)-Ethyl crotonate | Aliphatic Ester | 842 | 839 |  |  |  |  |  |  |  |  |
| 49 | Ethyl 2-methylbutyrate | Aliphatic Ester | 848 | 842 |  | 0.12 |  |  |  |  | 0.25* |  |
| 50 | (2E)-Hexenal | Aliphatic Aldehyde | 851 | 850 |  | 1.85 | 0.23 | 0.28* |  | 0.01* | 0.66* | 0.52 |
| 51 | Furfuryl alcohol | Furan Alcohol | 852 | 849 |  |  | 0.02* |  |  | 0.02* |  |  |
| 52 | (3Z)-Hexenol | Aliphatic Alcohol | 853 | 853 |  | 0.23 |  |  |  |  |  |  |
| 53 | a-Methyl octane | Hydrocarbon | 861 | 863 |  |  | 0.47* |  |  |  |  |  |
| 54 | (2E)-Hexenol | Aliphatic Alcohol | 864 | 864 | 0.32 |  | 0.03* |  |  |  | 0.05* | 0.02* |
| 55 | 2-Methylbutyric acid | Acid | 867 | 881 |  | 0.12* |  |  | 0.08* |  |  |  |
| 56 | n-Hexanol | Aliphatic Alcohol | 868 | 867 | 0.22 | 0.65 | 1.92 | 0.88 |  | 0.17* | 0.29* | 0.85 |
| 57 | Isoamyl acetate | Aliphatic Ester | 876 | 871 | 0.18 | 0.58* | 0.12* |  |  |  | 0.64* | 0.27 |
| 58 | 2-Methylbutyl acetate | Aliphatic Ester | 877 | 873 | 0.05 | 0.15* | 0.06* |  |  |  | 0.21* | 0.10* |
| 59 | n-Pentanoic acid | Acid | 886 | 911 | 3.57 | 0.05* | 0.07* | 0.12 |  | 0.01* |  | 0.05* |
| 60 | Pentyl methyl ketone | Aliphatic Ketone | 889 | 887 |  | 0.21 | 0.12* | 0.25* |  | 0.06* | 0.16* | 0.12* |
| 61 | 2-Butyl furan | Furan | 891 | 890 | 0.2 |  |  |  |  |  |  |  |
| 62 | Angelic acid | Acid | 892 | 904 |  |  |  |  | 0.03* |  |  |  |
| 63 | n-Nonane | Hydrocarbon | 900 | 900 |  |  | 0.03* |  |  |  |  |  |
| 64 | (4Z)-Heptenal | Aliphatic Aldehyde | 901 | 902 |  |  |  |  |  |  |  | 0.16* |
| 65 | n-Heptanal | Aliphatic Aldehyde | 902 | 906 |  |  | 0.07 | 0.59 |  | 0.16 | 0.28* | 0.30* |
| 66 | γ-Butyrolactone | Cyclic ester/Lactone | 906 | 910 | 0.11* | 0.12* |  | 0.20* |  | 0.36* |  |  |
| 67 | 2(5H)-Furanone | Cyclic ester/Lactone | 907 | 907 |  |  | 0.03* |  |  |  |  |  |
| 68 | Sorbaldehyde | Aliphatic Aldehyde | 911 | 914 |  | 0.20* |  | 0.29* |  |  | 0.29* |  |
| 69 | Dimethyl sulphone | Sulfur compound | 912 | 913 |  |  | 0.06* |  |  |  |  |  |
| 70 | 2,5-Dimethyl pyrazine | Nitrogen compound | 913 | 912 |  |  |  |  |  | 0.02* |  |  |
| 71 | Pentyl acetate | Aliphatic Ester | 915 | 915 | 0.34* | 0.13* |  |  |  |  |  |  |
| 72 | Prenyl acetate | Aliphatic Ester | 920 | 920 |  |  |  |  |  |  | 0.02 |  |
| 73 | Methyl Hexanoate | Aliphatic Ester | 923 | 922 |  |  |  |  |  |  | 0.24 | 0.05 |
| 74 | Tricyclene | Monoterpene Hydrocarbon | 925 | 923 |  |  |  |  | 0.06* |  |  |  |
| 75 | α-Thujene | Monoterpene Hydrocarbon | 927 | 927 |  |  | 0.51* | 0.01* | 0.1 |  | 0.03* | 0.27 |
| 76 | α-Pinene | Monoterpene Hydrocarbon | 934 | 933 | 8.67 | 4.95 | 26.31 | 2.72* | 22.52 | 0.53* | 4.92* | 11.25 |
| 77 | Ethyl Tiglate | Aliphatic Ester | 938 | 938 |  |  |  |  |  |  | 0.05 |  |
| 78 | (2Z)-Heptenal | Aliphatic Aldehyde | 946 | 945 | 0.72 |  |  |  |  |  |  |  |
| 79 | Camphene | Monoterpene Hydrocarbon | 951 | 953 | 0.75* | 0.18* | 0.34 |  | 1.23 |  | 0.17* | 0.21 |
| 80 | Thuja-2,4(10)-diene | Monoterpene Hydrocarbon | 955 | 953 | 0.94* | 0.21* | 1.83* |  | 0.21 |  | 0.04* | 0.55* |
| 81 | (2E)-Heptenal | Aliphatic Aldehyde | 956 | 956 | 2.28 | 1.74 | 0.86* | 3.83 | 0.05* | 0.17 | 1.56* | 2.60* |
| 82 | 2(5H)-Furanone, 5-Ethyl | Lactone | 959 | 966 |  | 0.10* |  |  | 0.01* |  |  |  |
| 83 | Benzaldehyde | Aromatic Aldehyde | 962 | 960 | 1.29 | 1.58 | 1.76 | 0.23 | 0.02* | 54.43 | 0.73 | 0.36* |
| 84 | Isopentyl propanoate | Aliphatic Ester | 967 | 966 |  |  |  |  |  |  | 0.04* |  |
| 85 | (2E)-Heptenol | Aliphatic Alcohol | 968 | 964 | 0.02* |  |  |  |  |  |  |  |
| 86 | n-Heptanol | Aliphatic Alcohol | 971 | 970 |  | 0.04 |  | 0.46* |  | 0.07* | 0.04* | 0.42* |
| 87 | 3,7,7-Trimethyl-1,3,5-cycloheptatriene | Monoterpene Hydrocarbon | 973 | 980 |  |  |  |  |  |  |  | 1.08* |
| 88 | Sabinene | Monoterpene Hydrocarbon | 975 | 972 | 0.61* | 0.61* | 3.91* | 0.11* | 3.51 | 0.16* | 0.45* | 0.06* |
| 89 | 1-Octen-3-one | Aliphatic Ketone | 978 | 973 |  | 0.05* | 1.24* | 0.39* |  | 0.18* | 0.04* | 0.82* |
| 90 | β-Pinene | Monoterpene Hydrocarbon | 980 | 978 | 0.27 | 0.35 | 0.39 |  | 2.31 | 0.03* | 0.23* | 0.47 |
| 91 | Vinyl amyl carbinol | Aliphatic Alcohol | 981 | 978 |  | 0.91 |  |  |  |  | 0.89* | 0.09* |
| 92 | 6-Methyl Hept-5-en-2-one | Aliphatic Ketone | 984 | 986 |  | 0.29 | 0.52 | 2.15 | 0.03* |  |  | 0.49* |
| 93 | 2-Pentylfuran | Furan | 990 | 991 |  |  | 0.61 |  |  |  | 0.19* |  |
| 94 | Myrcene | Monoterpene Hydrocarbon | 991 | 991 | 0.16 |  | 0.34 | 0.56* | 3.06 |  |  | 2.52 |
| 95 | 6-Methyl Hept-5-en-2-ol | Aliphatic Alcohol | 994 | 995 |  |  | 0.63* | 1.17* | 0.02* |  |  |  |
| 96 | Mesitylene | Hydrocarbon | 996 | 994 |  |  | 0.29* |  |  | 0.22* |  | 0.20* |
| 97 | n-Hexanoic acid | Acid | 998 | 997 | 0.29 | 0.76* |  | 0.71 |  | 0.78 | 1.26* | 0.19 |
| 98 | (2E,4Z)-Heptadienal | Aliphatic Aldehyde | 999 | 1001 |  | 4.41* | 2.29* | 5.43* |  |  | 3.12* | 1.06* |
| 99 | Ethyl hexanoate | Aliphatic Ester | 1000 | 1003 |  |  |  |  |  |  | 2.24* | 0.15 |
| 100 | n-Decane | Hydrocarbon | 1001 | 1000 |  |  | 0.30* |  |  | 0.35* |  |  |
| 101 | Octan-2-ol | Aliphatic Alcohol | 1002 | 1004 |  |  |  | 1.07* |  |  |  |  |
| 102 | n-Octanal | Aliphatic Aldehyde | 1004 | 1006 |  | 0.36 | 0.26 | 0.90* |  | 0.73 | 0.49* | 0.31* |
| 103 | (3Z)-hexenyl acetate | Aliphatic Ester | 1005 | 1008 |  | 0.39* |  |  |  |  |  |  |
| 104 | δ-3-Carene | Monoterpene Hydrocarbon | 1010 | 1009 | 0.41* |  | 0.07* | 1.20* |  | 0.22* |  | 8.47 |
| 105 | α-Phellandrene | Monoterpene Hydrocarbon | 1011 | 1007 |  |  |  |  |  |  | 0.42* |  |
| 106 | Hexyl acetate | Aliphatic Ester | 1012 | 1012 | 0.11 |  |  |  |  |  |  |  |
| 107 | (2E,4E)-Heptadienal | Aliphatic Aldehyde | 1014 | 1013 |  | 2.15* | 0.30* | 0.55* |  |  | 1.43* | 0.31* |
| 108 | (2E)-Hexenyl acetate | Aliphatic Ester | 1015 | 1017 |  |  |  |  |  |  | 1.74* |  |
| 109 | α-Terpinene | Monoterpene Hydrocarbon | 1019 | 1018 |  |  | 0.04* |  | 0.03 |  |  | 0.24 |
| 110 | (3Z)-Hexenoic acid | Acid | 1021 | 1011 |  | 0.23* |  |  |  |  |  |  |
| 111 | Pseudocumene | Monoterpene Hydrocarbon | 1022 | 1020 |  |  | 0.08* |  |  |  |  |  |
| 112 | o-Cymene | Monoterpene Hydrocarbon | 1023 | 1024 |  |  |  |  |  |  |  | 0.49 |
| 113 | (2E,4E)-Heptadienol | Aliphatic Alcohol | 1024 | 1019 |  | 0.61* |  |  |  |  |  |  |
| 114 | p-Cymene | Monoterpene Hydrocarbon | 1027 | 1025 | 0.18 | 1 | 1.25* | 0.72* | 1.07 | 0.16* |  | 4.44 |
| 115 | Limonene | Monoterpene Hydrocarbon | 1031 | 1030 | 1.44 | 0.89 | 2.33 | 2.34* | 1.36 | 0.36* | 1.00* | 3.34 |
| 116 | (Z)-β-Ocimene | Monoterpene Hydrocarbon | 1032 | 1035 | 0.05* |  |  |  |  |  |  | 2.37 |
| 117 | β-Phellandrene | Monoterpene Hydrocarbon | 1033 | 1031 |  |  | 0.09* |  | 0.1 | 0.17* |  | 1.43 |
| 118 | Benzyl alcohol | Aromatic Alcohol/Phenol | 1035 | 1040 | 0.26* | 0.3 | 0.47 | 0.33* |  | 29.90* | 0.73* | 0.87* |
| 119 | Oct-3-en-2-one | Aliphatic Ketone | 1039 | 1036 | 0.1 | 0.44* | 0.09* | 0.23* |  |  | 0.87* | 0.13* |
| 120 | Phenylacetaldehyde | Aromatic Aldehyde | 1044 | 1045 |  | 0.07 |  |  |  |  | 0.15 | 0.01* |
| 121 | (E)-β-Ocimene | Monoterpene Hydrocarbon | 1047 | 1046 |  |  |  |  |  |  |  | 1.27 |
| 122 | (2Z)-Octenal | Aliphatic Aldehyde | 1048 | 1047 |  | 0.14 | 0.06* |  |  |  | 0.10* |  |
| 123 | γ-Hexalactone | Cyclic ester/Lactone | 1051 | 1060 |  | 0.06* |  | 0.77* |  | 0.01* | 0.07* |  |
| 124 | Isopentyl butyrate | Aliphatic Ester | 1056 | 1054 | 0.10* |  |  |  |  |  |  |  |
| 125 | (2E)-Octenal | Aliphatic Aldehyde | 1059 | 1058 | 0.44 | 2.02* | 0.64* | 0.78 |  | 0.03 | 3.15* | 0.37 |
| 126 | γ-Terpinene | Monoterpene Hydrocarbon | 1061 | 1058 |  |  |  |  | 0.09 |  |  | 1.3 |
| 127 | (4Z)-Octenol | Aliphatic Alcohol | 1064 | 1061 |  |  | 0.16* |  |  |  |  |  |
| 128 | 2-Acetyl pyrrole | Aromatic Ketone | 1066 | 1060 | 0.02 |  |  |  |  | 0.01* |  |  |
| 129 | Acetophenone | Aromatic Ketone | 1067 | 1068 |  | 0.29* |  |  |  | 0.07* | 0.07* |  |
| 130 | (2E)-Octenol | Aliphatic Alcohol | 1068 | 1067 |  |  | 0.05* |  |  |  |  |  |
| 131 | (2Z)-Octenol | Aliphatic Alcohol | 1069 | 1067 | 0.01* |  |  |  |  | 0.02* |  | 0.11* |
| 132 | (3E,5E)-3,5-Octadien-2-one | Aliphatic Ketone | 1070 | 1073 |  | 3.49* | 0.51 | 2.36* |  |  | 2.14* | 0.13* |
| 133 | n-Octanol | Aliphatic Alcohol | 1072 | 1076 |  |  | 0.08 | 0.73 |  | 0.21* | 0.12* | 0.19* |
| 134 | (Z)-Sabinene hydrate | Monoterpene Alcohol | 1073 | 1069 |  |  |  |  | 0.04 |  |  |  |
| 135 | n-Heptanoic acid | Acid | 1078 | 1116 |  |  |  | 0.24* |  | 0.16* | 0.40* | 0.05* |
| 136 | p-Cresol | Aromatic Alcohol/Phenol | 1079 | 1072 |  |  |  |  | 0.02* |  |  | 0.03* |
| 137 | Benzyl formate | Aromatic Ester | 1080 | 1078 |  |  |  |  |  | 0.06* |  |  |
| 138 | m-Cymenene | Monoterpene Hydrocarbon | 1084 | 1080 |  |  |  |  |  |  |  | 0.22* |
| 139 | Terpinolene | Monoterpene Hydrocarbon | 1087 | 1086 |  |  |  |  |  |  |  | 1.42 |
| 140 | (E)-Linalool oxide | Oxygenated Monoterpene Alcohol | 1088 | 1086 |  | 0.14 |  |  |  | 0.26* |  |  |
| 141 | p-Cymenene | Monoterpene Hydrocarbon | 1092 | 1093 |  |  |  |  |  |  |  | 1.05* |
| 142 | Pentyl butyrate | Aliphatic Ester | 1093 | 1095 | 0.18* |  |  |  |  |  |  |  |
| 143 | 3,5-Octadien-2-one ISOMER | Aliphatic Ketone | 1094 | 1091 |  |  |  | 0.61* |  |  |  |  |
| 144 | (4E)-Nonenal | Aliphatic Aldehyde | 1095 | 1098 |  |  | 0.14* |  |  |  |  |  |
| 145 | Methylbenzoate | Aromatic Ester | 1096 | 1096 |  |  |  |  |  | 0.04* |  |  |
| 146 | n-Undecane | Hydrocarbon | 1100 | 1100 |  |  | 0.35* | 0.31 |  | 0.09* |  |  |
| 147 | α-Pinene oxide | Oxygenated monoterpene Hydrocarbon | 1101 | 1101 | 0.07* |  | 0.23* |  |  |  |  |  |
| 148 | Linalool | Monoterpene Alcohol | 1102 | 1101 |  | 0.27 |  |  | 0.48 |  | 0.95 | 0.2 |
| 149 | Nonan-2-ol | Aliphatic Alcohol | 1103 | 1105 |  |  |  |  |  | 0.01* |  |  |
| 150 | 6-Methyl Heptadi-3,5-en-2-one | Aliphatic Ketone | 1105 | 1102 |  |  |  |  | 0.02* |  |  |  |
| 151 | Nonanal | Aliphatic Aldehyde | 1107 | 1107 | 0.27 | 0.64 | 0.42 | 1.59 |  | 0.41 | 0.78 | 0.33 |
| 152 | α-Thujone | Monoterpene Ketone | 1109 | 1110 |  |  |  |  |  |  |  | 0.07 |
| 153 | Maltol | Aromatic Ketone | 1113 | 1108 |  |  |  |  |  | 0.02* |  |  |
| 154 | Phenethyl alcohol | Aromatic Alcohol | 1116 | 1113 | 0.72 | 1.28 | 0.23* | 0.06* |  | 0.20* | 0.84* | 1.17* |
| 155 | β-Thujone | Monoterpene Ketone | 1121 | 1118 |  |  |  |  |  |  |  | 0.10* |
| 156 | Dehydro-sabina ketone | Monoterpene Ketone | 1122 | 1122 |  |  |  |  | 0.04* |  |  |  |
| 157 | Methyl octanoate | Aliphatic Ester | 1125 | 1125 | 0.04* | 0.02* |  |  |  | 0.01* | 0.10* | 0.18* |
| 158 | α-Campholenal | Monoterpene Aldehyde | 1129 | 1125 | 0.19* | 0.14* | 0.86* |  | 0.20* |  | 0.18* | 0.72 |
| 159 | (4E,6Z)-allo-Ocimene | Monoterpene Hydrocarbon | 1130 | 1128 |  |  |  |  |  |  |  | 0.15* |
| 160 | Limona ketone | Oxygenated Monoterpene Hydrocarbon | 1134 | 1131 |  |  |  |  | 0.03* |  |  |  |
| 161 | Non-3-en-2-one | Aliphatic Ketone | 1139 | 1137 |  | 0.07* |  |  |  |  | 0.10* |  |
| 162 | (E)-Pinocarveol | Monoterpene Alcohol | 1145 | 1141 | 0.34* | 0.17* | 1.17* |  | 0.33 |  | 0.18* | 0.26* |
| 163 | (2Z)-Nonenal | Aliphatic Aldehyde | 1147 | 1148 |  | 0.03* |  |  |  |  |  |  |
| 164 | (E)-Verbenol | Monoterpene Alcohol | 1149 | 1145 | 0.56* | 0.23* | 1.30* | 0.08* | 0.96 |  | 0.22* | 0.39* |
| 165 | γ-Heptalactone | Cyclic ester/Lactone | 1152 | 1155 |  |  |  |  |  | 0.01* |  |  |
| 166 | Isopentyl valerate | Aliphatic Ester | 1153 | 1151 | 0.05* |  |  |  |  |  |  |  |
| 167 | (2E,6Z)-Nonadienal | Aliphatic Aldehyde | 1154 | 1153 |  | 0.41 |  |  |  |  | 0.13* |  |
| 168 | (2E,6E)-Nonadienal | Aliphatic Aldehyde | 1155 | 1152 |  |  |  |  |  |  |  | 0.05* |
| 169 | Menthone | Monoterpene Ketone | 1158 | 1158 |  | 0.11* | 0.02* |  |  |  |  | 0.07* |
| 170 | Sabina ketone | Monoterpene Ketone | 1160 | 1157 |  |  | 0.04* |  | 0.09 |  |  |  |
| 171 | (2E)-Nonenal | Aliphatic Aldehyde | 1161 | 1163 | 0.09 | 1.28 | 0.08* | 0.15* |  | 0.02* | 0.47* | 0.16* |
| 172 | (E)-Pinocamphone | Monoterpene Ketone | 1163 | 1160 |  |  | 0.22* |  | 0.02* |  |  |  |
| 173 | Benzyl acetate | Aromatic Ester | 1164 | 1167 |  | 0.05* |  |  |  | 0.45* | 0.11* |  |
| 174 | Pinocarvone | Monoterpene Ketone | 1166 | 1164 | 0.10* |  | 0.25* |  | 0.22 |  |  |  |
| 175 | 4-Ethyl phenol | Aromatic Alcohol/Phenol | 1167 | 1165 |  |  |  |  |  |  | 0.07* |  |
| 176 | Ethyl enzoate | Aromatic Ester | 1172 | 1170 |  | 0.08* |  |  |  |  | 0.16* |  |
| 177 | 1-Nonanol | Aliphatic Alcohol | 1173 | 1176 |  | 0.12 | 0.16 | 0.46 |  | 0.14* | 0.08* | 0.15* |
| 178 | Borneol | Monoterpene Alcohol | 1175 | 1173 |  |  | 0.03* |  | 0.02 |  |  |  |
| 179 | (Z)-Pinocamphone | Monoterpene Ketone | 1179 | 1176 |  |  | 0.07* |  |  |  |  |  |
| 180 | Menthol | Monoterpene Alcohol | 1181 | 1184 |  | 0.04* |  |  |  |  |  |  |
| 181 | Octanoic acid | Acid | 1182 | 1166 |  | 0.13* |  |  |  | 0.24* | 0.24 |  |
| 182 | Terpinen-4-ol | Monoterpene Alcohol | 1183 | 1184 |  |  | 0.05* | 0.09* |  |  |  | 3.25 |
| 183 | 2-Propyl-2-heptenal | Aliphatic Aldehyde | 1184 | - | 2.16* |  |  |  |  |  |  |  |
| 184 | 4’-Methyl acetophenone | Aromatic Ketone | 1189 | 1188 |  |  |  |  | 0.03* |  |  |  |
| 185 | p-Cymen-8-ol | Monoterpene Alcohol | 1190 | 1189 |  |  |  |  |  |  |  | 0.29 |
| 186 | Pentyl pentanoate | Aliphatic Ester | 1191 | 1194 | 0.26 |  |  |  |  |  |  |  |
| 187 | Hexyl butyrate | Aliphatic Ester | 1192 | 1195 |  |  |  |  |  |  | 0.12* |  |
| 188 | Creosol | Aromatic Alcohol/Phenol | 1193 | 1190 |  | 0.04* |  |  |  |  |  |  |
| 189 | (Z)-Pinocarveol | Monoterpene Alcohol | 1194 | 1186 |  |  | 0.05* |  |  |  |  |  |
| 190 | Octyl methyl ketone | Aliphatic Ketone | 1195 | 1196 |  |  |  |  |  | 0.05* |  |  |
| 191 | Methyl salicylate | Aromatic Ester | 1196 | 1192 |  |  |  |  |  |  | 0.45* |  |
| 192 | p-Mentha-1,5-dien-7-ol | Monoterpene Alcohol | 1197 | 1194 |  |  |  |  | 0.01* |  |  |  |
| 193 | Ethyl octanoate | Aliphatic Ester | 1198 | 1202 |  |  |  |  |  |  | 0.45* | 0.23 |
| 194 | (2E)-Hexenyl butyrate | Aliphatic Ester | 1199 | 1197 |  | 0.20* |  |  |  |  |  |  |
| 195 | 2-Hydroxy-6-methylbenzaldehyde | Aromatic Aldehyde | 1200 | - |  |  |  |  |  | 0.52* |  |  |
| 196 | Myrtenal | Monoterpene Aldehyde | 1202 | 1197 |  |  | 0.16* |  | 0.18 |  |  |  |
| 197 | α-Terpineol | Monoterpene Alcohol | 1203 | 1195 |  | 0.09 |  |  |  |  | 0.14* | 0.24 |
| 198 | Myrtenol | Monoterpene Alcohol | 1204 | 1202 |  |  | 0.28* |  | 0.1 |  |  | 0.09 |
| 199 | n-Dodecane | Hydrocarbon | 1205 | 1200 |  | 0.06* | 0.03* | 0.16* |  | 0.04* | 0.05* | 0.03* |
| 200 | (2E,4Z)-Nonadienal | Aliphatic Aldehyde | 1206 | - | 29.16 |  |  |  |  |  |  |  |
| 201 | n-Decanal | Aliphatic Aldehyde | 1208 | 1208 |  | 0.1 |  | 0.04 |  | 0.04* | 0.09* |  |
| 202 | Octyl acetate | Aliphatic Ester | 1210 | 1214 |  |  |  |  |  | 0.01* |  |  |
| 203 | Verbenone | Monoterpene Ketone | 1212 | 1208 | 0.23* | 0.26* | 0.91 | 0.01* | 1.12 | 0.03* | 0.21* | 0.44* |
| 204 | (2E,4E)-Nonadienal | Aliphatic Aldehyde | 1217 | 1218 | 9 | 0.09 | 0.03* | 0.01* |  | 0.02* | 0.13* | 0.05* |
| 205 | (E)-Carveol | Monoterpene Alcohol | 1223 | 1223 | 0.13* | 0.05* | 0.26* |  | 0.24 |  | 0.08* | 0.09* |
| 206 | Methyl nonanoate | Aliphatic Ester | 1224 | 1224 |  |  |  |  |  | 0.01* |  |  |
| 207 | Nerol | Monoterpene Alcohol | 1227 | 1229 |  |  |  |  | 0.01* |  |  |  |
| 208 | Citronellol | Monoterpene Alcohol | 1229 | 1232 |  |  |  |  |  |  |  | 0.02 |
| 209 | Benzosulfonazole | Sulfur compound | 1230 | 1226 |  |  |  |  |  |  | 0.02* |  |
| 210 | (Z)-Carveol | Monoterpene Alcohol | 1236 | 1232 |  |  |  |  | 0.02 |  |  |  |
| 211 | Neral | Monoterpene Aldehyde | 1240 | 1238 |  |  |  | 0.05* |  | 0.01* |  |  |
| 212 | Ethyl phenylacetate | Aromatic Ester | 1243 | 1246 |  |  |  |  |  |  | 0.02* |  |
| 213 | Carvone | Monoterpene Ketone | 1247 | 1246 |  |  | 0.10* | 0.05* | 0.18 | 0.01* |  | 0.09* |
| 214 | Isopentyl hexanoate | Aliphatic Ester | 1250 | 1252 |  |  |  |  |  |  | 0.03* |  |
| 215 | Linalyl acetate | Monoterpene Ester | 1251 | 1250 |  |  |  |  | 0.02* |  |  |  |
| 216 | Geraniol | Monoterpene Alcohol | 1253 | 1255 |  |  |  |  | 0.12 |  |  |  |
| 217 | 2-Phenethyl acetate | Aromatic Ester | 1255 | 1257 | 0.17 | 0.23* |  |  |  |  | 0.71* | 0.08* |
| 218 | γ-Octalactone | Cyclic ester/Lactone | 1256 | 1263 |  |  |  | 0.02* |  | 0.05* |  |  |
| 219 | (2E)-Decenal | Aliphatic Aldehyde | 1263 | 1265 | 0.05 | 0.04 | 0.03* | 0.38* |  | 0.05* | 0.11* | 0.02* |
| 220 | Nonanoic acid | Acid | 1269 | 1264 |  | 0.09* | 0.04 | 0.06 |  | 0.12* | 0.06 | 0.04* |
| 221 | Geranial | Monoterpene Aldehyde | 1271 | 1268 |  |  |  |  |  | 0.06* |  |  |
| 222 | Ethyl salicylate | Aromatic Ester | 1272 | 1270 |  |  |  |  |  |  |  | 0.03* |
| 223 | Neryl formate | Monoterpene Ester | 1277 | 1276 |  |  |  | 0.06* |  |  |  |  |
| 224 | (E)-Carvone oxide | Oxygenated Monoterpene Ketone | 1280 | 1277 |  |  |  |  | 0.02* |  |  |  |
| 225 | (E)-Linalool oxide acetate | Oxygenated Monoterpene Ester | 1283 | 1290 |  |  |  |  |  |  |  | 0.13 |
| 226 | Bornyl acetate | Monoterpene Ester | 1288 | 1285 | 0.21* | 0.13* | 0.65* |  | 0.53 |  | 0.13* | 0.26 |
| 227 | (E)-Sabinyl acetate | Monoterpene Ester | 1291 | 1291 |  |  |  |  |  |  |  | 0.04* |
| 228 | (E)-Verbenyl acetate | Monoterpene Ester | 1292 | 1289 |  |  | 0.07* |  |  |  |  |  |
| 229 | n-Tridec-1-ene | Hydrocarbon | 1293 | 1292 |  |  |  | 0.04* |  |  | 0.07* |  |
| 230 | Nonyl methyl ketone | Aliphatic Ketone | 1294 | 1294 |  |  |  |  |  | 0.01* |  |  |
| 231 | Ethyl nonanoate | Aliphatic Ester | 1295 | 1297 |  |  |  |  |  | 0.01* |  |  |
| 232 | p-Cymen-7-ol | Monoterpene Alcohol | 1296 | 1291 |  |  |  |  | 0.04* |  |  |  |
| 233 | (E,Z)-2,4-Decadienal | Aliphatic Aldehyde | 1297 | 1295 | 0.41* | 0.10* | 0.01 |  |  |  | 0.16* | 0.03* |
| 234 | (E)-Pinocarvyl acetate | Monoterpene Ester | 1298 | 1296 |  |  | 0.19* |  |  |  |  | 0.07* |
| 235 | n-Tridecane | Hydrocarbon | 1300 | 1300 | 0.07* | 0.14* | 0.11 | 0.37* |  | 0.42* | 0.07* | 0.19* |
| 236 | n-Undecanal | Aliphatic Aldehyde | 1308 | 1309 |  |  | 0.01* | 0.01* |  |  |  |  |
| 237 | Isoascaridole | Oxygenated monoterpene Hydrocarbon | 1310 | 1306 |  |  |  |  |  |  |  | 0.07* |
| 238 | (2E,4E)-Decadienal | Aliphatic Aldehyde | 1321 | 1322 | 0.18* | 0.11* | 0.08* | 0.05* |  |  | 0.20* | 0.03* |
| 239 | Methyl decanoate | Aliphatic Ester | 1324 | 1327 |  |  |  |  |  |  |  | 0.02* |
| 240 | Myrtenyl acetate | Monoterpene Ester | 1325 | 1324 |  |  | 0.01* |  |  |  |  |  |
| 241 | α-Terpinyl acetate | Monoterpene Ester | 1348 | 1349 |  |  |  |  | 0.17* | 0.01* |  |  |
| 242 | Ethyl hydrocinnamate | Aromatic Ester | 1349 | 1352 |  |  |  |  |  |  | 0.03* |  |
| 243 | Citronellyl acetate | Monoterpene Ester | 1350 | 1350 |  |  |  |  |  |  |  | 0.09 |
| 244 | Phenethyl propionate | Aromatic Ester | 1351 | 1355 |  |  |  |  |  |  | 0.03* |  |
| 245 | α-Cubebene | Sesquiterpene Hydrocarbon | 1352 | 1347 |  |  | 0.01* |  |  |  |  |  |
| 246 | Eugenol | Sesquiterpene Alcohol | 1354 | 1357 |  |  |  |  |  | 0.01* |  |  |
| 247 | α-Longipinene | Sesquiterpene Hydrocarbon | 1356 | 1352 |  |  | 0.01* |  |  |  |  |  |
| 248 | γ-Nonalactone | Cyclic Ester/Lactone | 1361 | 1362 |  | 0.04* | 0.01* | 0.16* |  | 0.06* | 0.03* | 0.02* |
| 249 | (8Z)-Undecenal | Aliphatic Aldehyde | 1365 | 1365 | 0.05 | 0.02* | 0.01* | 0.03* |  |  | 0.05* |  |
| 250 | (2E)-Undecenal | Aliphatic Aldehyde | 1366 | 1357 |  |  |  |  |  | 0.02* |  |  |
| 251 | Hydrocinnamyl acetate | Aromatic Ester | 1370 | 1375 |  |  |  |  |  |  | 0.03* |  |
| 252 | 2-Butyl-2-octen-1-al | Aliphatic Aldehyde | 1373 | 1369 |  | 0.04* |  |  |  |  | 0.02* |  |
| 253 | Cyclosativene | Sesquiterpene Hydrocarbon | 1374 | 1367 |  |  | 0.03* |  |  |  |  |  |
| 254 | α-Ylangene | Sesquiterpene Hydrocarbon | 1376 | 1371 |  | 0.01* |  |  |  |  |  |  |
| 255 | Geranyl acetate | Monoterpene Ester | 1379 | 1380 |  |  |  |  | 4.92 |  |  | 0.01* |
| 256 | α-Ionol | Aliphatic Alcohol | 1380 | 1376 |  | 0.02* |  |  |  |  |  |  |
| 257 | α-Copaene | Sesquiterpene Hydrocarbon | 1382 | 1375 |  | 0.17* | 0.04 |  | 0.02 |  |  | 0.04* |
| 258 | Daucene | Sesquiterpene Hydrocarbon | 1384 | 1384 |  |  |  |  | 2.65 |  |  |  |
| 259 | Hexyl hexanoate | Aliphatic Ester | 1385 | 1390 |  |  |  |  |  | 0.01* |  |  |
| 260 | (E)-p-Menth-6-en-2,8-diol | Monoterpene Alcohol | 1386 | 1375 |  |  |  |  | 0.08* |  |  |  |
| 261 | (E)-Cinnamate methyl | Aromatic Ester | 1387 | 1382 |  |  |  |  |  |  | 0.04* |  |
| 262 | β-Bourbonene | Sesquiterpene Hydrocarbon | 1389 | 1382 | 0.01* |  | 0.02* |  |  |  |  |  |
| 263 | 7-epi-Sesquithujene | Sesquiterpene Hydrocarbon | 1392 | 1387 |  |  |  |  | 0.05* |  |  |  |
| 264 | β-Elemene | Sesquiterpene Hydrocarbon | 1393 | 1390 |  |  | 0.07* |  | 0.07 |  |  | 0.07* |
| 265 | Ethyl decanoate | Aliphatic Ester | 1394 | 1399 | 0.04 |  |  |  |  |  |  |  |
| 266 | Phenethyl isobutyrate | Aromatic Ester | 1395 | 1395 |  | 0.05* |  |  |  |  | 0.07* |  |
| 267 | Vanillin | Phenolic Aldehyde | 1397 | 1394 |  | 0.01* | 0.01* |  |  | 0.01* |  |  |
| 268 | n-Tetradecane | Hydrocarbon | 1400 | 1400 | 0.01* | 0.02* | 0.02 | 0.07* |  | 0.01* | 0.02* | 0.01* |
| 269 | α-Funebrene | Sesquiterpene Hydrocarbon | 1406 | 1403 |  |  | 0.01* |  | 0.05* |  |  |  |
| 270 | Decyl acetate | Aliphatic Ester | 1408 | 1412 |  |  |  |  |  |  | 0.01* |  |
| 271 | α-Gurjunene | Sesquiterpene Hydrocarbon | 1409 | 1406 |  | 0.08* |  |  |  |  |  |  |
| 272 | n-Dodecanal | Aliphatic Aldehyde | 1410 | 1410 |  | 0.01* | 0.01 |  |  | 0.01* | 0.01* |  |
| 273 | α-Dihydroionone | Monoterpene Ketone | 1417 | 1412 |  | 0.01* |  |  |  |  |  |  |
| 274 | α-(Z)-Bergamotene | Sesquiterpene Hydrocarbon | 1416 | 1416 |  | 0.04* |  |  | 0.43 | 0.01* |  |  |
| 275 | (Z)-Caryophyllene | Sesquiterpene Hydrocarbon | 1417 | 1413 | 0.03 |  |  |  |  |  |  |  |
| 276 | α-Santalene | Sesquiterpene Hydrocarbon | 1424 | 1418 |  |  |  |  | 0.16* |  |  |  |
| 277 | β-Maaliene | Sesquiterpene Hydrocarbon | 1425 | 1418 |  |  | 0.12* |  |  |  |  |  |
| 278 | (E)-Caryophyllene | Sesquiterpene Hydrocarbon | 1426 | 1424 | 0.07 |  |  | 0.04* | 2.91 | 0.01* |  | 1.07 |
| 279 | (E)-α-Ionone | Monoterpene Ketone | 1427 | 1421 |  | 0.17 |  |  |  |  |  |  |
| 280 | Octyl 2-methylbutyrate | Aliphatic Ester | 1432 | 1431 |  |  |  |  |  |  | 0.02* |  |
| 281 | γ-Elemene | Sesquiterpene Hydrocarbon | 1433 | 1432 |  |  |  |  | 0.05* |  |  |  |
| 282 | γ-Maaliene | cyclic Ester/Lactone | 1434 | 1430 | 0.02* | 0.02* | 0.09* |  |  |  |  |  |
| 283 | α-(E)-Bergamotene | Sesquiterpene Hydrocarbon | 1435 | 1432 |  |  |  |  | 1.12 |  |  |  |
| 284 | Aromadendrene | Sesquiterpene Hydrocarbon | 1436 | 1438 |  |  |  |  |  |  |  | 0.07* |
| 285 | Calarene | Sesquiterpene Hydrocarbon | 1438 | 1434 | 0.94* |  | 3.79* |  |  |  | 0.55* | 1.44* |
| 286 | β-Gurjunene | Sesquiterpene Hydrocarbon | 1439 | 1437 |  | 0.58* |  |  |  |  |  |  |
| 287 | α-Maaliene | Sesquiterpene Hydrocarbon | 1442 | 1438 |  |  | 0.06* |  |  |  |  |  |
| 288 | (Z)-β-Farnesene | Sesquiterpene Hydrocarbon | 1445 | 1439 |  |  |  |  | 0.20* |  |  |  |
| 289 | (E)-Cinnamyl acetate | Aromatic Ester | 1447 | 1448 |  |  |  |  |  |  | 0.01* |  |
| 290 | Geranylacetone | Monoterpene Ketone | 1448 | 1450 | 0.01* |  | 0.01* |  |  |  | 0.01* | 0.02* |
| 291 | epi-β-Santalene | Sesquiterpene Hydrocarbon | 1453 | 1446 |  |  |  |  | 0.04* |  |  |  |
| 292 | (E)-β-Farnesene | Sesquiterpene Hydrocarbon | 1454 | 1452 | 0.01 | 0.01 | 0.01* | 0.02* | 3.05 |  | 0.01* | 0.03* |
| 293 | Sesquisabinene | Sesquiterpene Hydrocarbon | 1457 | 1455 |  | 0.01* | 0.02* |  | 0.61* | 0.01* |  | 0.01* |
| 294 | β-Acoradiene | Sesquiterpene Hydrocarbon | 1462 | 1467 | 0.01* |  |  |  |  |  |  |  |
| 295 | α-Humulene | Sesquiterpene Hydrocarbon | 1463 | 1454 | 0.02* |  |  |  |  |  |  | 0.23* |
| 296 | γ-Decalactone | Cyclic Ester/Lactone | 1468 | 1469 |  |  |  |  |  |  | 0.31 |  |
| 297 | (E)-Ethylcinnamate | Aromatic Ester | 1470 | 1473 |  |  |  |  |  |  | 0.04* |  |
| 298 | Massoia lactone | Cyclic Ester/Lactone | 1476 | 1474 |  |  | 0.01* |  |  |  | 0.01* |  |
| 299 | γ-Muurolene | Sesquiterpene Hydrocarbon | 1481 | 1478 |  |  |  |  |  |  |  | 0.02* |
| 300 | Dodecanol | Aliphatic Alcohol | 1482 | 1476 |  |  |  | 0.01* |  |  |  |  |
| 301 | α-Curcumene | Sesquiterpene Hydrocarbon | 1485 | 1480 |  |  |  |  | 0.13* |  |  |  |
| 302 | (E)-β-Ionone | Monoterpene Ketone | 1486 | 1482 |  | 0.09 |  |  |  |  | 0.01* |  |
| 303 | 5-Methyl 2-phenyl-hex-2-enal | Aromatic Aldehyde | 1487 | 1485 |  |  |  |  |  |  | 0.01* |  |
| 304 | Germacrene D | Sesquiterpene Hydrocarbon | 1488 | 1480 | 0.01* |  | 0.01* |  | 0.8 |  |  | 0.01* |
| 305 | β-(E)-Bergamotene | Sesquiterpene Hydrocarbon | 1490 | 1483 |  |  |  |  | 0.17 |  |  |  |
| 306 | 1-Pentadecene | Hydrocarbon | 1494 | 1492 |  | 0.01* | 0.01* | 0.03* |  | 0.06* | 0.01* | 0.01* |
| 307 | β-Selinene | Sesquiterpene Hydrocarbon | 1496 | 1492 |  | 0.02* |  |  | 0.46 |  |  | 0.03* |
| 308 | α-Zingiberene | Sesquiterpene Hydrocarbon | 1499 | 1496 |  |  |  |  | 0.04* |  |  |  |
| 309 | n-Pentadecane | Hydrocarbon | 1500 | 1500 | 0.01* | 0.01* | 0.01* | 0.03* |  | 0.01* | 0.01* | 0.01* |
| 310 | α-Bulnesene | Sesquiterpene Hydrocarbon | 1504 | 1505 |  |  |  |  | 0.16* |  |  | 0.02* |
| 311 | α-Muurolene | Sesquiterpene Hydrocarbon | 1505 | 1497 |  |  |  |  |  |  | 0.04* | 0.01* |
| 312 | Isodaucene | Sesquiterpene Hydrocarbon | 1510 | 1504 |  |  |  |  | 0.51* |  |  |  |
| 313 | β-Bisabolene | Sesquiterpene Hydrocarbon | 1511 | 1508 | 0.06 | 0.06* | 0.47* |  | 2.81 | 0.01* | 0.04* | 0.12* |
| 314 | Myristicin | Oxygenated monoterpene Hydrocarbon | 1523 | 1520 |  |  |  |  |  | 0.04* |  |  |
| 315 | δ-Cadinene | Sesquiterpene Hydrocarbon | 1525 | 1518 |  | 0.06* |  |  |  |  |  | 0.03* |
| 316 | β-Sesquiphellandrene | Sesquiterpene Hydrocarbon | 1528 | 1523 |  |  | 0.01* |  | 0.31 |  |  |  |
| 317 | (E)-α-Bisabolene | Sesquiterpene Hydrocarbon | 1545 | 1540 |  |  |  |  | 0.25 |  |  |  |
| 318 | Benzyl hexanoate | Aromatic Ester | 1547 | 1547 |  |  |  |  |  | 0.01* |  |  |
| 319 | Dodecanoic acid | Acid | 1562 | 1581 |  | 0.01* |  |  |  |  |  |  |
| 320 | (E)-Nerolidol | Sesquiterpene Alcohol | 1563 | 1561 |  |  |  |  |  |  | 0.1 |  |
| 321 | Spathulenol | Sesquiterpene Alcohol | 1583 | 1576 |  |  | 0.01* |  |  |  |  | 0.01* |
| 322 | 1,2,3,4-Tetramethoxy-5-(2-propenyl)-benzene | Aromatic Ether | 1587 | 1591 |  |  |  |  |  | 0.01* |  |  |
| 323 | Caryophyllene oxide | Oxygenated Sesquiterpene | 1589 | 1587 |  | 0.01* | 0.01* |  | 2.32 |  |  | 0.07 |
| 324 | Ethyl dodecanoate | Aliphatic Ester | 1594 | 1598 |  | 0.01* |  |  |  |  | 0.01* | 0.01 |
| 325 | n-Hexadecane | Hydrocarbon | 1600 | 1600 |  |  |  | 0.01* |  | 0.01* | 0.01* |  |
| 326 | Carotol | Sesquiteprene Alcohol | 1610 | 1601 |  |  |  |  | 27.43 | 0.01* |  |  |
| 327 | n-Tetradecanal | Aliphatic Aldehyde | 1614 | 1614 |  | 0.01* |  | 0.01* |  |  | 0.01* |  |
| 328 | Humulene epoxide II | Oxygenated Sesquiterpene Hydrocarbon | 1619 | 1613 |  |  |  |  | 0.11 |  |  | 0.02* |
| 329 | Daucol | Sesquiterpene Alcohol | 1653 | 1642 |  |  |  |  | 0.75 |  |  |  |
| 330 | (9Z)-Tetradecen-1-ol | Aliphatic Alcohol | 1672 | 1665 |  |  |  | 0.01* |  |  |  |  |
| 331 | Apiole | Oxygenated sesquiterpene Hydrocarbon | 1678 | 1683 |  |  |  |  |  | 0.01* |  |  |
| 332 | γ-Dodecalactone | Cyclic ester/Lactone | 1679 | 1681 |  |  |  |  |  |  | 0.02 |  |
| 333 | n-Tetradecanol | Aliphatic Alcohol | 1684 | 1680 |  |  |  |  |  |  | 0.01* |  |
| 334 | n-Heptadecane | Hydrocarbon | 1700 | 1700 |  |  |  | 0.01* |  |  |  |  |
| 335 | Juniper camphor | Sesquiterpene Alcohol | 1708 | 1696 |  |  |  |  | 0.04 |  |  |  |
| 336 | Ethyl tetradecanoate | Aliphatic Ester | 1793 | 1794 |  |  |  |  |  |  | 0.01* |  |
| 337 | n-Octadecane | Hydrocarbon | 1800 | 1800 |  |  |  | 0.01* |  |  |  |  |
| 338 | Phytone | Sesquiterpene Ketone | 1842 | 1841 |  | 0.01* |  |  |  |  | 0.01* | 0.01* |
| 339 | Methyl pentadecyl ketone | Aliphatic Ketone | 1903 | 1908 |  | 0.01* |  |  |  |  |  |  |
| 340 | Methyl hexadecanoate | Aliphatic Ester | 1925 | 1925 |  | 0.01* |  |  |  |  | 0.01* | 0.01* |
| 341 | n-Hexadecanoic acid | Acid | 1962 | 1977 |  |  |  |  |  |  | 0.01* |  |
| 342 | Ethyl palmitate | Aliphatic Ester | 1994 | 1993 | 0.01* | 0.01* |  |  |  |  | 0.01* |  |
| 343 | Methyl linoleate | Aliphatic Ester | 2092 | 2093 |  | 0.01* |  |  |  |  | 0.01* |  |
| 344 | Methyl oleate | Aliphatic Ester | 2099 | 2098 |  |  |  |  |  |  | 0.01* |  |
| 345 | δ-Hexadecalactone | Cyclic ester/Lactone | 2137 | 2148 |  |  |  |  |  |  | 0.02* |  |
| 346 | Ethyl linoleate | Aliphatic Ester | 2160 | 2164 | 0.01* |  |  |  |  |  | 0.01* | 0.01* |

*Not reported in literature so far
